# Role of microRNAs in the age-associated decline of pancreatic beta cell function in rat islets

**DOI:** 10.1007/s00125-015-3783-5

**Published:** 2015-10-16

**Authors:** Ksenia Tugay, Claudiane Guay, Ana C. Marques, Florent Allagnat, Jonathan M. Locke, Lorna W. Harries, Guy A. Rutter, Romano Regazzi

**Affiliations:** Department of Fundamental Neurosciences, University of Lausanne, Rue du Bugnon 9, CH-1005 Lausanne, Switzerland; Department of Physiology, University of Lausanne, Lausanne, Switzerland; Institute of Biomedical and Clinical Sciences, University of Exeter Medical School, University of Exeter, Exeter, UK; Section of Cell Biology and Functional Genomics, Division of Diabetes, Endocrinology and Metabolism, Imperial College London, London, UK

**Keywords:** Ageing, Apoptosis, Beta cell, Diabetes, Insulin secretion, MicroRNA, Pancreatic islet, Proliferation

## Abstract

**Aims/hypothesis:**

Ageing can lead to reduced insulin sensitivity and loss of pancreatic beta cell function, predisposing individuals to the development of diabetes. The aim of this study was to assess the contribution of microRNAs (miRNAs) to age-associated beta cell dysfunction.

**Methods:**

The global mRNA and miRNA profiles of 3- and 12-month-old rat islets were collected by microarray. The functional impact of age-associated differences in miRNA expression was investigated by mimicking the observed changes in primary beta cells from young animals.

**Results:**

Beta cells from 12-month-old rats retained normal insulin content and secretion, but failed to proliferate in response to mitotic stimuli. The islets of these animals displayed modifications at the level of several miRNAs, including upregulation of miR-34a, miR-124a and miR-383, and downregulation of miR-130b and miR-181a. Computational analysis of the transcriptomic modifications observed in the islets of 12-month-old rats revealed that the differentially expressed genes were enriched for miR-34a and miR-181a targets. Indeed, the induction of miR-34a and reduction of miR-181a in the islets of young animals mimicked the impaired beta cell proliferation observed in old animals. mRNA coding for alpha-type platelet-derived growth factor receptor, which is critical for compensatory beta cell mass expansion, is directly inhibited by miR34a and is likely to be at least partly responsible for the effects of this miRNA.

**Conclusions/interpretation:**

Changes in the level of specific miRNAs that occur during ageing affect the proliferative capacity of beta cells. This might reduce their ability to expand under conditions of increased insulin demand, favouring the development of type 2 diabetes.

**Electronic supplementary material:**

The online version of this article (doi:10.1007/s00125-015-3783-5) contains peer-reviewed but unedited supplementary material, which is available to authorised users.

## Introduction

Insulin secretion from pancreatic beta cells plays a central role in blood glucose homeostasis and metabolism control. Reduced sensitivity of insulin target tissues and a consequent rise in insulin demand is normally compensated by expansion of beta cells and an increase in their secretory activities. Failure in this compensatory process results in the release of insufficient insulin to cover the organism’s needs and the development of type 2 diabetes [1]. Ageing is a risk factor for several metabolic diseases, including type 2 diabetes. Indeed, ageing can affect both insulin secretion and insulin action, and predisposes to glucose intolerance and diabetes [2]. Moreover, ageing is associated with impaired proliferation and increased sensitivity of beta cells to apoptosis [3], reducing their capacity to cope with an insulin-resistance state. At present, the mechanisms underlying these phenomena are not fully understood, but changes in the expression of genes coding for key proteins have been reported to be involved in the age-associated decline of beta cell function.

MicroRNAs (miRNAs) are short non-coding RNAs that bind to the 3′ untranslated region (UTR) of target mRNAs, causing translational repression and/or messenger degradation [4]. During the past decade, several studies have demonstrated the involvement of miRNAs in the regulation of beta cell function and survival [5–7]. Although the role of miRNAs in the regulation of beta cell activities has been investigated in various conditions, including pregnancy and obesity [8–11], the potential contribution of these molecules to age-associated beta cell impairment has not, as yet, been explored.

In this study, we compared the islet miRNA profiles of 3- and 12-month-old rats. Several differentially expressed miRNAs were identified and their roles in beta cell secretion, proliferation and survival upon chronic exposure to proapoptotic conditions were systematically investigated.

## Methods

### Chemicals

Prolactin, IL-1β, exendin-4 and platelet-derived growth factor (PDGF)-AA were obtained from Sigma-Aldrich (St Louis, MO, USA). TNF-α was obtained from Enzo Life Sciences (Lausanne, Switzerland) and IFNγ was obtained from R&D Systems (Minneapolis, MN, USA).

### Animals

Wistar rats were from Janvier Laboratories (Le Genest St Ile, France). All procedures were approved by the Swiss Veterinary Office and were in accordance with National Institutes of Health guidelines.

### Islet isolation and cell culture

Islets were isolated by collagenase digestion [12] and cultured in RPMI 1640 medium (Invitrogen, Carlsbad, CA, USA) [9]. Dissociated islet cells were obtained by trypsin digestion [8]. Human islets were received from the Cell Isolation and Transplantation Center (University of Geneva, Geneva, Switzerland). Dissociated human islet cells were cultured in CMRL medium (Invitrogen) [9]. The rat insulin-secreting cell line INS832/13 was cultured as previously described [8].

### Profiling of miRNA and mRNA

RNA was isolated using the miRNeasy Kit (Qiagen, Hombrechtikon, Switzerland). MiRNA expression profiling was carried out using Agilent Technologies (Santa Clara, CA, USA) miRNA Microarrays [9]. Profiling of mRNAs was carried out by Arraystar (Rockville, MD, USA).

### Measurement of miRNA and mRNA expression

MiRNA expression was assessed using the miRCURY LNA Universal RT miRNA PCR kit (Exiqon, Vedbaek, Denmark). Measurements of mRNA levels were performed by quantitative real-time PCR (qPCR; Bio-Rad, Reinach, Switzerland) with custom-designed primers (Microsynth, Balgach, Switzerland) (see Electronic Supplementary Material [ESM] Methods). MiRNA expression was normalised to the level of U6 or miR-7, while mRNA expression was normalised to 18S. The level of selected miRNAs in cadaveric human islets from 11 normoglycaemic donors, and their association with age, was assessed in our previously published global miRNA profiling dataset [13].

### Transfection and modulation of miRNA levels

INS832/13 cells and primary rat or human dissociated islet cells were transfected with Lipofectamine 2000 (Invitrogen), either with RNA oligonucleotide duplexes (Eurogentec, Seraing, Belgium) corresponding to the sequence of the miRNAs (overexpression) or with single-stranded miScript miRNA Inhibitors (Qiagen) that specifically inhibit endogenous miRNAs [9]. A custom-designed small interfering RNA duplex directed against green fluorescent protein (Eurogentec) and a miScript reference miRNA (Qiagen) were used as respective controls.

### Insulin secretion

The insulin content of and insulin secretion by dissociated rat islet cells were measured by ELISA [14].

### Cell death assessment

Rat and human islet cells were incubated with 1 μg/ml Hoechst 33342 (Invitrogen) for 1 min. At least 10^3^ cells/condition were analysed under fluorescence microscopy (AxioCam MRc 5, Zeiss, Feldbach, Switzerland) to score the fraction of cells displaying pycnotic nuclei.

### Proliferation assay

Cells were cultured on poly-l-lysine-coated glass coverslips. They were fixed with cold methanol, permeabilised with 0.5% saponin (Sigma-Aldrich) and exposed for 1 h to antibodies against Ki67 (1:400) (Abcam, Cambridge, UK) and insulin (1:10,000) (Millipore, Zug, Switzerland). Coverslips were then incubated for 1 h with anti-rabbit Alexa-Fluor-488 and anti-mouse Alexa-Fluor-555 antibodies (Invitrogen). Images were collected on an AxioVision fluorescence microscope.

### Luciferase assay

A luciferase reporter construct was generated by inserting 212 nucleotides of the 3′ UTR sequence of rat *Pdgfra* surrounding the putative binding site of miR-34a between the XhoI and EcoRI sites of psiCHECK-1 (ESM Methods). Luciferase activity was measured using a dual-luciferase reporter assay (Promega, Madison, WI, USA). Firefly luciferase activity was normalised for transfection efficiency with the SV40-driven *Renilla* activity generated by pGL3-Basic (Promega).

### Western blotting

Cells were lysed in Laemmli buffer. Lysates were resolved by SDS-PAGE, transferred to a PVDF membrane and detected using antibodies against PDGF receptor α (PDGFRα) (catalogue no. 3174; Cell Signaling Technology, Danvers, MA, USA) and α-tubulin (Fluka Chemie, Buchs, Switzerland) [15]. After 1 h incubation at room temperature with horseradish peroxidase-conjugated secondary antibodies (Fluka Chemie), membranes were revealed by chemiluminescence (Immobilon, Millipore) using the ChemiDoc XRS+ System (Bio-Rad Laboratories).

### miRNA target enrichment analysis

For each differentially expressed miRNA, we estimated the median number of miRNA recognition elements (M_obs_) predicted, using TargetScan (version 6.2) [16], in the 3′ UTR (rn6, downloaded from UCSC [17] on 10 April 2015) of *n* up- or downregulated genes. To obtain an empirical *p* value associated with each M_obs_, we independently estimated 1,000 times the median TargetScan predicted density of miRNA recognition elements for N regions of matching length randomly sampled from the 3′ UTRs of rat islet mRNAs that were not differentially expressed.

### Statistical analysis

Statistical differences were tested using Student’s *t* test or for multiple comparisons, with ANOVA followed by a post hoc Dunnett test, with a discriminating *p* value of 0.05 (SAS statistical package, Cary, NC, USA).

## Results

**Fig. 1:**
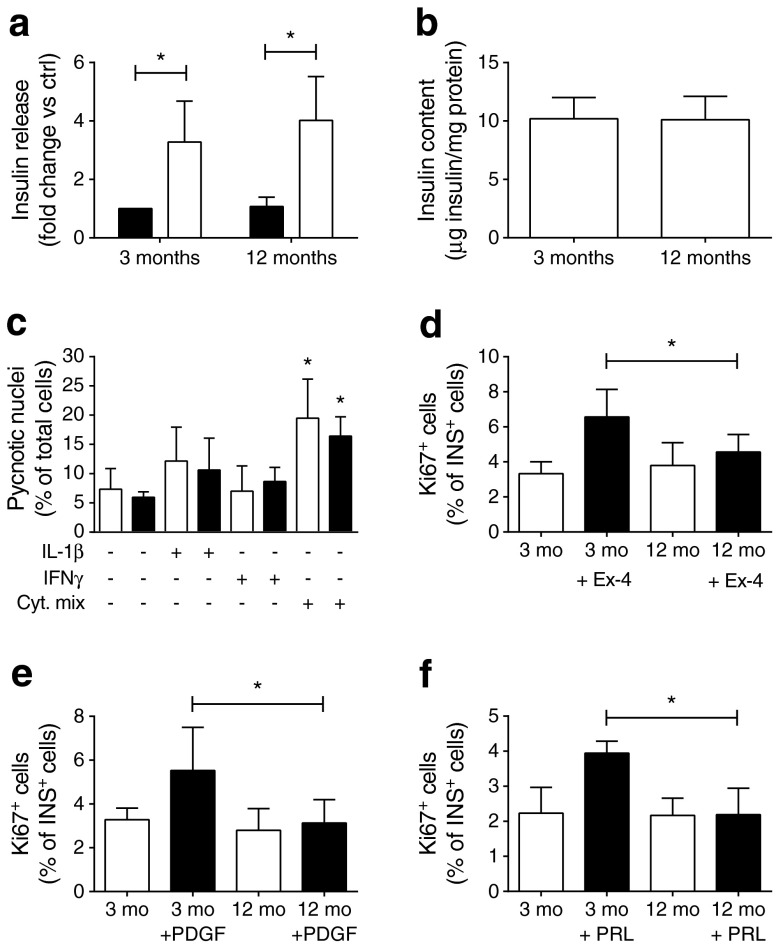
Insulin secretion, apoptosis and proliferation in islets of aged rats. (**a**) Insulin secretion of rat islet cells in response to 2 mmol/l (black bars) or 20 mmol/l (white bars) glucose. Results are represented as fold change compared with 2 mmol/l glucose in 3-month-old rats. (**b**) Insulin content was normalised to total protein content. (**c**) Apoptosis was measured in the islet cells of young (white bars) and aged (black bars) animals treated with IL-1β (10 ng/ml), IFNγ (300 ng/ml) or with a cytokine (Cyt.) mix (0.1 ng/ml IL-1β, 10 ng/ml TNF-α and 30 ng/ml IFNγ). *p < 0.05 vs untreated rats of the same age. (**d–f**) Beta cell proliferation in rat islet cells of 3- and 12-month-old animals treated with exendin-4 (Ex-4; 100 nmol/l) (**d**), PDGF-AA (50 ng/ml) (**e**) or prolactin (PRL; 500 ng/ml) (**f**) for 5 days was assessed by staining with anti-Ki67 and anti-insulin antibodies. Results are means ± SD of at least three independent experiments. *p < 0.05 by ANOVA and Dunnett’s post hoc test. Ctrl, control; mo, month

As observed by others [18, 19], 12-month-old male Wistar rats displayed an increase in body weight but no difference in blood glucose levels compared with 3-month-old animals (ESM Fig. 1). In agreement with these observations, insulin content and glucose-stimulated insulin secretion from islets isolated from older animals were comparable with those of younger animals (Fig. 1a, b) and the sensitivity of islet cells to apoptosis was unchanged (Fig. 1c). However, in contrast to beta cells from young animals, those isolated from 12-month-old rats displayed no proliferative response to exendin-4, PDGF or prolactin (Fig. 1d–f).

To determine whether these phenotypic traits were linked to transcriptomic differences, we used microarray analysis to compare the global mRNA profiles of the islets isolated from young and old rats. We detected 632 upregulated and 397 downregulated genes (fold change >2.0; nominal *p* < 0.05, *n* = 3) in the islets of 12-month-old rats (ESM Tables 1, 2; microarray data are deposited in Gene Expression Omnibus under the accession code GSE72466). These changes are unlikely to reflect differences in islet composition, since the islet β-cell content is not modified in 12-month-old animals [20]. Among the upregulated mRNAs, pathway analysis revealed an enrichment of genes involved in insulin secretion, mitogen-activated protein kinase signalling and MODY (ESM Table 3). Changes in the expression of selected genes belonging to these pathways were verified by qPCR (ESM Fig. 2). In particular, we confirmed the induction of genes involved in insulin secretion (such as the calcium-channel subunits *Cacna1c* and *Cacna1d*) and MODY (*Slc2a2* and *Neurod1*), and encoding the transcription factor p53 (*Tp53*).

**Fig. 2:**
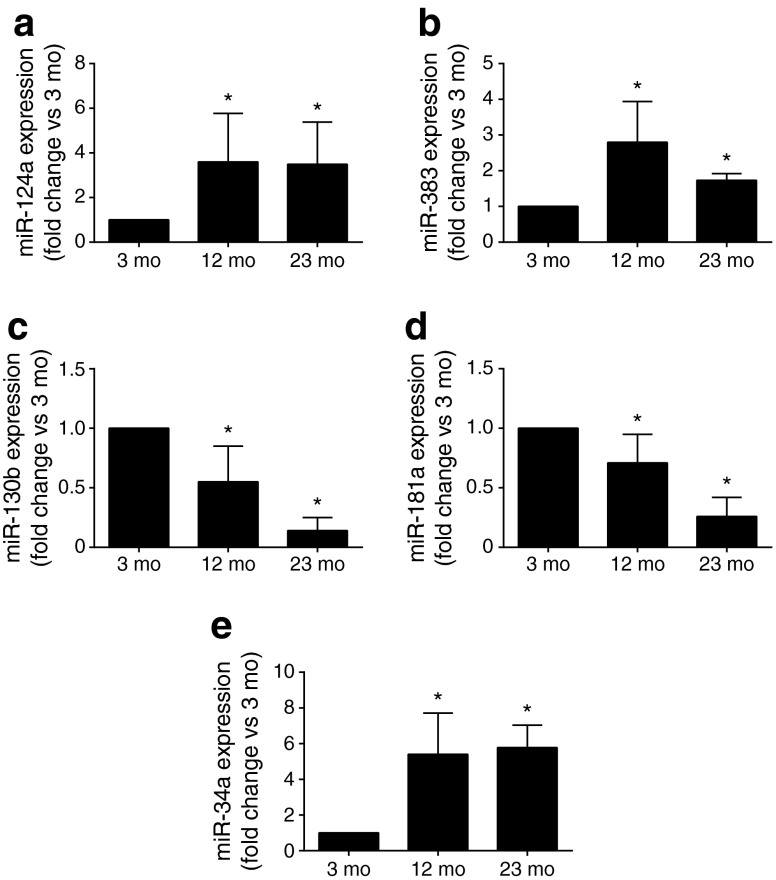
Islet miRNA expression in rats of different ages. miRNA expression was measured by qPCR. Results are presented as fold change vs the level in islets of 3-month-old animals and represent means ± SD; *n* = 5. *p < 0.05 vs 3-month-old mice by ANOVA and Dunnett’s post hoc test. Mo, month

MiRNAs are major regulators of gene expression and are involved in senescence and other age-associated phenomena [21–23]. To study the potential contribution of these RNAs to age-associated beta cell dysfunction, we analysed by microarray modifications in the islet miRNA profile of 12-month-old animals. Out of 307 detected miRNAs, 69 displayed changes in their expression level (nominal *p* < 0.05) (ESM Table 4; GSE72466). Changes in the level of all miRNAs displaying an adjusted *p* < 0.05 were verified by qPCR. We indeed confirmed the upregulation of miR-124a and miR-383 and the downregulation of miR-181a and miR-130b observed by microarray (Fig. 2a–d). The modifications in the levels of these miRNAs persisted and were in some cases exacerbated in the islets of 23-month-old animals (Fig. 2a–d). Interestingly, miR-124a plays a role in beta cell development [24] and insulin secretion [25], while miR-383 is differentially expressed in *db/db* mice and in mice fed a high-fat diet, two models of type 2 diabetes [9]. We were unable to detect reproducible changes in the levels of miR-29b, miR-129-1* (currently annotated miR-129-1-3p), miR-484 and miR-488 (ESM Fig. 3). Thus, these miRNAs were not further investigated.

**Fig. 3:**
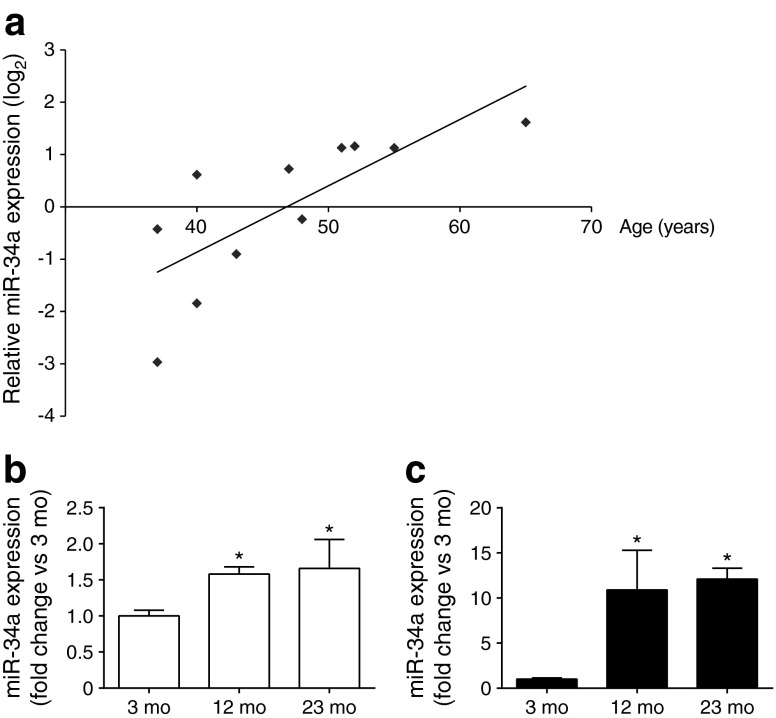
Age-associated changes in miR-34a expression in human islets and in other rat organs. RNA was isolated from the islets of human donors of various ages (**a**) and from the brain (**b**) and liver (**c**) of rats aged 3, 12 and 23 months. (**a**) The miR-34a level was positively correlated with the age of human islet donors. miRNA expression was measured using TaqMan Low Density Array plates (Life Technologies, Zug, Switzerland) and a global mean normalisation strategy to control for RNA input. Relative miR-34a levels were calculated using the mean ΔCt of all 11 samples and log_2_ transformed for normality. miR-34a expression in brain (**b**) and liver (**c**) of rats. The data are expressed as fold change vs the level of expression in 3-month-old rats. (**b**, **c**) Data represent means ± SD *p < 0.05 vs 3-month-old rats by ANOVA and Dunnett’s post hoc test. Mo, month

MiR-34a increases in different tissues during ageing [26, 27] and plays a role in islet cell survival and insulin secretion [28]. Although they did not reach statistical significance, the microarray data suggested a possible increase of miR-34a, miR-34b and miR-34c in the islets of old animals (ESM Table 4). Indeed, analysis of miR-34a expression by qPCR confirmed an upregulation of this miRNA in the islets of both 12- and 23-month-old rats (Fig. 2e). Islet miR-34a expression was also positively correlated with age in human islets from normoglycaemic donors of different ages (*r*^2^ = 0.58, *p* = 0.007) (Fig. 3a). In contrast, we did not detect significant (*p* > 0.05) correlations between the levels of the other differentially expressed miRNAs and the age of islet donors (data not shown). In this case, the effect of ageing observed in congenic animals may have been masked by genetic variations in the human donors or by other confounding factors such as sex or BMI [29].

We then verified whether the observed modifications in miRNA expression are specific for islet cells. Most of the selected miRNAs displayed expression profiles in insulin target tissues (liver, skeletal muscle and adipose tissue) and brain that differed from those of islets (ESM Table 5). In contrast, and in line with the literature [26, 27], miR-34a was upregulated in the liver and brain of older rats relative to 3-month-old rats (Fig. 3b, c).

**Fig. 4:**
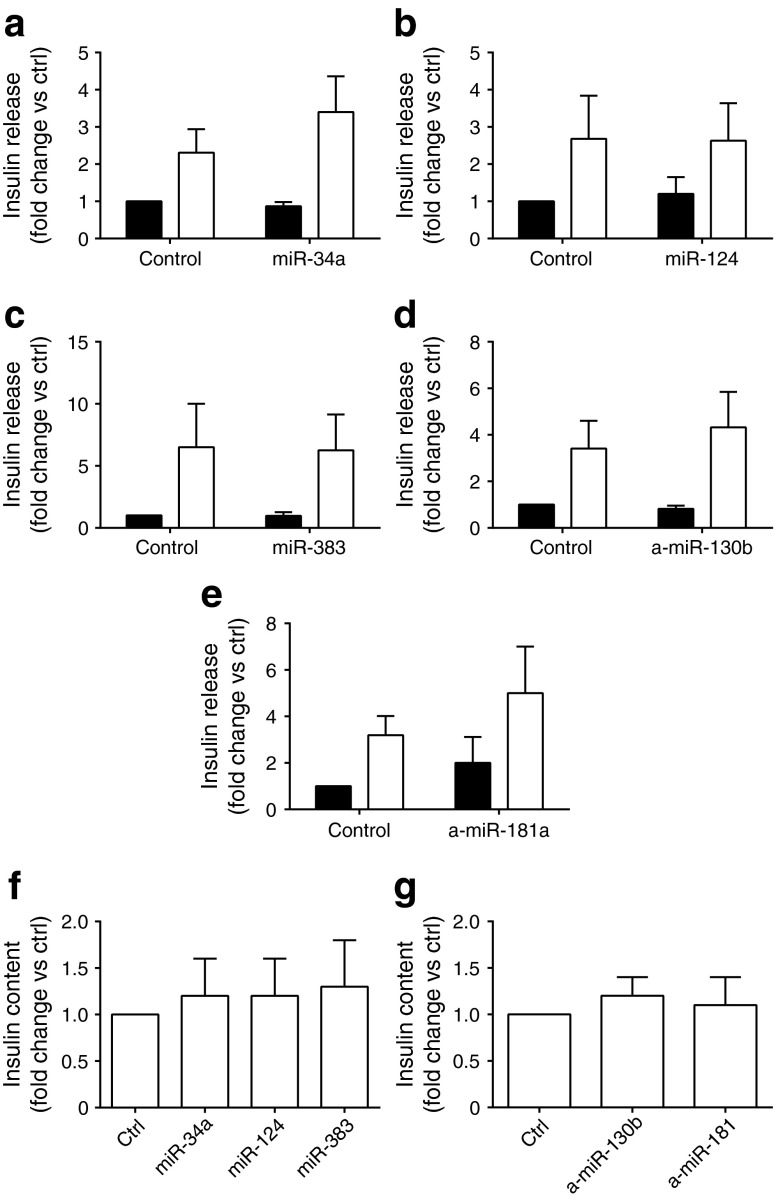
Impact of changes in miRNA expression on insulin secretion and content. Dissociated rat islet cells were transfected with oligonucleotides leading to overexpression (**a**–**c**) or downregulation (**d**, **e**) of the indicated miRNAs. Insulin secretion (**a**–**e**) and insulin content (**f**, **g**) in response to 2 mmol/l (black bars) or 20 mmol/l (white bars) glucose was measured 48 h post transfection. Insulin release is expressed as fold change vs 2 mmol/l glucose in the control (ctrl) condition. Data represent means ± SD

**Fig. 5:**
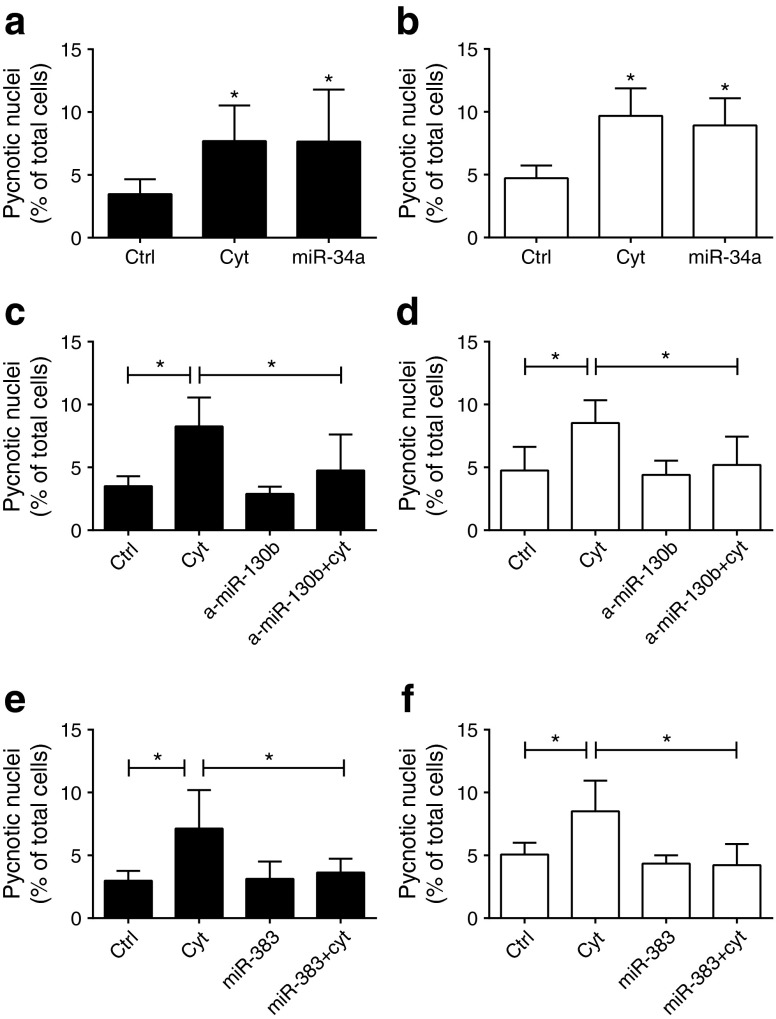
Impact of changes in miRNA expression on apoptosis. Dispersed rat (**a**, **c**, **e**) and human (**b**, **d**, **f**) islet cells were transfected with oligonucleotides leading to overexpression (**a**, **b**, **e**, **f**) or downregulation (**c**, **d**) of the indicated miRNAs. Apoptosis was assessed by scoring the cells displaying pycnotic nuclei. Incubation of cells transfected with control (ctrl) oligonucleotides during 24 h with a mix of proinflammatory cytokines (10 ng/ml TNF-α, 0.1 ng/ml IL-1β and 30 ng/ml IFNγ; cyt) was used as a positive control for apoptosis. Results are the means ± SD of three or four independent experiments. *p < 0.05 (vs control for **a**, **b**) by ANOVA and Dunnett’s post hoc test

To investigate the impact of the selected miRNAs on beta cell activities, islet cells of 3-month-old animals were transfected with miRNA mimics or anti-miRs to reproduce the changes in their expression that occur during ageing (ESM Fig. 4). Modifications in the levels of these miRNAs did not affect insulin content or insulin secretion (Fig. 4). Since ageing is characterised by increased susceptibility of beta cells to apoptotic stimuli [3], we investigated the impact of these miRNAs on beta cell survival. We found that overexpression of miR-34a triggers apoptosis of rat (Fig. 5a) and human islet cells (Fig. 5b). Downregulation of miR-130b and overexpression of miR-383 did not affect apoptosis of rat (Fig. 5c, e) or human (Fig. 5d, f) islet cells, whereas they exerted a protective effect when the cells were treated with cytokines. Downregulation of miR-181a or overexpression of miR-124a modified neither the basal apoptotic rate nor survival in the presence of cytokines (ESM Fig. 5).

**Fig. 6:**
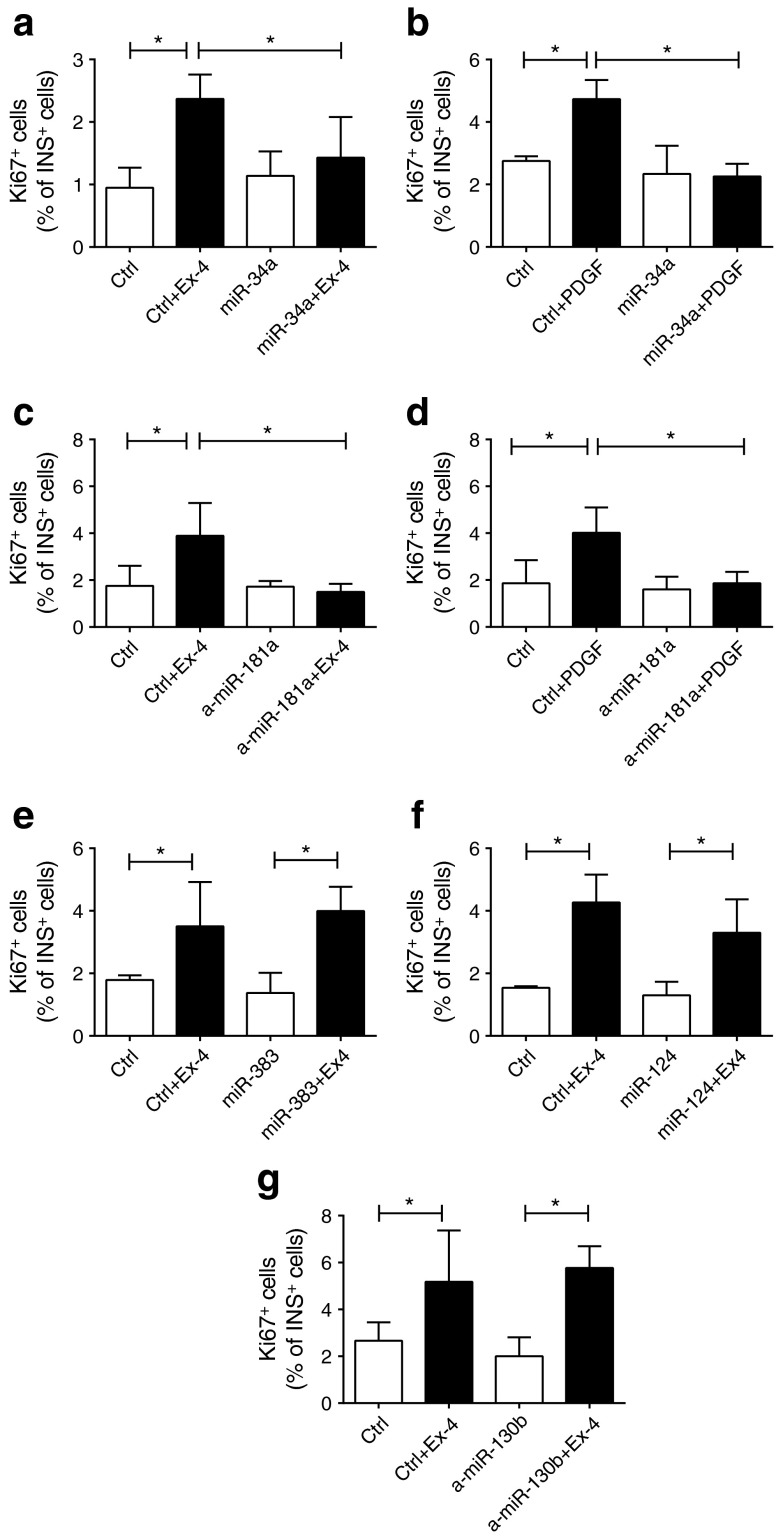
Impact of changes in miRNA expression on beta cell proliferation. Rat islet cells were transfected with oligonucleotides leading to overexpression (**a**, **b**, **e**, **f**) or downregulation (**c**, **d**, **g**) of the indicated miRNAs and treated with exendin-4 (Ex-4; 100 nmol/l) or PDGF-AA (50 ng/ml) (black bars). Beta cell proliferation was assessed 48 h later by anti-Ki67 and anti-insulin staining. The results represent the means ± SD of three to six independent experiments. *p < 0.05 by ANOVA and Dunnett’s post hoc test. Ctrl, control

We next assessed the impact of the selected miRNAs on beta cell proliferation. We observed that overexpression of miR-34a (Fig. 6a, b) or downregulation of miR-181a (Fig. 6c, d) did not affect basal beta cell proliferation, but did inhibit proliferation stimulated by exendin-4 or PDGF-AA. In contrast, beta cell proliferation was unaffected by overexpression of miR-383 and miR-124a or downregulation of miR-130b (Fig. 6e–g).

**Fig. 7:**
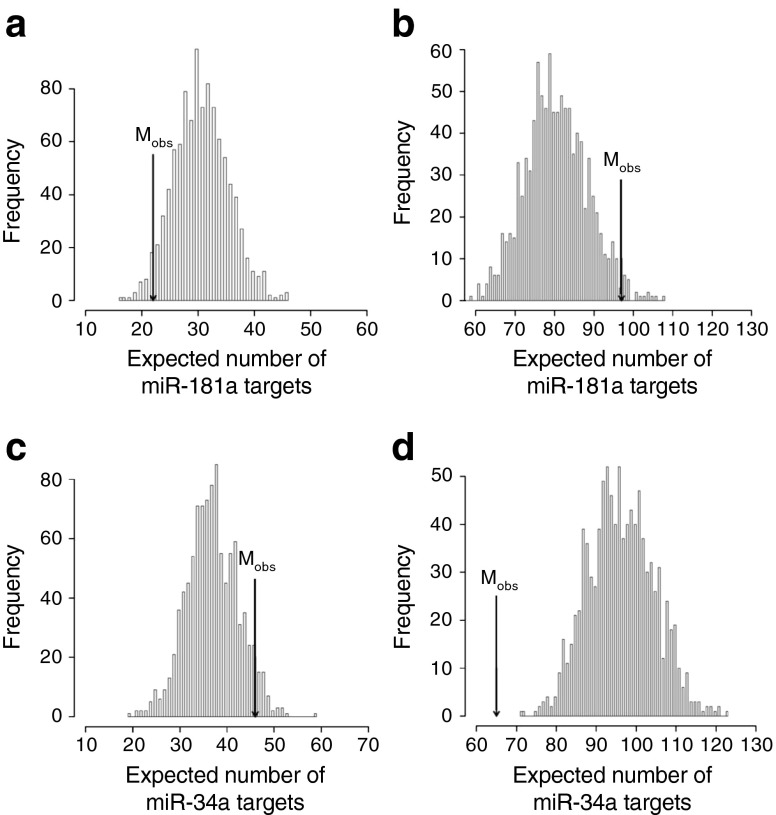
Number of miR-34a and miR-181a recognition elements in the 3′ UTR of differentially expressed mRNAs in islets of 12-month-old rats. The median number of recognition elements (M_obs_) for miR-181a (**a**, **b**) and miR-34a (**c**, **d**) predicted in the 3′ UTR of all down- (**a**, **c**) and upregulated (**b**, **d**) mRNAs in ageing (arrows) was compared with a null distribution of the median number of predicted recognition elements obtained for 1,000 randomly sampled sets of 3′ UTRs from mRNAs expressed in rat islets. *p* is the associated empirical *p* value and corresponds to the proportion of values in the null distribution that are either smaller or larger (left and right side of the distribution, respectively) than M_obs_. (**a**) *p* = 0.025; (**b**) *p* = 0.023; (**c**) *p* = 0.071; (**d**) *p* = 0

To unravel the mechanisms underlying the effects of miR-34a and miR-181a on age-associated beta cell dysfunction, we searched for potential targets within genes expressed in the islets of aged animals. Analysis of the protein-coding genes differentially expressed in islets of 12-month-old rats revealed significant enrichment for potential targets of miR-181a in the upregulated genes (Fig. 7b, ESM Table 6) and depletion of predicted targets in the downregulated genes (Fig. 7a, ESM Table 7). Moreover, the upregulated genes were significantly depleted from miR-34a targets (Fig. 7d, ESM Table 6) and, although the values did not reach statistical significance (*p* < 0.071), the downregulated genes tended to be enriched in the predicted targets of this miRNA (Fig. 7c, ESM Table 7). These observations suggest that age-associated changes in miR-181a and miR-34a levels contribute to the gene expression differences observed in the islets of aged rats.

**Fig. 8:**
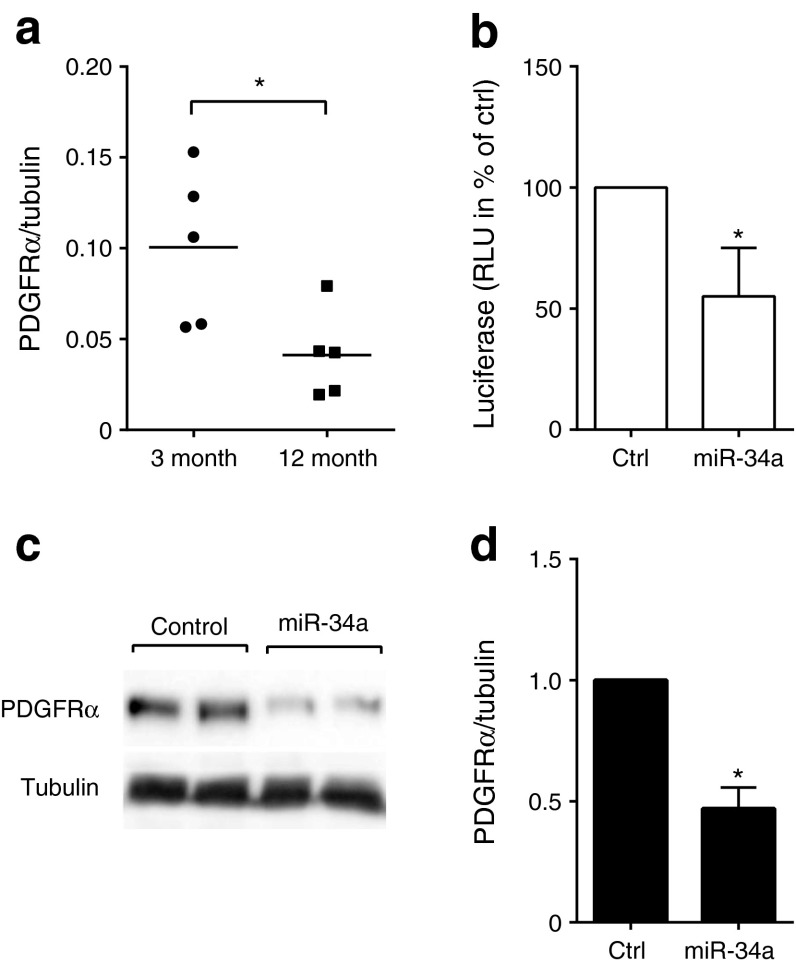
PDGFRα is a direct miR-34a target. (**a**) The levels of PDGFα in the islets of 3- and 12-month-old rats was determined by western blotting. The dot blot indicates the values of each of the tested rats (*p < 0.05 by Student’s *t* test). (**b**) INS832/13 cells were co-transfected with miR-34a mimic and with a reporter construct containing the 3′ UTR of PDGFRα. Luciferase activity was measured 2 days later. The results are expressed in relative light units (RLU) and are means ± SD (*n* = 3). *p < 0.05 vs control (Ctrl) by Student’s *t* test. (**c**, **d**) Dissociated rat islet cells were transfected with a control oligonucleotide or with the miR-34a mimic. Two days later, the levels of PDGFRα were analysed by western blotting. The figure shows a representative blot (**c**) and band quantification (**d**) from four independent experiments. Data are means ± SD. *p < 0.05 vs control

PDGFRα, a receptor whose decrease contributes to impaired age-associated beta cell proliferation [30], is a member of the beta cell ‘disallowed gene’ family [31]. Western blot analysis revealed that the levels of this receptor are reduced in the islets of 12-month-old rats (Fig. 8a). PDGFRα is a predicted target of miR-34a (www.targetscan.org) and is regulated by this miRNA in cancer cells [32]. Although *Pdgfra* expression is upregulated in islet beta cells after DICER deletion and global miRNA suppression [33], its targeting by miR-34a in beta cells has not been explored. To experimentally verify PDGFRα as a target for miR-34a in these cells, INS832/13 cells were transfected with a luciferase construct containing the 3′ UTR of *Pdgfra* mRNA. Overexpression of miR-34a resulted in reduced luciferase activity, confirming that *Pdgfra* is indeed a direct target of miR-34a (Fig. 8b). Moreover, western blot analysis of rat islet cells overexpressing miR-34a confirmed that the miRNA reduces the levels of PDGFRα (Fig. 8c, d).

## Discussion

Ageing is a risk factor for the development of type 2 diabetes. In fact, it is associated with a progressive decrease in insulin sensitivity and a decline in beta cell function [2]. The mechanisms underlying these effects are not fully understood, but are likely to involve changes in gene expression. Here, we investigated the transcriptomic modifications occurring in islets of 12-month-old rats. In agreement with other studies [34], we did not observe alterations in insulin secretion or apoptosis in the islets of these animals, suggesting that beta cells preserve the capacity to accomplish their basic tasks. However, beta cells from 12-month-old rats were unable to respond to mitogens. The loss of beta cell proliferation is probably not a significant handicap under normal conditions, but might constitute a major obstacle to the achievement of blood glucose homeostasis under conditions of insulin resistance requiring compensatory beta cell mass expansion.

Our data indicate that the expression of several protein-coding genes and miRNAs is already modified in the islets of 12-month-old rats. Some of these transcriptomic changes are likely to have a positive impact on beta cell function. For instance, we detected the upregulation of genes involved in insulin release, such as those coding for calcium-channel subunits [35–37] and the glucose transporter GLUT-2 (Slc2a2) [38]. These data suggest the existence of compensatory mechanisms operating to maintain efficient insulin secretion in 12-month-old animals.

In addition to differences in protein-coding genes, the islets of 12-month-old rats also displayed modifications in the miRNA profile. When changes in the levels of selected miRNAs were reproduced in islet cells of young animals, none of them affected insulin biosynthesis or secretion. In cell lines, overexpression of miR-124a and miR-34a has previously been reported to inhibit insulin secretion [25, 28, 39] but this observation was not confirmed in another study, although glucose-induced Ca^2+^ fluxes were affected by miR-124a overexpression [24]. These discrepant findings might be due to differences in the level of overexpression achieved after cell transfection. Moreover, because of transcriptomic differences, these miRNAs might have distinct impacts on insulin secretion in primary beta cells and tumoral beta cell lines. These miRNAs reduce the expression of key components of the machinery of insulin exocytosis [25, 28, 39]. Thus, although overexpression in beta cells of young animals did not significantly impair insulin release, we cannot exclude the possibility that the presence of higher levels of miR-34a or miR-124a would exacerbate the secretory decline of ageing beta cells.

Several of the miRNAs displaying expression changes in ageing animals affect the survival of beta cells. Indeed, downregulation of miR-130b or induction of miR-383 improved the survival of beta cells under proapoptotic conditions. Similar protective effects have already been reported for other miRNAs under insulin-resistance conditions [8, 9]. The changes in the levels of these miRNAs might be sufficient to compensate for the proapoptotic effect elicited by the concomitant upregulation of miR-34a. This suggests that a balance between the action of proapoptotic and antiapoptotic miRNAs prevents a net loss of beta cells in 12-month-old rats.

The most striking defect observed in beta cells of 12-month-old rats was the loss of proliferation in response to mitotic stimuli. Interestingly, overexpression of miR-34a or blockade of miR-181a was sufficient to reproduce this phenotypic trait in the islets of younger animals. The mechanism through which the downregulation of miR-181a contributes to the age-associated impairment of beta cell proliferation remains to be established. However, analysis of differentially expressed genes in the islets of 12-month-old rats revealed an enrichment of miR-181a targets in the upregulated mRNAs and a reduction in those that were downregulated. Thus, attenuation of the repressive activity of this miRNA appears to contribute to transcriptomic modifications occurring in the islets of ageing animals. The islet gene-expression profile of ageing animals also appears to be influenced by the induction of miR-34a. Indeed, the 3′ UTR of the upregulated genes was depleted from potential recognition sequences for this miRNA, and the putative miR-34a targets tended to be more frequent in genes downregulated in ageing. Some of the targets of miR-34a might be directly involved in the proliferative defect. Indeed, we demonstrated that the mRNA coding for PDGFRα is directly targeted by miR-34a. PDGFR signalling plays a critical role in postnatal beta cell mass expansion and in beta cell regeneration [30]. Thus, the translational repression of *Pdgfra* exerted by the induction of miR-34a is likely to contribute to the loss of proliferative capacity observed during ageing.

The increase in the level of miR-34a in the islets of ageing rats is probably linked to the activation of p53 signalling. Indeed, we have previously reported that, in insulin-secreting cells, this transcription factor binds to the promoter of miR-34a and triggers expression of the miRNA [40]. The p53 pathway is induced by a variety of stresses and plays a pivotal role in cellular senescence and metabolic homeostasis [41, 42]. p53 and miR-34a are linked in a positive-feedback loop to sirtuin-1 (SIRT1). Indeed, p53 induces the expression of miR-34a and the miRNA targets and represses SIRT1, preventing SIRT1-mediated deacetylation of p53 and, in turn, promoting the activity of the transcription factor [43]. Consistent with this model, we found that increases in p53 and miR-34a in the islets of ageing animals were indeed associated with reduced levels of SIRT1.

In addition to being upregulated in islets, the level of miR-34a was also elevated in the liver and brain of old rats, pointing to a general role for this miRNA in the ageing process. Moreover, the expression of this miRNA has been found to be abnormally elevated in islets and insulin target tissues of obese animals [40, 44, 45], contributing to beta cell failure and insulin resistance. Being at the crossroad between ageing and metabolic imbalance, these findings point to miR-34a induction as an important risk factor for the development of type 2 diabetes. This will particularly hold true for individuals expressing single nucleotide polymorphisms within the precursor of miR-34a that result in increased expression of this miRNA [46].

In this study, we identified a group of islet miRNAs that display expression changes during ageing and are likely to contribute to the progressive failure of beta cells to compensate for insulin resistance. A better understanding of the role and the mode of action of these miRNAs in beta cells might open the way to the development of new strategies for the prevention and/or treatment of type 2 diabetes.

## Electronic supplementary material

ESM Methods (PDF 163 kb)

ESM Fig. 1 (PDF 78 kb)

ESM Fig. 2 (PDF 83 kb)

ESM Fig. 3 (PDF 11 kb)

ESM Fig. 4 (PDF 85 kb)

ESM Fig. 5 (PDF 80 kb)

ESM Table 1 (PDF 2147 kb)

ESM Table 2 (PDF 1457 kb)

ESM Table 3 (PDF 121 kb)

ESM Table 4 (PDF 1047 kb)

ESM Table 5 (PDF 349 kb)

ESM Table 6 (PDF 303 kb)

ESM Table 7 (PDF 237 kb)

## Abbreviations

miRNA: MicroRNA
M_obs_: Median number of miRNA recognition elements
PDGF: Platelet-derived growth factor
PDGFRα: Platelet-derived growth factor receptor α
qPCR: Quantitative real-time PCR
SIRT1: Sirtuin-1
UTR: Untranslated region
