# Supplementary material for: Role of microRNAs in the age-associated decline of pancreatic beta cell function in rat islets
Source: Diabetologia. 2015 Oct 16;59(1):161–9. doi: 10.1007/s00125-015-3783-5 (PMC4670458; doi:10.1007/s00125-015-3783-5)
Supplement: Supplementary file 1 — (PDF 163 kb) [file 125_2015_3783_MOESM1_ESM.pdf]

Supplementary methods

A) Sequence of the primers used for qRT-PCR

Gene ID	Forward primers	Reverse primers
CACNA1C	5'-CCA TTG CCT CCG AAC ACT AC-3'	5'-GCG GTT GAA GAC GGA TAC AA-3'
CACNA1D	5'-CCC AAT GGA GGC ATC ACT AA-3'	5'-ATG GCC ACT CCC ATC CTA TC-3'
NeuroD1	5'-GGA TGA TCA AAA GCC CAA GA-3'	5'-GCA GGG TAC CAC CTT TCT CA-3'
Slc2a2	5'-TTC CTC GCC TGG TTC TAC T -3'	5'-GAA ACC GAC GTG GGT AAC A-3'
Sirt-1	5'-CCA GAT CCT CAA GCC ATG TT-3'	5'-GAT CCT TTG GAT TCC TGC AA-3'
TP53	5'-GGC CTC TGT CAT CTT CCG TC-3'	5'-TAC CAG GTG GAG GTG TGG AG-3'
18S	5'-GCA ATT ATT CCC CAT GAA CG-3'	5'-GGC CTC ACT AAA CCA TCC AA-3'

B) Sequence introduced in the XhoI and EcoRI sites of psiCHECK-1 to generate the luciferase reporter construct. The miR-34a recognition element is shown in red.

Rat Pdgfra 3'UTR

CTCGAGGTGTGCCAGGTCTGTGTCTAACCAGCTCCGGAACCGGTGGGGAAGATAA
AAAGGTTTCTGGATTTGGGGGCTTTAGGTGGGAGGCTATCACAAGCATCCAA**ACT**
GCCAAACTGGTTAGTGTGGGTTCATTGGCATTCTCTGCAATGTGTCTAATTGCTG
ACTCTGTATGAATGAAACATGGGGTATGATTAACTGCAATCGAATTC
