# Supplementary material for: Role of microRNAs in the age-associated decline of pancreatic beta cell function in rat islets
Source: Diabetologia. 2015 Oct 16;59(1):161–9. doi: 10.1007/s00125-015-3783-5 (PMC4670458; doi:10.1007/s00125-015-3783-5)
Supplement: Supplementary file 2 — (PDF 78 kb) [file 125_2015_3783_MOESM2_ESM.pdf]

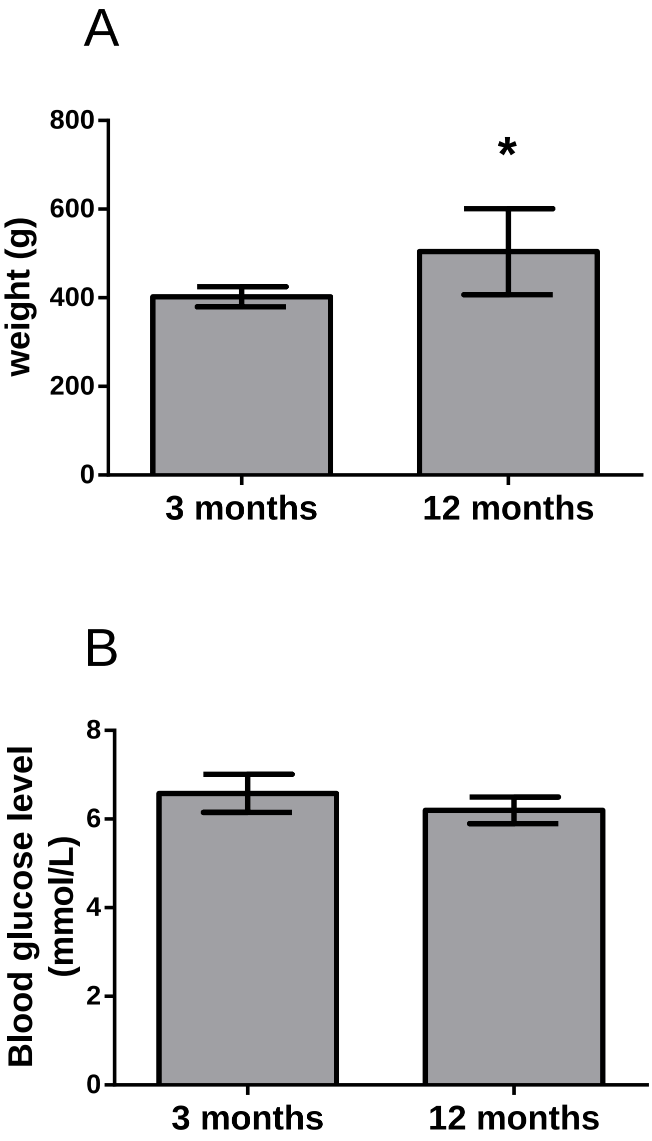

ESM FIG 1. Weight and glycemia of young and aged rats. 3 and 12 month-old rats were starved overnight. In the morning, they were weighted (A) and blood glucose levels were measured with a Accu-check® glucometer (B). The results correspond to the mean ± SD of six 12 month-old male rats and six 3 month-old control animals.

* Significantly different from control (p-value ≤ 0.05, Student T-test).
