# Supplementary material for: Role of microRNAs in the age-associated decline of pancreatic beta cell function in rat islets
Source: Diabetologia. 2015 Oct 16;59(1):161–9. doi: 10.1007/s00125-015-3783-5 (PMC4670458; doi:10.1007/s00125-015-3783-5)
Supplement: Supplementary file 3 — (PDF 83 kb) [file 125_2015_3783_MOESM3_ESM.pdf]

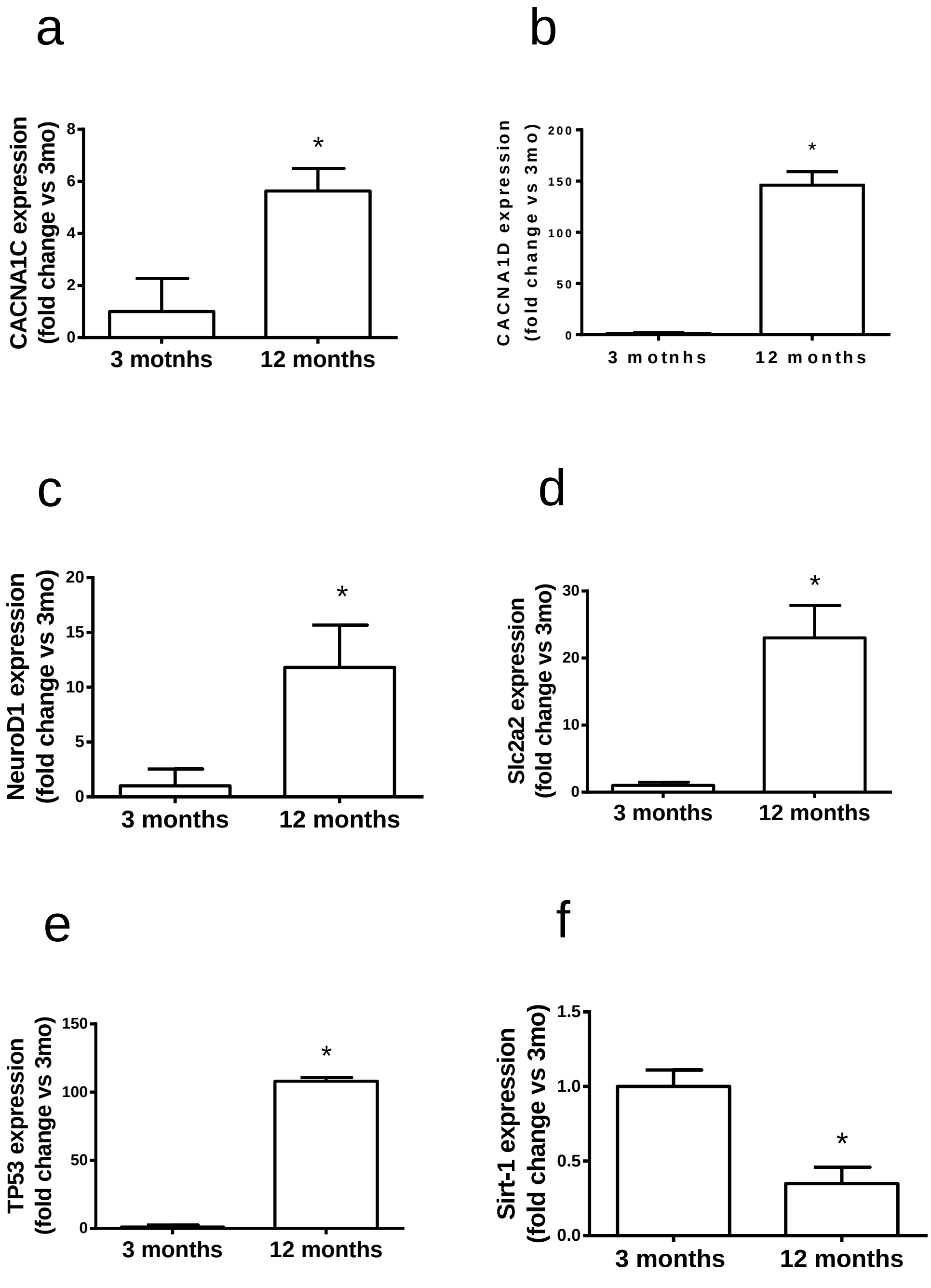

ESM FIG 2. Confirmation of the changes in the level of selected protein-coding genes by qRT-PCR. The expression of *Cacna1c* (a), *Cacna1d* (b), *Neurod1* (c), *Slc2a2* (d), *Tp53* (e) and *Sirt-1* (f) in pancreatic islets of 3 and 12 month old rats was assessed by qRT-PCR. Values are presented as fold changes versus the level measured in 3 month old rats. They represent the mean ± SD of four 12-month old animals versus four 3-month old animals . *Significantly different from 3-month old animals (control group) (p-value ≤ 0.05, Student T-test).
