# Supplementary material for: Role of microRNAs in the age-associated decline of pancreatic beta cell function in rat islets
Source: Diabetologia. 2015 Oct 16;59(1):161–9. doi: 10.1007/s00125-015-3783-5 (PMC4670458; doi:10.1007/s00125-015-3783-5)
Supplement: Supplementary file 4 — (PDF 11 kb) [file 125_2015_3783_MOESM4_ESM.pdf]

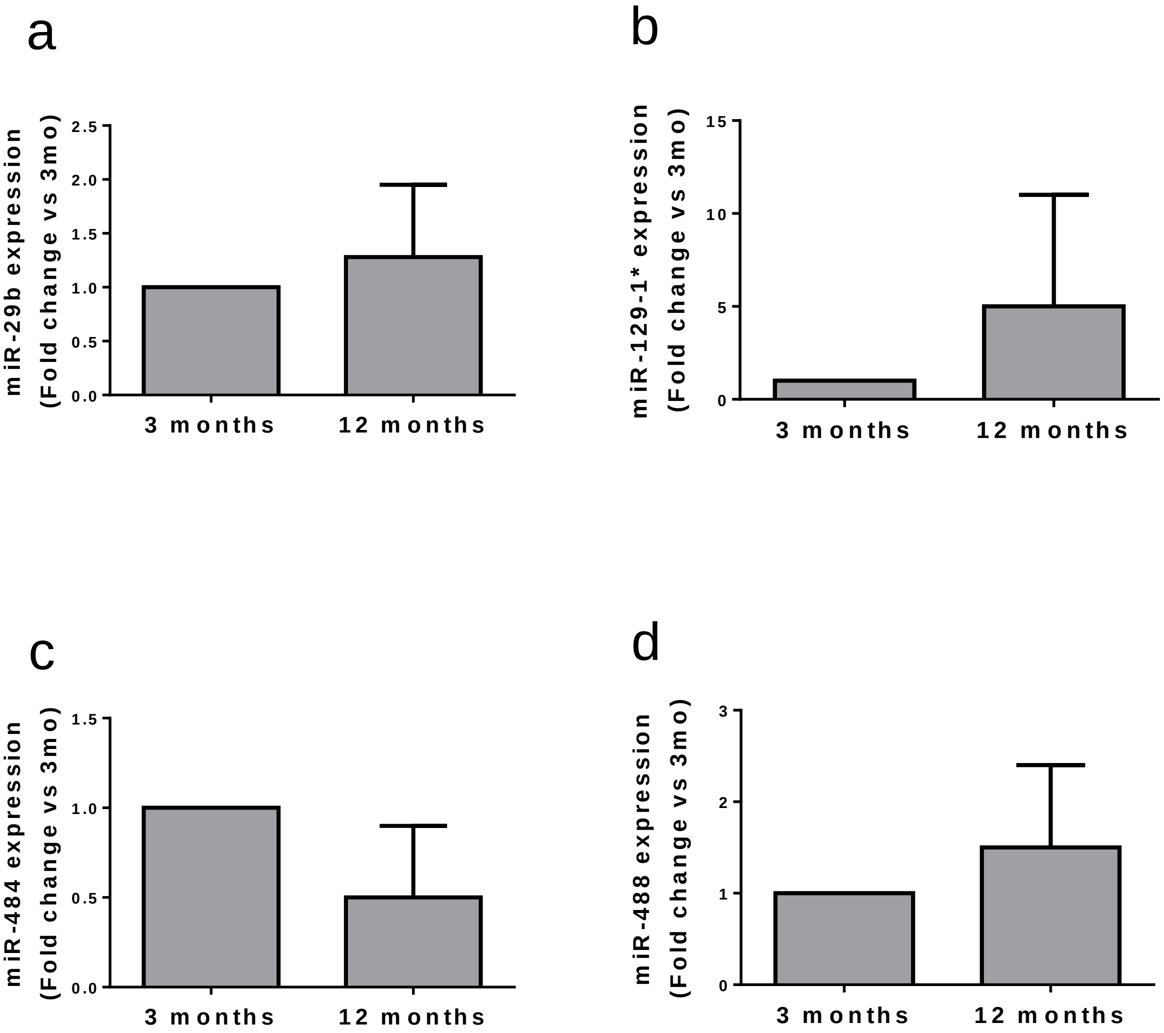

ESM FIG 3. MicroRNA expression in pancreatic islets of young and old rats. RNA was isolated from pancreatic islets of male Wistar rats aged 3 and 12 months. MicroRNA expression was assessed by qRT-PCR. Values are expressed as fold change *versus* the level measured in the islets of 3 month-old rats (n= 4).
