# Supplementary material for: Role of microRNAs in the age-associated decline of pancreatic beta cell function in rat islets
Source: Diabetologia. 2015 Oct 16;59(1):161–9. doi: 10.1007/s00125-015-3783-5 (PMC4670458; doi:10.1007/s00125-015-3783-5)
Supplement: Supplementary file 5 — (PDF 85 kb) [file 125_2015_3783_MOESM5_ESM.pdf]

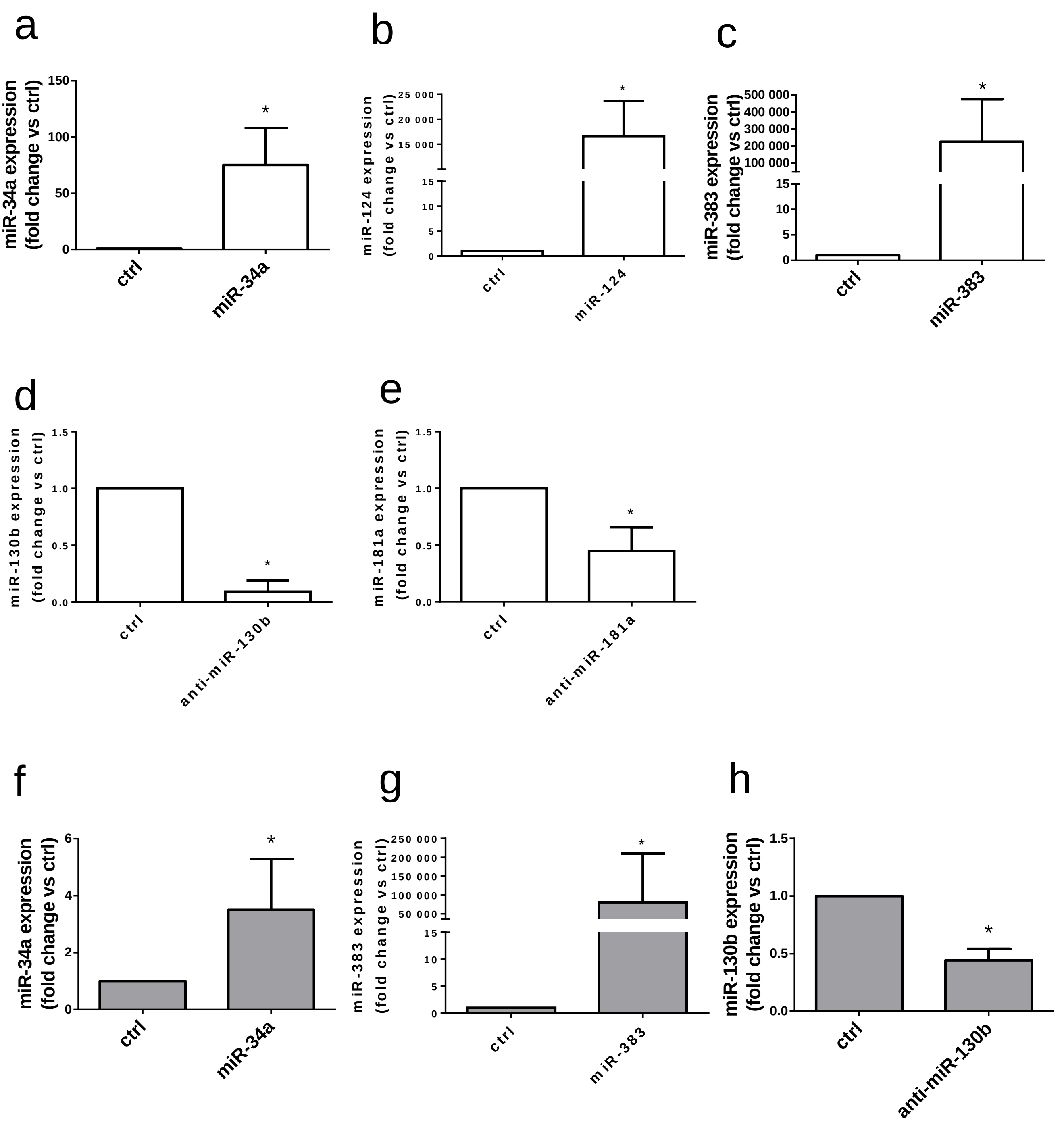

ESM FIG 4. Downregulation or overexpression of specific microRNAs in dispersed rat and human islet cells. Dispersed rat (a-e) or human (f-h) islet cells were transfected with the indicated miRNA mimics (a-c, g-h), anti-miRNAs (d,e,h) or their respective controls. miRNA overexpression or downregulation was measured by qRT-PCR. Results are expressed as fold change *versus* Ctrl and correspond to the mean ± SD of at least three independent experiments.

* Significantly different from control (p-value ≤ 0.05, Student T-test).
