# Supplementary material for: Role of microRNAs in the age-associated decline of pancreatic beta cell function in rat islets
Source: Diabetologia. 2015 Oct 16;59(1):161–9. doi: 10.1007/s00125-015-3783-5 (PMC4670458; doi:10.1007/s00125-015-3783-5)
Supplement: Supplementary file 6 — (PDF 80 kb) [file 125_2015_3783_MOESM6_ESM.pdf]

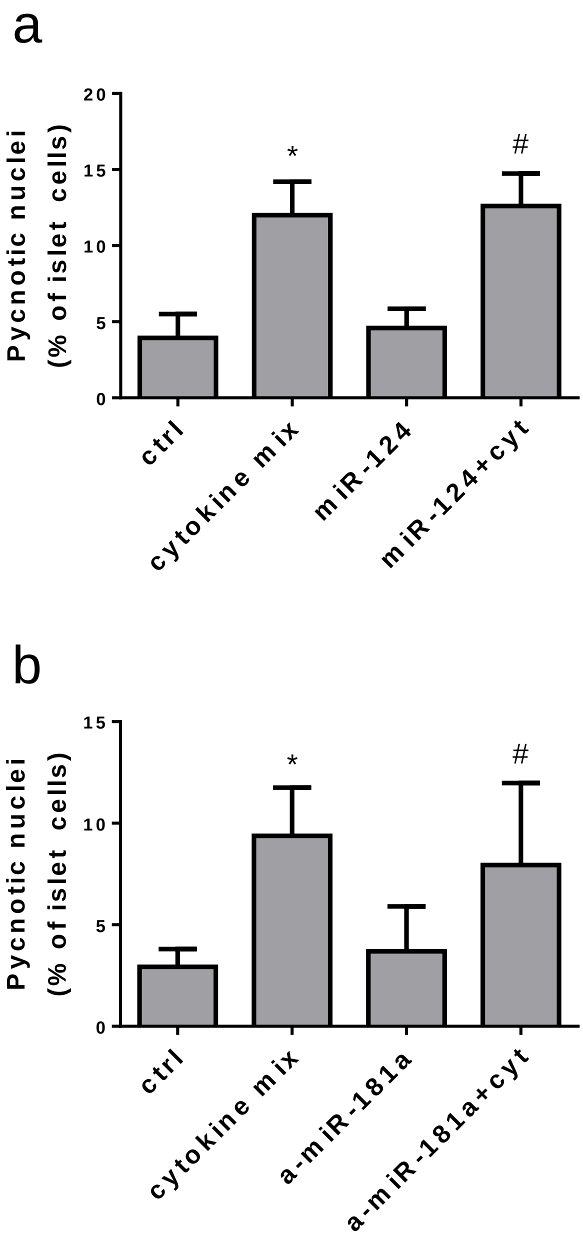

ESM FIG 5. Impact of changes in miRNA expression on islet-cell apoptosis. Dispersed islet cells were transfected with oligonucleotides leading to overexpression (A) or downregulation (B) of the indicated miRNAs. Cell death was assessed by scoring the cells displaying pycnotic nuclei upon Hoechst staining. Incubation during 24h with a mix of pro-inflammatory cytokines (10 ng/mL TNFα; 0.1 ng/mL IL-1β; 30ng/mL IFNγ) was used as a positive control for apoptosis. The results correspond to the mean ± SD of three to four independent experiments. * Significantly different from #significantly different from anti- or oligo-miR condition ($p < 0.05$ by ANOVA analysis, Dunnett's post-hoc test).
