# Supplementary material for: Role of microRNAs in the age-associated decline of pancreatic beta cell function in rat islets
Source: Diabetologia. 2015 Oct 16;59(1):161–9. doi: 10.1007/s00125-015-3783-5 (PMC4670458; doi:10.1007/s00125-015-3783-5)
Supplement: Supplementary file 7 — (PDF 2147 kb) [file 125_2015_3783_MOESM7_ESM.pdf]

**ESM Table 1**

## Analysis of the up-regulated mRNAs in the islets of 12 month-old rats

Two µg of RNA isolated from the islets of three 3 month-old and three 12 month-old rats were analyzed by microarray.
The signal intensities were quantile normalized and expressed on a Log2 scale.
Column B - E - Probe annotations (SeqID, Gene Symbol, description, chromosome)
Column F - P-value calculated from paired t-test
Column G - FCA absolute, an absolute fold change calculated between two groups
Column H,I - Normalized Intensity for each group (log2 transformed)
Column J - O - Normalized Intensity for each sample (log 2 transformed)
Fold Change cut off 2.0, p-value cut-off 0.5

| | | | | | | Normalized Intensity | | | | | |
|---|---|---|---|---|---|---|---|---|---|---|---|
| SeqID | Gene Symbol | Description | chromo-some | p-value | FC Absolute | 12 mo | 12 mo | 12 mo | 3 mo | 3 mo | 3 mo |
| NM_017020 | Il6ra | Rattus norvegicus interleukin 6 recepto | chr2 | 0.047679 | 11.902869 | 11.22947 | 12.0573 | 12.12635 | 9.179913 | 8.196045 | 7.317394 |
| NM_022685 | Rem2 | Rattus norvegicus rad and gem related | chr15 | 0.022098 | 2.8804815 | 10.99263 | 10.6077 | 11.37457 | 9.170772 | 9.536084 | 9.689097 |
| NM_031969 | Calm1 | Rattus norvegicus calmodulin 1 (Calm | chr6 | 0.034794 | 2.6234567 | 14.93681 | 14.8747 | 14.80736 | 13.24575 | 13.2511 | 13.94759 |
| NM_024359 | Hif1a | Rattus norvegicus hypoxia inducible fa | chr6 | 0.046032 | 3.2231255 | 11.77025 | 12.5562 | 11.78862 | 10.00422 | 10.25985 | 10.78559 |
| NM_001106581 | Pdk3 | pyruvate dehydrogenase kinase 3 | chrX | 0.001901 | 17.448597 | 7.180491 | 6.68609 | 7.596643 | 3.060453 | 2.870505 | 3.157151 |
| NM_001107465 | Chd1 | chromodomain helicase DNA binding | chr1 | 0.004625 | 6.147144 | 5.692766 | 6.4461 | 5.454534 | 2.817606 | 3.736898 | 3.179149 |
| NM_001012030 | Zfp281 | Rattus norvegicus zinc finger protein 2 | chr13 | 0.043007 | 2.1050541 | 10.35245 | 10.4139 | 10.33669 | 8.834476 | 9.6671 | 9.379844 |
| NM_031510 | Idh1 | Rattus norvegicus isocitrate dehydroge | chr9 | 0.008399 | 4.0477424 | 10.57618 | 10.2291 | 10.86028 | 8.363111 | 8.583876 | 8.667213 |
| NM_001110838 | Toag1 | tolerance-associated gene 1 | chr8 | 0.001683 | 19.18213 | 6.927327 | 6.85276 | 7.389266 | 2.817606 | 2.788281 | 2.77839 |
| NM_017198 | Pak1 | Rattus norvegicus p21 (CDKN1A)-act | chr1 | 0.011324 | 6.110785 | 7.582896 | 7.6287 | 8.039293 | 4.411103 | 5.287956 | 5.717759 |
| NM_145096 | Zdhhc2 | Rattus norvegicus zinc finger, DHHC | chr16 | 0.002084 | 8.128788 | 8.382111 | 8.94565 | 8.920061 | 5.094403 | 6.123974 | 5.960319 |
| NM_001108390 | Ndfip2 | Nedd4 family interacting protein 2 | chr15 | 0.025804 | 2.6310236 | 10.88382 | 10.9942 | 10.92048 | 9.038341 | 9.752488 | 9.820837 |
| NM_053773 | Tjp2 | Rattus norvegicus tight junction protei | chr1 | 0.016767 | 4.3541965 | 9.608555 | 9.10456 | 8.944793 | 7.524991 | 7.443657 | 6.32204 |
| NM_031743 | Slc24a2 | Rattus norvegicus solute carrier family | chr5 | 0.04682 | 14.2889385 | 8.355094 | 6.8078 | 8.94626 | 2.817606 | 4.052382 | 5.728688 |
| NM_001108552 | Trim2 | tripartite motif-containing 2 | chr2 | 0.014318 | 6.0540433 | 9.761775 | 10.1397 | 9.66022 | 6.571774 | 7.655322 | 7.540898 |
| NM_001012078 | Sema4a | Rattus norvegicus sema domain, immu | chr2 | 0.026363 | 3.1146963 | 11.47684 | 11.0392 | 11.44743 | 9.56892 | 9.943165 | 9.534121 |
| NM_001037347 | Sfrs15 | Rattus norvegicus splicing factor, argi | chr11 | 0.018908 | 19.370321 | 9.245078 | 9.80771 | 9.455865 | 4.085805 | 5.279558 | 6.315959 |
| NM_001106395 | Foxo3 | forkhead box O3 | chr20 | 0.044995 | 2.3483555 | 6.36811 | 5.81008 | 6.045876 | 4.896085 | 5.118303 | 4.514729 |

| | | | | | | | | | | | |
|---|---|---|---|---|---|---|---|---|---|---|---|
| NM_024398 | Aco2 | Rattus norvegicus aconitase 2, mitocho | chr7 | 0.015296 | 2.3120189 | 10.88617 | 10.769 | 11.1947 | 9.621634 | 9.845153 | 9.755635 |
| NM_001108620 | Nup205 | nucleoporin 205 | chr4 | 0.035305 | 2.0639873 | 11.48667 | 11.4364 | 11.36821 | 10.18454 | 10.7891 | 10.18137 |
| NM_080781 | Copb1 | Rattus norvegicus coatomer protein co | chr1 | 0.039759 | 2.0228078 | 14.60039 | 14.6671 | 14.55789 | 13.24479 | 13.60899 | 13.92247 |
| NM_207602 | St3gal6 | Rattus norvegicus ST3 beta-galactoside | chr11 | 0.001174 | 3.0352056 | 10.65101 | 10.7583 | 10.51693 | 9.155923 | 9.08058 | 8.884392 |
| NM_001107400 | Brunol4 | bruno-like 4, RNA binding protein" | chr18 | 0.026584 | 19.81529 | 8.102116 | 7.85467 | 7.508509 | 2.817606 | 4.943666 | 2.77839 |
| NM_001110782 | Syn1 | synapsin I isoform b | chrX | 0.00747 | 6.2750945 | 7.812203 | 8.26096 | 7.777534 | 4.701969 | 5.841997 | 5.357815 |
| NM_001107647 | Utp15 | UTP15, U3 small nucleolar ribonucleo | chr2 | 0.013471 | 2.7639298 | 8.454012 | 8.84707 | 8.691416 | 6.993045 | 7.675298 | 6.923988 |
| NM_001108239 | Gtf3c3 | general transcription factor IIIC, polyp | chr9 | 0.002917 | 11.917524 | 9.271097 | 9.41736 | 9.338932 | 5.534195 | 5.618829 | 6.149324 |
| NM_057205 | Ube1c | Rattus norvegicus ubiquitin-activating | chr4 | 0.025405 | 5.4789047 | 10.39618 | 10.5752 | 10.19289 | 7.256192 | 8.11227 | 8.434178 |
| NM_017298 | Cacna1d | Rattus norvegicus calcium channel, vo | chr16 | 0.033344 | 2.068794 | 10.02733 | 10.0892 | 10.40682 | 8.697262 | 9.418596 | 9.261078 |
| NM_001100722 | Lingo1 | leucine rich repeat and Ig domain conta | chr8 | 0.014929 | 2.7862523 | 10.25822 | 10.2662 | 10.80462 | 8.420609 | 8.910045 | 9.563419 |
| NM_001013143 | Terf2ip | Rattus norvegicus telomeric repeat bin | chr19 | 0.024611 | 2.0322044 | 12.0412 | 12.0365 | 11.89758 | 10.77606 | 11.32489 | 10.80514 |
| NM_001013076 | Dnajb4 | Rattus norvegicus DnaJ (Hsp40) homo | chr2 | 0.001752 | 3.0102928 | 9.826145 | 9.66484 | 10.02175 | 8.119774 | 8.189296 | 8.43396 |
| NM_001109608 | LOC69072 | hypothetical protein LOC690728 | chr4 | 0.016259 | 2.4517612 | 6.805542 | 6.40925 | 7.037242 | 5.706667 | 5.252867 | 5.411041 |
| NM_001025664 | Wsb1 | Rattus norvegicus WD repeat and SOC | chr10 | 0.026608 | 33.054752 | 11.29479 | 10.4845 | 10.42956 | 5.735397 | 7.079721 | 4.253364 |
| NM_001107213 | Uba6 | ubiquitin-like modifier activating enzy | chr14 | 0.013039 | 5.4294295 | 8.378452 | 7.96668 | 8.398852 | 5.887856 | 6.036383 | 5.497342 |
| NM_001012129 | Satb1 | Rattus norvegicus special AT-rich sequ | chr9 | 0.022256 | 2.2748795 | 11.11543 | 10.9539 | 11.37404 | 9.644484 | 10.10076 | 10.14077 |
| NM_001134414 | Tmtc4 | transmembrane and tetratricopeptide re | chr15 | 0.028191 | 4.3351793 | 9.162601 | 10.2527 | 10.45471 | 7.772354 | 7.788202 | 7.961219 |
| NM_001115027 | RGD15624 | hypothetical protein LOC317418 | chrX | 0.022663 | 5.9882727 | 11.53053 | 11.5884 | 11.59106 | 8.481719 | 9.79268 | 8.689201 |
| NM_001106005 | Gnpda2 | glucosamine-6-phosphate deaminase 2 | chr14 | 0.006881 | 5.8454175 | 10.7813 | 11.0636 | 10.80667 | 7.984308 | 8.938744 | 8.086558 |
| NM_001009675 | Tceal1 | Rattus norvegicus transcription elonga | chrX | 0.00357 | 4.6469703 | 10.87064 | 11.0898 | 10.99344 | 8.501822 | 9.138044 | 8.665154 |
| NM_031583 | Cspg6 | Rattus norvegicus chondroitin sulfate p | chr1 | 0.000119 | 2.327125 | 12.13201 | 12.257 | 12.16518 | 10.93483 | 11.01415 | 10.94959 |
| NM_001107712 | Cttnbp2nl | CTTNBP2 N-terminal like | chr2 | 0.001236 | 2.1693485 | 9.238039 | 9.16946 | 9.059008 | 8.086558 | 8.130604 | 7.897553 |
| NM_001037362 | MGC1250 | gluconokinase-like protein | chr17 | 0.042447 | 5.6088805 | 7.348463 | 6.70863 | 7.622128 | 4.961394 | 5.08364 | 4.171041 |
| NM_001108041 | Acot3 | acyl-CoA thioesterase 3 | chr6 | 0.017951 | 2.1297104 | 6.798192 | 6.28331 | 6.716238 | 5.847483 | 5.348814 | 5.329468 |
| NM_019365 | Rassf5 | Rattus norvegicus Ras association (Ral | chr13 | 0.002943 | 3.7190428 | 9.541564 | 9.64613 | 8.561038 | 7.824496 | 7.572189 | 6.667253 |
| NM_017326 | Calm2 | Rattus norvegicus calmodulin 2 (Calm | chr15 | 0.015206 | 2.20303 | 16.02861 | 16.2462 | 15.83753 | 15.00327 | 14.82418 | 14.86644 |
| NM_001135600 | Cyp4v3 | cytochrome P450, family 4, subfamily | chr16 | 0.007835 | 3.8489926 | 11.52926 | 11.6615 | 11.07662 | 9.61629 | 9.999954 | 8.817738 |
| NM_033485 | Pawr | Rattus norvegicus PRKC, apoptosis, W | chr7 | 0.019085 | 20.644209 | 9.393534 | 9.24025 | 8.610544 | 3.922654 | 4.964323 | 5.254356 |
| NM_001106635 | Arid1a | AT rich interactive domain 1A (SWI-li | chr5 | 0.000237 | 2.7336175 | 12.60827 | 12.74 | 12.08744 | 11.12416 | 11.27997 | 10.67913 |
| NM_199107 | Gyltl1b | Rattus norvegicus glycosyltransferase- | chr3 | 0.033231 | 2.5212486 | 8.399252 | 8.07773 | 9.056057 | 7.084751 | 7.165526 | 7.280344 |
| NM_019185 | Gata6 | Rattus norvegicus GATA binding prot | chr18 | 0.008382 | 14.309847 | 8.810637 | 8.35152 | 8.799225 | 4.937523 | 5.141569 | 4.365476 |
| NM_001007714 | Morf4l2 | Rattus norvegicus mortality factor 4 lik | chrX | 0.00675 | 9.609952 | 14.07985 | 14.1865 | 14.36724 | 10.61669 | 10.58724 | 11.63611 |

| | | | | | | | | | | | |
|---|---|---|---|---|---|---|---|---|---|---|---|
| NM_053718 | Mllt3 | myeloid/lymphoid or mixed-lineage le | chr5 | 0.039183 | 2.3859546 | 8.041844 | 8.11227 | 8.45624 | 6.884209 | 7.244484 | 6.71796 |
| NM_022856 | Nab1 | Rattus norvegicus Ngfi-A binding prot | chr9 | 0.004369 | 7.5511346 | 6.05719 | 6.56751 | 5.961792 | 2.817606 | 3.996757 | 3.022049 |
| NM_001134859 | Sec23ip | SEC23 interacting protein | chr1 | 0.025332 | 2.4242032 | 12.12293 | 12.0139 | 12.36592 | 10.65696 | 10.51086 | 11.50244 |
| NM_001024769 | Cdc73 | Rattus norvegicus cell division cycle 7 | chr13 | 0.036547 | 2.6935363 | 11.44239 | 12.2311 | 11.84646 | 10.53441 | 10.35932 | 10.33775 |
| NM_019275 | Smad4 | Rattus norvegicus MAD homolog 4 (D | chr18 | 0.02591 | 2.7883074 | 8.993723 | 8.93365 | 9.154389 | 7.063108 | 7.83564 | 7.744839 |
| NM_001014772 | Adam9 | Rattus norvegicus a disintegrin and me | chr16 | 0.0463 | 12.249964 | 7.036422 | 5.52837 | 8.862395 | 3.884074 | 3.019974 | 3.679025 |
| NM_001037188 | RGD13071 | hypothetical protein LOC302998 | chr10 | 0.028052 | 4.023906 | 5.849524 | 5.403 | 5.758212 | 4.140784 | 3.780132 | 3.064029 |
| NM_019178 | Tlr4 | Rattus norvegicus toll-like receptor 4 ( | chr5 | 0.03863 | 2.9964352 | 7.447341 | 7.4304 | 6.680788 | 5.583198 | 5.48854 | 5.737053 |
| NM_001106401 | Fam172a | hypothetical protein LOC294606 | chr2 | 0.010249 | 2.0193357 | 11.11975 | 11.1878 | 10.72 | 9.905207 | 10.31789 | 9.762861 |
| NM_001009626 | Apoh | Rattus norvegicus apolipoprotein H (A | chr10 | 0.006484 | 3.7258847 | 11.50755 | 11.4768 | 11.39886 | 9.309164 | 9.676118 | 9.705224 |
| NM_017258 | Btg1 | Rattus norvegicus B-cell translocation | chr7 | 0.031362 | 3.755796 | 14.76076 | 14.5836 | 14.48421 | 12.57201 | 13.36301 | 12.16618 |
| NM_017187 | Hmgb2 | high mobility group box 2 | chr16 | 0.004164 | 7.596887 | 10.40148 | 9.63854 | 9.494988 | 7.838357 | 6.627616 | 6.292809 |
| NM_001009714 | RGD1307 | Rattus norvegicus similar to hypothetic | chr9 | 0.026011 | 2.240546 | 6.271523 | 6.6832 | 6.287871 | 4.749575 | 5.815846 | 5.18562 |
| NM_001034021 | Tbc1d14 | TBC1 domain family, member 14 isof | chr14 | 0.007322 | 2.075241 | 12.14429 | 12.3628 | 11.9821 | 11.01069 | 11.49044 | 10.82827 |
| NM_183328 | Ssbp1 | Rattus norvegicus single-stranded DN | chr4 | 0.036432 | 2.321758 | 12.23596 | 11.9477 | 12.13556 | 10.54935 | 10.90485 | 11.21937 |
| NM_053363 | Clcn3 | Rattus norvegicus chloride channel 3 ( | chr16 | 0.027866 | 2.3717558 | 10.22807 | 10.6327 | 10.41133 | 8.687209 | 9.269326 | 9.577743 |
| NM_001033695 | L3mbtl2 | Rattus norvegicus l(3)mbt-like 2 (Dros | chr7 | 0.026559 | 2.1838055 | 11.90579 | 11.8685 | 11.74265 | 10.73473 | 11.08612 | 10.3156 |
| NM_173153 | Gimap4 | Rattus norvegicus GTPase, IMAP fam | chr4 | 0.035174 | 3.8921413 | 8.012702 | 6.83422 | 7.467863 | 6.391192 | 5.288807 | 4.753098 |
| NM_013011 | Ywhaz | Rattus norvegicus tyrosine 3-monooxy | chr7 | 0.008389 | 9.355037 | 13.54317 | 13.182 | 12.98919 | 10.74225 | 10.1042 | 9.190692 |
| NM_001013130 | Spnb2 | Rattus norvegicus spectrin beta 2 (Spn | chr14 | 0.008196 | 10.755171 | 10.44727 | 9.48578 | 11.7664 | 6.910295 | 6.646076 | 7.862207 |
| NM_012992 | Npm1 | Rattus norvegicus nucleophosmin 1 (N | chr10 | 0.042261 | 2.896915 | 14.39998 | 13.944 | 14.40203 | 12.84055 | 12.98595 | 12.31594 |
| NM_001107678 | Dhx36 | DEAH (Asp-Glu-Ala-His) box polype | chr2 | 0.021008 | 4.7017884 | 11.42222 | 11.3965 | 11.14451 | 8.847573 | 8.847067 | 9.56892 |
| NM_001108707 | Twistnb | TWIST neighbor | chr6 | 0.009297 | 2.4929476 | 6.236426 | 5.84255 | 6.681898 | 4.983605 | 4.706543 | 5.117172 |
| NM_001034855 | Gpr153 | Rattus norvegicus G protein-coupled r | chr5 | 0.043116 | 2.8806548 | 6.876765 | 6.86419 | 6.535305 | 4.930239 | 5.983357 | 4.783471 |
| NM_173099 | Pcdh3 | Rattus norvegicus protocadherin 3 (Pc | chr18 | 0.031164 | 6.600426 | 6.145409 | 6.84939 | 5.923888 | 3.200852 | 3.407279 | 4.142882 |
| NM_001005873 | Dhx40 | Rattus norvegicus DEAH (Asp-Glu-Al | chr10 | 0.001836 | 2.6803324 | 10.96149 | 11.5146 | 11.39835 | 9.533405 | 9.989442 | 10.08436 |
| NM_001100472 | Ccnc | cyclin C | chr5 | 0.025458 | 2.663694 | 11.54229 | 11.8782 | 11.50469 | 9.871765 | 10.26315 | 10.55001 |
| NM_198736 | Kctd13 | Rattus norvegicus potassium channel t | chr1 | 0.04335 | 2.656035 | 11.2141 | 11.4291 | 11.23981 | 10.23679 | 10.17286 | 9.245547 |
| NM_001012744 | Enpp5 | Rattus norvegicus ectonucleotide pyro | chr9 | 0.041831 | 2.918415 | 8.434178 | 7.86599 | 9.553082 | 6.74399 | 6.944446 | 7.529253 |
| NM_001029925 | LOC49947 | Rattus norvegicus similar to osteopetro | chr20 | 0.032656 | 4.4691954 | 7.750963 | 7.45692 | 8.225211 | 5.313462 | 6.085827 | 5.553756 |
| NM_019218 | Neurod1 | Rattus norvegicus neurogenic different | chr3 | 0.001415 | 4.605801 | 13.79482 | 14.262 | 14.14675 | 11.45081 | 12.05242 | 12.08998 |
| NM_001109388 | LOC68003 | hypothetical protein LOC680039 | chr14 | 0.04964 | 4.0762353 | 8.818294 | 9.29004 | 8.981901 | 6.067896 | 7.10614 | 7.834485 |
| NM_001135013 | Ankrd42 | ankyrin repeat domain 42 | chr1 | 0.033522 | 2.1653593 | 8.698883 | 9.15592 | 8.478096 | 7.165526 | 8.250258 | 7.573299 |

| | | | | | | | | | | | |
|---|---|---|---|---|---|---|---|---|---|---|---|
| NM_017229 | Pde3b | Rattus norvegicus phosphodiesterase 3 | chr1 | 0.013883 | 3.8918211 | 11.22852 | 11.6186 | 11.16733 | 8.95446 | 9.515474 | 9.663204 |
| NM_001017960 | Thra | Rattus norvegicus thyroid hormone rec | chr10 | 0.047442 | 2.5631437 | 7.051135 | 7.3594 | 8.411622 | 5.285947 | 6.602564 | 6.859896 |
| NM_001127492 | Sphkap | SPHK1 interactor, AKAP domain cont | chr9 | 0.029298 | 8.303061 | 10.09632 | 9.91887 | 11.39605 | 8.053043 | 6.662434 | 7.534836 |
| NM_001130538 | Slc30a8 | solute carrier family 30 (zinc transport | chr7 | 0.023813 | 13.176388 | 13.83067 | 13.284 | 14.89712 | 10.62963 | 10.2119 | 10.01057 |
| NM_001004233 | MGC9494 | Rattus norvegicus similar to Mkrn1 pro | chr4 | 0.044234 | 2.129443 | 9.753067 | 10.0807 | 10.06741 | 8.507475 | 9.456225 | 8.666061 |
| NM_031333 | Cdh2 | Rattus norvegicus cadherin 2 (Cdh2), r | chr18 | 0.04637 | 10.0071745 | 7.841231 | 8.32162 | 8.201783 | 3.252816 | 4.961394 | 6.181541 |
| NM_001113365 | Tbc1d14 | TBC1 domain family, member 14 isof | chr14 | 0.003563 | 2.205176 | 12.14962 | 12.3684 | 11.90786 | 10.88617 | 11.34096 | 10.77606 |
| NM_001007643 | Tmx2 | Rattus norvegicus thioredoxin-related t | chr3 | 0.036545 | 5.2056584 | 9.91197 | 9.00806 | 10.21348 | 7.409835 | 7.492539 | 7.090886 |
| NM_031122 | St13 | Rattus norvegicus suppression of tumo | chr7 | 0.01011 | 3.3784778 | 7.639179 | 7.82338 | 8.566155 | 5.966939 | 6.324428 | 6.468224 |
| NM_057107 | Acsl3 | Rattus norvegicus acyl-CoA synthetase | chr9 | 0.00061 | 2.0258148 | 11.27648 | 11.2387 | 11.05664 | 10.23895 | 10.18933 | 10.088 |
| NM_001024269 | Luc7l | Rattus norvegicus LUC7-like (S. cerev | chr10 | 0.018441 | 6.164453 | 8.096165 | 7.57085 | 7.871833 | 4.759005 | 5.404645 | 5.503281 |
| NM_001014263 | Sypl | Rattus norvegicus synaptophysin-like p | chr6 | 0.036895 | 3.029589 | 11.26136 | 11.3159 | 11.64981 | 9.617693 | 10.28496 | 9.527043 |
| NM_012576 | Nr3c1 | Rattus norvegicus nuclear receptor sub | chr18 | 0.005603 | 2.7724783 | 13.05219 | 13.298 | 13.15055 | 11.44743 | 11.74091 | 11.89882 |
| NM_001108927 | Osbp | oxysterol binding protein | chr1 | 0.030178 | 5.7915688 | 10.44027 | 9.7504 | 10.75194 | 7.90418 | 7.997729 | 7.438848 |
| NM_017118 | Capns1 | Rattus norvegicus calpain, small subun | chr1 | 0.049707 | 2.8631322 | 13.32045 | 12.2456 | 13.03659 | 11.61414 | 11.40899 | 11.02672 |
| NM_057139 | Hnrpu | Rattus norvegicus heterogeneous nucle | chr13 | 0.013466 | 2.0676126 | 13.35177 | 13.3606 | 13.07345 | 12.35674 | 12.49397 | 11.79123 |
| NM_022539 | Metap2 | Rattus norvegicus methionine aminope | chr7 | 9.13E-06 | 2.176119 | 13.04095 | 13.0024 | 12.94651 | 11.91274 | 11.88207 | 11.82977 |
| NM_001006996 | Ddx18 | Rattus norvegicus DEAD (Asp-Glu-Al | chr13 | 0.042296 | 4.744321 | 9.079847 | 8.71298 | 9.032461 | 6.863945 | 7.277795 | 5.944941 |
| NM_178106 | Entpd3 | Rattus norvegicus ectonucleoside triph | chr8 | 0.020009 | 2.0712802 | 14.85737 | 14.8556 | 14.90477 | 13.95975 | 13.95386 | 13.55252 |
| NM_001106217 | Lrp11 | low density lipoprotein receptor-relate | chr1 | 0.023672 | 3.5941572 | 15.12459 | 15.4528 | 15.20135 | 12.85298 | 14.15881 | 13.22999 |
| NM_031350 | Pex3 | Rattus norvegicus peroxisomal biogen | chr1 | 0.032195 | 3.6687763 | 9.601163 | 9.91931 | 9.303808 | 7.040376 | 8.320596 | 7.837413 |
| NM_001033696 | Hnrpdl | Rattus norvegicus heterogeneous nucle | chr14 | 0.027865 | 2.9899929 | 12.01166 | 11.3269 | 11.87461 | 10.12335 | 10.28371 | 10.06573 |
| NM_001106963 | Eif1ay | eukaryotic translation initiation factor | chrX | 0.047656 | 2.2401557 | 11.82321 | 11.6231 | 11.66268 | 10.62408 | 10.93263 | 10.06144 |
| NM_017094 | Ghr | Rattus norvegicus growth hormone rec | chr2 | 0.019106 | 98.48134 | 9.417356 | 8.57091 | 11.01955 | 2.817606 | 3.54648 | 2.77839 |
| NM_001007014 | LOC47415 | Rattus norvegicus zinc responsive prot | chr1 | 0.001027 | 2.0652347 | 10.34857 | 10.7411 | 10.3672 | 9.277342 | 9.653342 | 9.387312 |
| NM_001108792 | RGD1310 | hypothetical protein LOC363212 | chr9 | 0.040267 | 5.7360144 | 8.39685 | 8.11948 | 8.222951 | 5.163824 | 6.615365 | 5.399941 |
| NM_001024800 | Txndc1 | Rattus norvegicus thioredoxin domain | chr6 | 0.001278 | 5.363224 | 12.30638 | 12.2273 | 12.40226 | 9.716491 | 9.928602 | 10.02152 |
| NM_001004262 | Cog6 | Rattus norvegicus component of oligor | chr2 | 0.045277 | 5.9084077 | 10.27151 | 9.88687 | 10.63325 | 8.200704 | 7.958495 | 6.944124 |
| NM_001109605 | LOC69061 | hypothetical protein LOC690617 | chr7 | 0.01865 | 6.1450615 | 5.573004 | 6.04899 | 5.724621 | 3.639251 | 2.881047 | 2.968034 |
| NM_001106478 | Hltf | helicase-like transcription factor | chr2 | 0.046056 | 4.072552 | 11.0988 | 11.1785 | 11.04695 | 8.999099 | 9.967136 | 8.280245 |
| NM_001135008 | Pabpn1 | poly(A) binding protein, nuclear 1" | chr15 | 0.017034 | 5.7451386 | 8.666482 | 8.89017 | 9.632247 | 6.189416 | 6.921467 | 6.510991 |
| NM_022397 | Hnrpf | Rattus norvegicus heterogeneous nucle | chr4 | 0.007702 | 3.0927231 | 13.50768 | 13.3404 | 13.18753 | 11.59738 | 11.80105 | 11.75056 |
| NM_133526 | Tspan8 | Rattus norvegicus tetraspanin 8 (Tspan | chr7 | 0.044804 | 2.3727877 | 12.33721 | 10.7722 | 11.34096 | 10.9084 | 10.06266 | 9.739526 |

| | | | | | | | | | | | |
|---|---|---|---|---|---|---|---|---|---|---|---|
| NM_001106413 | Card6 | caspase recruitment domain family, me | chr2 | 0.026649 | 4.3283987 | 7.119383 | 7.31036 | 7.211463 | 4.52577 | 4.989882 | 5.784054 |
| NM_053883 | Dusp6 | Rattus norvegicus dual specificity phos | chr7 | 0.030549 | 5.2750444 | 6.18606 | 5.30281 | 6.305006 | 3.134681 | 3.713162 | 3.748488 |
| NM_001108081 | Actr6 | ARP6 actin-related protein 6 homolog | chr7 | 0.041577 | 2.6895492 | 10.8339 | 11.1146 | 10.99297 | 9.630854 | 10.05794 | 8.970582 |
| NM_001108530 | Ube2d1 | ubiquitin-conjugating enzyme E2D 1, U | chr20 | 0.029393 | 15.844743 | 9.449048 | 10.0394 | 9.212891 | 4.167052 | 6.248285 | 6.328181 |
| NM_181631 | Fbxo11 | Rattus norvegicus F-box only protein 1 | chr6 | 0.014156 | 2.2846584 | 12.80217 | 13.234 | 12.72548 | 11.50529 | 11.86344 | 11.81702 |
| NM_012870 | Tnfrsf11b | Rattus norvegicus tumor necrosis facto | chr7 | 0.01722 | 7.780062 | 12.65216 | 12.9996 | 12.75686 | 8.936412 | 10.60729 | 9.98557 |
| NM_001011988 | Cnot2 | CCR4-NOT transcription complex, sul | chr7 | 0.018307 | 8.402032 | 9.765544 | 9.47815 | 10.38519 | 6.478012 | 7.220925 | 6.717732 |
| NM_019356 | Eif2s1 | Rattus norvegicus eukaryotic translatio | chr6 | 0.028758 | 11.132481 | 9.598604 | 9.47772 | 10.29837 | 7.148978 | 6.033777 | 5.761836 |
| NM_001100529 | Txndc13 | thioredoxin domain containing 13 | chr3 | 0.007241 | 2.7270136 | 13.12684 | 13.4649 | 13.04051 | 11.56447 | 11.88518 | 11.84065 |
| NM_001033909 | Elf2 | Rattus norvegicus E74-like factor 2 (E | chr2 | 0.00776 | 3.4007366 | 10.58671 | 10.8564 | 10.2208 | 8.839663 | 9.351631 | 8.17503 |
| NM_053352 | Cmkor1 | Rattus norvegicus chemokine orphan r | chr9 | 0.022399 | 6.044332 | 9.254816 | 8.23384 | 7.75493 | 5.870347 | 5.992278 | 5.594211 |
| NM_001105828 | Supt4h1 | suppressor of Ty 4 homolog 1 | chr10 | 0.042023 | 3.396744 | 10.45731 | 10.6027 | 10.10211 | 8.141422 | 8.678252 | 9.049993 |
| NM_001109023 | Pnn | pinin, desmosome associated protein" | chr6 | 0.003495 | 9.403201 | 10.58124 | 10.9719 | 10.80538 | 7.272319 | 7.451276 | 7.935471 |
| NM_001106752 | Ttc8 | tetratricopeptide repeat domain 8 | chr6 | 0.014847 | 11.960775 | 6.467107 | 5.57212 | 7.085772 | 2.817606 | 2.788281 | 2.77839 |
| NM_031082 | Pggt1b | Rattus norvegicus protein geranylgerar | chr18 | 0.027032 | 2.4332886 | 9.550145 | 9.8427 | 9.442774 | 8.047593 | 8.348827 | 8.590474 |
| NM_053886 | Lman1 | Rattus norvegicus lectin, mannose-binc | chr18 | 0.019326 | 6.6015706 | 9.295324 | 9.0742 | 10.15653 | 6.190547 | 7.119619 | 7.047468 |
| NM_001012187 | Klhl7 | Rattus norvegicus kelch-like 7 (Drosop | chr4 | 0.042724 | 4.0185943 | 13.65011 | 14.1619 | 13.46331 | 11.11412 | 11.83591 | 12.30527 |
| NM_001014793 | Kpna4 | Rattus norvegicus karyopherin (import | chr2 | 0.023591 | 3.245464 | 5.139023 | 6.3511 | 6.290453 | 3.921326 | 4.216644 | 4.547328 |
| NM_001025677 | RGD13087 | Rattus norvegicus similar to Zinc finge | chr1 | 0.030435 | 2.350996 | 7.098609 | 7.45034 | 7.143664 | 5.460831 | 6.570176 | 5.961792 |
| NM_199381 | NAPE-PLI | Rattus norvegicus N-acyl-phosphatidyl | chr4 | 0.041134 | 2.3019447 | 5.69526 | 6.62895 | 6.209895 | 3.98915 | 5.661934 | 5.274461 |
| NM_020101 | Centa2 | Rattus norvegicus centaurin, alpha 2 (C | chr10 | 0.035383 | 3.5670881 | 7.779608 | 8.27388 | 8.656558 | 6.151907 | 6.92304 | 6.130858 |
| NM_001108572 | Ehmt1 | euchromatic histone-lysine N-methyltr: | chr3 | 0.014327 | 2.2232392 | 10.4189 | 10.7355 | 10.23073 | 9.33309 | 9.783977 | 8.810106 |
| NM_001106842 | Senp6 | SUMO/sentrin specific peptidase 6 | chr8 | 0.022787 | 2.1650908 | 9.83047 | 9.97096 | 10.14744 | 8.834894 | 9.075134 | 8.695558 |
| NM_053720 | Aatf | Rattus norvegicus apoptosis antagonizi | chr10 | 0.030364 | 3.5262628 | 9.978446 | 9.91064 | 10.52755 | 7.930221 | 8.732617 | 8.299373 |
| NM_017359 | Rab10 | Rattus norvegicus RAB10, member RA | chr6_random | 0.009477 | 2.2678106 | 13.17831 | 13.3503 | 12.9672 | 11.81702 | 12.3853 | 11.74962 |
| NM_001007608 | Skp1a | Rattus norvegicus S-phase kinase-asso | chr10 | 0.036408 | 2.1020658 | 14.0015 | 14.0122 | 14.14009 | 13.10657 | 13.18243 | 12.64937 |
| NM_001014779 | Pcdhb8 | protocadherin beta 8 | chr18 | 0.04964 | 5.487075 | 5.071578 | 5.23824 | 6.941399 | 2.817606 | 3.650332 | 3.415162 |
| NM_013135 | Rasa1 | Rattus norvegicus RAS p21 protein act | chr2 | 0.001214 | 6.869526 | 9.797706 | 10.1392 | 10.07804 | 6.932507 | 7.250502 | 7.491254 |
| NM_001037316 | Nat1 | N-acetyltransferase 1 | chr16 | 0.025324 | 4.1989117 | 9.111841 | 9.10837 | 9.011518 | 6.451318 | 7.610758 | 6.959601 |
| NM_001077429 | Lnp | lunapark | chr3 | 0.013048 | 2.0779371 | 7.829025 | 8.04582 | 8.355094 | 7.002469 | 6.948883 | 7.11313 |
| NM_001107268 | Mtmr6 | myotubularin related protein 6 | chr15 | 0.02273 | 3.8750887 | 11.02088 | 11.1899 | 11.62411 | 9.068066 | 9.754137 | 9.149965 |
| NM_001009413 | RGD13064 | Rattus norvegicus similar to EST AA7 | chr4 | 0.037559 | 3.1397693 | 12.39887 | 12.4935 | 12.77238 | 10.84777 | 11.35676 | 10.50822 |
| NM_053306 | Pak2 | Rattus norvegicus p21 (CDKN1A)-act | chr11 | 0.017802 | 7.4891148 | 6.696454 | 6.89389 | 7.517683 | 3.098304 | 4.655787 | 4.639552 |

| | | | | | | | | | | | | |
|---|---|---|---|---|---|---|---|---|---|---|---|---|
| NM_013146 | Cald1 | Rattus norvegicus caldesmon 1 (Cald1) | chr4 | 0.045126 | 7.4271684 | 10.58724 | 9.31749 | 10.05658 | 6.42964 | 6.935749 | 7.917479 |
| NM_012964 | Hmmr | Rattus norvegicus hyaluronan mediatec | chr10 | 0.024381 | 7.0650063 | 6.525191 | 6.25337 | 6.086936 | 2.817606 | 3.757795 | 3.828025 |
| NM_001014028 | RGD13052 | Rattus norvegicus similar to Cgi67 seri | chr1 | 0.009141 | 3.0329933 | 10.57833 | 10.809 | 10.67651 | 8.703139 | 9.224 | 9.334446 |
| NM_001109444 | LOC68157 | hypothetical protein LOC681578 | chr2 | 0.039207 | 2.0448916 | 12.38933 | 12.5993 | 12.11701 | 10.99081 | 11.57079 | 11.44799 |
| NM_198766 | RGD73510 | Rattus norvegicus similar to RIKEN cI | chr1 | 0.041809 | 2.0953166 | 9.381577 | 9.57464 | 9.955333 | 7.927256 | 8.900829 | 8.881965 |
| NM_001108479 | Vstm2b | V-set and transmembrane domain cont | chr1 | 0.010493 | 2.1804929 | 7.132378 | 8.1109 | 7.475488 | 5.911548 | 6.851266 | 6.581996 |
| NM_012761 | Sp4 | Rattus norvegicus Sp4 transcription fac | chr6 | 0.003133 | 11.670734 | 6.142535 | 6.73013 | 6.346369 | 2.817606 | 2.788281 | 2.978674 |
| NM_031345 | Tsc22d3 | Rattus norvegicus TSC22 domain fami | chrX | 0.023853 | 11.842899 | 10.44653 | 9.86757 | 10.23271 | 5.860373 | 6.408051 | 7.580535 |
| NM_013040 | Abcc9 | Rattus norvegicus ATP-binding cassett | chr4_random | 0.031667 | 7.10806 | 9.695834 | 9.62303 | 9.310858 | 5.841997 | 7.411932 | 6.887429 |
| NM_001025702 | Phf20l1 | PHD finger protein 20-like 1 | chr7 | 0.026703 | 6.8673034 | 6.774415 | 7.2443 | 6.728283 | 3.096655 | 4.713854 | 4.597255 |
| NM_001009542 | MGC72992 | Rattus norvegicus similar to programm | chr2 | 0.005421 | 4.824751 | 11.22587 | 11.2285 | 11.39942 | 8.739399 | 8.843557 | 9.459494 |
| NM_001109220 | Pex1 | peroxisome biogenesis factor 1 | chr4 | 0.024916 | 2.5120082 | 10.73363 | 11.403 | 10.64938 | 8.999519 | 10.15887 | 9.641118 |
| NM_001037179 | Lrrc8c | Rattus norvegicus leucine rich repeat c | chr14 | 0.037416 | 6.008501 | 8.935688 | 8.78212 | 8.412785 | 5.384218 | 6.364352 | 6.621007 |
| NM_001108431 | Dtwd2 | DTW domain containing 2 | chr18 | 0.015931 | 4.664092 | 8.78407 | 9.22244 | 8.66199 | 6.002236 | 7.201953 | 6.799521 |
| NM_001014000 | RGD13053 | Rattus norvegicus similar to RIKEN cI | chr16 | 0.003462 | 7.9830585 | 10.20122 | 10.4665 | 10.18068 | 6.910995 | 7.78727 | 7.159315 |
| NM_001106154 | Tmco6 | transmembrane and coiled-coil domain | chr18 | 0.028999 | 4.328609 | 9.498068 | 10.0004 | 9.242783 | 6.669629 | 8.090727 | 7.639179 |
| NM_024404 | Hnrpd | Rattus norvegicus heterogeneous nucle | chr14 | 0.00763 | 3.637284 | 13.18753 | 13.2186 | 12.95646 | 10.99765 | 11.50707 | 11.26926 |
| NM_001107953 | Zcchc11 | zinc finger, CCHC domain containing | chr5 | 0.015797 | 6.104517 | 9.864268 | 10.0367 | 9.846365 | 6.738905 | 8.046978 | 7.131851 |
| NM_031672 | Slc15a2 | Rattus norvegicus solute carrier family | chr11 | 0.015145 | 2.20518 | 7.80094 | 8.6892 | 7.90137 | 6.677249 | 7.294162 | 6.997413 |
| NM_001109005 | Rab23 | RAB23, member RAS oncogene family | chr9 | 0.027926 | 2.1881218 | 5.787474 | 6.24218 | 5.95922 | 4.498089 | 5.496372 | 4.605331 |
| NM_001039037 | Zdhhc13 | Rattus norvegicus zinc finger, DHHC c | chr1 | 0.026015 | 6.304578 | 11.22262 | 11.4347 | 11.31492 | 8.212263 | 9.647361 | 8.143392 |
| NM_001105960 | RGD13086 | hypothetical protein LOC289088 | chr13 | 0.047628 | 2.130837 | 10.15696 | 10.4258 | 9.783421 | 8.572323 | 9.553871 | 8.965736 |
| NM_001145840 | Ganc | glucosidase, alpha neutral C" | chr3 | 0.022709 | 8.385126 | 9.161615 | 9.24704 | 8.734746 | 5.269739 | 6.198278 | 6.471893 |
| NM_031238 | Sh3gl3 | Rattus norvegicus SH3-domain GRB2- | chr1 | 0.042422 | 3.2456787 | 8.991162 | 9.51236 | 8.861537 | 6.671093 | 7.804956 | 7.793447 |
| NM_199494 | Fpgt | Rattus norvegicus fucose-1-phosphate | chr2 | 0.047425 | 3.4957235 | 9.183058 | 9.33632 | 9.440398 | 6.66829 | 8.234393 | 7.640317 |
| NM_001031822 | Copg | Rattus norvegicus coatomer protein co | chr4 | 0.046595 | 3.7549767 | 12.8945 | 12.5956 | 13.19107 | 11.04511 | 11.39511 | 10.51459 |
| NM_133381 | Crebbp | Rattus norvegicus CREB binding prote | chr10 | 0.013108 | 2.167024 | 9.764864 | 10.4647 | 9.7199 | 8.452357 | 9.591857 | 8.558066 |
| NM_001113752 | Tmem189 | transmembrane protein 189 | chr3 | 0.03719 | 4.6968694 | 9.196323 | 9.31268 | 9.628202 | 6.153894 | 7.795093 | 7.493119 |
| NM_001139493 | Qser1 | glutamine and serine rich 1 | chr3 | 0.030189 | 5.070275 | 6.644351 | 6.99886 | 6.767088 | 3.497058 | 5.24362 | 4.643424 |
| NM_001139484 | Znf644 | zinc finger protein 644 | chr14 | 0.000136 | 17.682863 | 8.567986 | 8.84489 | 8.331184 | 4.344625 | 4.788328 | 4.178267 |
| NM_001014205 | Tmem123 | Rattus norvegicus transmembrane prot | chr8 | 0.047458 | 2.6298308 | 14.68463 | 14.5464 | 14.63068 | 13.20851 | 13.73347 | 12.73485 |
| NM_001009180 | Capza2 | Rattus norvegicus capping protein (act | chr4 | 0.005375 | 3.5640016 | 13.29933 | 13.4849 | 12.99863 | 11.71897 | 11.44362 | 11.11975 |
| NM_017096 | Crp | Rattus norvegicus C-reactive protein, p | chr13 | 0.004856 | 14.137718 | 8.928203 | 9.57892 | 8.790823 | 4.718851 | 6.269903 | 4.844755 |

| | | | | | | | | | | | |
|---|---|---|---|---|---|---|---|---|---|---|---|
| NM_001134708 | Odf2l | outer dense fiber of sperm tails 2-like | chr2 | 0.012801 | 10.448503 | 7.459498 | 7.21364 | 7.186761 | 3.493676 | 4.56129 | 3.649259 |
| NM_001109968 | Gls | glutaminase isoform b | chr9 | 9.67E-05 | 3.9462936 | 12.76108 | 12.6235 | 12.40787 | 10.74465 | 10.64793 | 10.45836 |
| NM_001009349 | Mthfs | Rattus norvegicus 5,10-methenyltetrah | chr8 | 0.012671 | 2.7538292 | 11.94505 | 11.9634 | 11.75796 | 10.27299 | 10.82969 | 10.17937 |
| NM_001013047 | Mtm1 | Rattus norvegicus X-linked myotubula | chr7 | 0.049263 | 2.1490154 | 10.23271 | 10.5169 | 10.59094 | 8.892183 | 9.921788 | 9.215589 |
| NM_001107879 | Rybp | RING1 and YY1 binding protein | chr4 | 0.010871 | 2.3765283 | 12.25373 | 12.7293 | 12.13309 | 10.75475 | 11.53632 | 11.07846 |
| NM_001013933 | Ube2a | Rattus norvegicus ubiquitin-conjugatin | chrX | 0.034759 | 2.5543053 | 9.480625 | 9.82352 | 9.263508 | 7.639179 | 8.864248 | 8.005434 |
| NM_053769 | Dusp1 | Rattus norvegicus dual specificity phos | chr10 | 0.036619 | 2.941988 | 14.54158 | 13.9253 | 13.14952 | 12.43283 | 12.41399 | 12.09921 |
| NM_001039020 | Zfp207 | Rattus norvegicus zinc finger protein 2 | chr10 | 0.004481 | 2.1857624 | 13.26428 | 13.1136 | 13.12858 | 11.99361 | 12.10135 | 12.02714 |
| NM_001013159 | Fnbp4 | formin binding protein 4 | chr3 | 0.040146 | 2.437995 | 12.21784 | 12.0774 | 11.88004 | 10.42201 | 11.17551 | 10.72068 |
| NM_053365 | Fabp4 | Rattus norvegicus fatty acid binding pr | chr2 | 0.044384 | 5.6483836 | 11.33743 | 10.68 | 10.73727 | 7.859696 | 8.260496 | 9.140945 |
| NM_001108515 | Fbxl11 | F-box and leucine-rich repeat protein 1 | chr1 | 0.001922 | 2.5385969 | 11.70733 | 12.0877 | 11.87676 | 10.25083 | 10.76901 | 10.6199 |
| NM_001106095 | Lig4 | ligase IV, DNA, ATP-dependent" | chr16 | 0.000738 | 44.525875 | 7.996512 | 8.42023 | 8.397246 | 2.817606 | 2.788281 | 2.77839 |
| NM_001114602 | Pcdhb5 | protocadherin beta 5 | chr18 | 0.022399 | 3.263818 | 7.255568 | 7.39098 | 7.195262 | 5.052415 | 5.80034 | 5.869376 |
| NM_019314 | Kcnn2 | Rattus norvegicus potassium intermedi | chr18 | 0.02122 | 4.5301394 | 9.690983 | 10.4166 | 9.812533 | 6.873641 | 8.470915 | 8.03694 |
| NM_001105724 | Rasa2 | RAS p21 protein activator 2 | chr8 | 0.042812 | 6.7699594 | 6.637554 | 5.96032 | 6.823629 | 2.817606 | 4.179215 | 4.147237 |
| NM_198051 | Mds024 | Rattus norvegicus putative RNA methy | chr1 | 0.015035 | 7.523384 | 6.833575 | 7.23094 | 8.092716 | 4.62694 | 4.103573 | 4.692576 |
| NM_001107357 | Arhgap12 | Rho GTPase activating protein 12 | chr17 | 0.021792 | 2.270554 | 9.924163 | 10.5305 | 9.77367 | 8.414223 | 9.39072 | 8.874217 |
| NM_001047088 | Gpr120_pr | Rattus norvegicus G protein-coupled re | chr1 | 0.027538 | 2.6844993 | 9.352034 | 9.69281 | 9.383314 | 8.064412 | 8.600619 | 7.48917 |
| NM_017042 | Ppp3cb | Rattus norvegicus protein phosphatase | chr15 | 0.002356 | 5.1995835 | 10.5209 | 10.9966 | 11.06926 | 8.170504 | 8.405347 | 8.875707 |
| NM_001103354 | LOC10012 | hypothetical protein LOC100125362 | chr8 | 0.043339 | 2.2887034 | 8.842094 | 9.8715 | 8.681237 | 7.38424 | 8.426123 | 8.000877 |
| NM_001107670 | Phf17 | PHD finger protein 17 | chr2 | 0.04674 | 2.1372216 | 12.29032 | 12.7599 | 11.91274 | 10.99297 | 11.37713 | 11.30569 |
| NM_001012003 | Orc2l | Rattus norvegicus origin recognition co | chr9 | 0.028706 | 7.907238 | 9.399775 | 8.68823 | 9.786214 | 6.340062 | 6.635692 | 5.948942 |
| NM_001114603 | Pcdhb10 | protocadherin beta 10 | chr18 | 0.025921 | 5.417701 | 7.417662 | 7.07166 | 7.096586 | 4.439827 | 5.415785 | 4.417249 |
| NM_001134800 | Zbed4 | zinc finger, BED-type containing 4" | chr7 | 0.028513 | 2.2009864 | 9.298307 | 9.70995 | 8.976152 | 7.80925 | 8.594345 | 8.166362 |
| NM_001014029 | RGD13103 | Rattus norvegicus similar to RIKEN cI | chr1 | 0.004181 | 2.204656 | 11.40302 | 12.0514 | 11.72169 | 10.35752 | 10.96149 | 10.43541 |
| NM_001013989 | RGD13095 | Rattus norvegicus similar to RNA-binc | chr14 | 0.011169 | 4.7126184 | 9.132239 | 9.11954 | 9.614522 | 6.624214 | 7.358117 | 7.17438 |
| NM_001105718 | Vps35 | maternal embryonic message 3 | chr19 | 0.000749 | 2.440554 | 13.99612 | 14.2091 | 14.07168 | 12.76343 | 12.85595 | 12.79594 |
| NM_001108546 | Ythdf3 | YTH domain family, member 3" | chr2 | 0.001338 | 2.0306547 | 11.67303 | 11.7047 | 11.75056 | 10.60182 | 10.75621 | 10.70448 |
| NM_001108260 | Baz2b | bromodomain adjacent to zinc finger d | chr3 | 0.000121 | 38.68613 | 8.196045 | 8.76598 | 8.247255 | 2.817606 | 3.501533 | 3.068909 |
| NM_001047846 | LOC28816 | Rattus norvegicus similar to PEST-con | chr11 | 0.019124 | 5.41375 | 7.233537 | 7.06664 | 9.215589 | 4.419087 | 5.312467 | 6.474328 |
| NM_001108631 | Herc3 | hect domain and RLD 3 | chr4 | 0.017425 | 2.722581 | 10.15063 | 10.7099 | 10.36047 | 8.430924 | 9.637757 | 8.817364 |
| NM_001037310 | Taf9 | TAF9 RNA polymerase II, TATA box | chr2 | 0.00957 | 2.0735736 | 12.86377 | 13.2874 | 12.76715 | 11.71471 | 12.44245 | 11.60478 |
| NM_001108355 | Cnot6l | CCR4-NOT transcription complex, sul | chr14 | 0.000704 | 11.792442 | 8.183004 | 8.74188 | 8.53857 | 4.43844 | 5.308956 | 5.036691 |

| | | | | | | | | | | | |
|---|---|---|---|---|---|---|---|---|---|---|---|
| NM_001082541 | Hnrpd | heterogeneous nuclear ribonucleoprote | chr14 | 0.00542 | 3.8694472 | 13.36373 | 13.3307 | 13.16674 | 11.12981 | 11.57351 | 11.30151 |
| NM_001130039 | Dhx32 | DEAH (Asp-Glu-Ala-His) box polypep | chr1 | 0.012149 | 3.6717708 | 5.271543 | 4.99188 | 5.86277 | 3.180668 | 3.532829 | 3.783271 |
| NM_001013202 | Clca2 | Rattus norvegicus chloride channel cal | chr2 | 0.022842 | 8.603895 | 7.606926 | 7.27984 | 8.257955 | 5.047053 | 4.581357 | 4.201343 |
| NM_053637 | Stxbp3 | Rattus norvegicus syntaxin binding pro | chr2 | 0.006909 | 2.2547476 | 11.74406 | 12.028 | 11.45819 | 10.38463 | 11.0006 | 10.3261 |
| NM_199106 | Galnt13 | Rattus norvegicus UDP-N-acetyl-alpha | chr3 | 0.013849 | 3.196628 | 8.854977 | 10.2562 | 9.645447 | 6.935052 | 8.427741 | 8.364129 |
| NM_001108592 | Snrpb2 | small nuclear ribonucleoprotein polype | chr3 | 0.026136 | 2.5677626 | 12.08819 | 12.064 | 11.9384 | 10.39184 | 11.12917 | 10.48806 |
| NM_001039035 | Sfrs7 | splicing factor, arginine/serine-rich 7" | chr6 | 0.032261 | 3.4123132 | 10.54573 | 10.4982 | 11.1638 | 8.159821 | 9.222135 | 9.513555 |
| NM_001106406 | Ppwd1 | peptidylprolyl isomerase domain and V | chr2 | 0.031622 | 4.3963385 | 5.485325 | 5.34653 | 6.477655 | 3.881346 | 3.435863 | 3.583392 |
| NM_001012050 | Fbxo8 | Rattus norvegicus F-box only protein 8 | chr16 | 0.00443 | 2.376101 | 12.1 | 12.2908 | 12.3 | 10.68996 | 11.15911 | 11.09593 |
| NM_001012006 | Ace2 | Rattus norvegicus angiotensin I conver | chrX | 0.037484 | 5.49452 | 11.2111 | 10.6167 | 11.35472 | 8.592465 | 9.07583 | 8.14023 |
| NM_001106480 | Prpf40a | PRP40 pre-mRNA processing factor 40 | chr3 | 0.018556 | 5.401561 | 10.80538 | 11.3889 | 11.10325 | 8.962976 | 8.937713 | 8.096736 |
| NM_001013135 | Sdccag3 | Rattus norvegicus serologically define | chr3 | 0.011644 | 2.84585 | 10.29686 | 10.28 | 10.00676 | 8.563406 | 9.091095 | 8.402576 |
| NM_001025747 | Yipf6 | Rattus norvegicus Yip1 domain family | chrX | 0.010266 | 4.6730433 | 9.682591 | 9.7376 | 10.1732 | 7.187167 | 7.964458 | 7.768688 |
| NM_017067 | Chm | Rattus norvegicus choroidermia (Chm) | chrX | 0.016841 | 5.36594 | 7.275767 | 7.51961 | 8.334339 | 5.365464 | 5.165691 | 5.32707 |
| NM_001107459 | Rgs17 | regulator of G-protein signaling 17 | chr1 | 0.004635 | 8.646503 | 9.353606 | 10.1893 | 9.969966 | 6.006891 | 7.501639 | 6.668022 |
| NM_001014266 | RGD13595 | Rattus norvegicus similar to RIKEN cI | chr7 | 0.013244 | 3.225713 | 10.90433 | 11.0363 | 10.48146 | 8.841111 | 9.638538 | 8.873569 |
| NM_021763 | Arfip1 | Rattus norvegicus ADP-ribosylation fa | chr2 | 0.008624 | 8.799899 | 8.746468 | 8.82361 | 9.934852 | 5.153009 | 6.233614 | 6.70585 |
| NM_001108421 | Upf2 | UPF2 regulator of nonsense transcripts | chr17 | 0.034936 | 5.369233 | 9.071424 | 7.95022 | 9.952715 | 6.644738 | 6.332747 | 6.722729 |
| NM_017301 | Edg1 | Rattus norvegicus endothelial different | chr2 | 0.046703 | 2.9045868 | 11.03362 | 10.8612 | 10.54762 | 8.831817 | 9.816411 | 9.179226 |
| NM_030989 | Tp53 | Rattus norvegicus tumor protein p53 (T | chr10 | 0.037655 | 4.3043175 | 10.52755 | 10.2074 | 9.595755 | 7.94304 | 8.940013 | 7.130309 |
| NM_001013156 | Zcchc9 | Rattus norvegicus zinc finger, CCHC d | chr2 | 0.039196 | 2.5410264 | 9.99226 | 10.1921 | 10.22909 | 8.621129 | 9.334446 | 8.421614 |
| NM_022690 | Ube2g1 | Rattus norvegicus ubiquitin-conjugatin | chr10 | 0.004392 | 2.7349243 | 12.13107 | 12.7877 | 12.35071 | 10.49135 | 11.3932 | 11.03042 |
| NM_012519 | Camk2d | calcium/calmodulin-dependent protein | chr2 | 0.000738 | 12.392486 | 7.241242 | 7.41917 | 7.919259 | 3.478098 | 3.980926 | 4.226468 |
| NM_017132 | Rcn2 | Rattus norvegicus reticulocalbin 2 (Rcn | chr8 | 0.007627 | 2.8386128 | 12.43514 | 12.6796 | 12.34317 | 11.00843 | 11.35383 | 10.58007 |
| NM_001109265 | Zbtb8b | zinc finger and BTB domain containin | chr5 | 0.039666 | 2.8264856 | 5.176072 | 5.08867 | 5.875485 | 3.115124 | 4.088253 | 4.439827 |
| NM_001014004 | Amt | Rattus norvegicus aminomethyltransfe | hr16_randor | 0.046839 | 7.6013603 | 10.06358 | 9.72638 | 9.910639 | 6.141246 | 8.039293 | 6.741282 |
| NM_001135003 | Cwf19l2 | CWF19-like 2, cell cycle control" | chr7 | 0.048864 | 2.9165587 | 5.552588 | 5.84095 | 5.434987 | 3.324562 | 4.47625 | 4.394913 |
| NM_145881 | Rims2 | regulating synaptic membrane exocyto | chr7 | 0.002362 | 6.6228013 | 5.370813 | 5.65605 | 6.411138 | 2.817606 | 3.015043 | 3.423025 |
| NM_013111 | Slc7a1 | Rattus norvegicus solute carrier family | chr12 | 0.022265 | 2.589523 | 10.5883 | 11.2395 | 11.03755 | 9.632247 | 9.656369 | 9.45871 |
| NM_001008337 | Rg9mtd1 | Rattus norvegicus RNA (guanine-9-) m | chr11 | 0.008596 | 2.8977826 | 7.914219 | 8.77571 | 7.595662 | 6.187083 | 7.152871 | 6.340788 |
| NM_080910 | Paics | Rattus norvegicus phosphoribosylamin | chr14 | 0.02342 | 2.4554348 | 13.50302 | 13.5037 | 13.48196 | 12.31512 | 12.4906 | 11.79501 |
| NM_001009665 | Ebag9 | Rattus norvegicus estrogen receptor-bi | chr7 | 0.006425 | 3.4558818 | 11.64853 | 12.0675 | 11.98855 | 9.815975 | 10.54693 | 9.97451 |
| NM_019251 | Bet1 | Rattus norvegicus blocked early in trar | chr4 | 0.042674 | 2.0744634 | 13.46574 | 13.6024 | 13.61014 | 12.35109 | 12.96612 | 12.20283 |

| | | | | | | | | | | | |
|---|---|---|---|---|---|---|---|---|---|---|---|
| NM_001030037 | Capn7 | Rattus norvegicus calpain 7 (Capn7), n | chr16 | 0.004637 | 2.3645766 | 11.06926 | 11.2368 | 11.22587 | 9.829266 | 10.14136 | 9.836553 |
| NM_001107267 | Lats2 | large tumor suppressor 2 | chr15 | 0.022184 | 2.581695 | 11.35294 | 11.7912 | 11.28665 | 9.576555 | 10.56378 | 10.18552 |
| NM_138840 | Tgoln2 | Rattus norvegicus trans-golgi network | chr4 | 0.007731 | 4.267918 | 14.42201 | 14.6641 | 14.37579 | 12.23793 | 12.92685 | 12.01657 |
| NM_001014158 | LOC36148 | Rattus norvegicus similar to KRAB-co | chr1 | 0.016087 | 2.532322 | 10.50854 | 10.9185 | 10.49066 | 9.002172 | 9.399775 | 9.494329 |
| NM_001127680 | Erich1 | glutamate-rich 1 | chr16 | 0.018866 | 2.2389715 | 10.48048 | 10.6037 | 10.33567 | 9.22019 | 9.757252 | 8.953926 |
| NM_001107360 | RGD15601 | hypothetical protein LOC307067 | chr17 | 0.01842 | 10.194828 | 7.121524 | 6.86767 | 7.610384 | 3.77282 | 4.315889 | 3.461573 |
| NM_138845 | Tnrc6b | trinucleotide repeat containing 6B | chr7 | 0.024549 | 4.205841 | 10.98088 | 11.5847 | 10.98565 | 9.491161 | 9.492431 | 8.350418 |
| NM_001126267 | Fam49b | hypothetical protein LOC299909 | chr7 | 0.045622 | 2.3848596 | 11.73538 | 12.0271 | 11.69493 | 9.931565 | 10.98443 | 10.77974 |
| NM_001005888 | Galc | Rattus norvegicus galactosylceramidas | chr6 | 0.005481 | 7.6912203 | 7.402804 | 6.80334 | 7.635646 | 4.085202 | 3.851165 | 5.075791 |
| NM_053990 | Ptpn2 | Rattus norvegicus protein tyrosine pho | chr18 | 0.018673 | 3.9151785 | 11.69395 | 11.7856 | 11.46675 | 9.230584 | 10.26416 | 9.544313 |
| NM_001108128 | Chordc1 | cysteine and histidine-rich domain | chr8 | 0.027355 | 4.1301265 | 6.168352 | 6.88329 | 6.560412 | 3.627298 | 4.666734 | 5.179466 |
| NM_001024310 | Arl6ip6 | ADP-ribosylation-like factor 6 interact | chr3 | 0.005845 | 4.58612 | 10.38322 | 10.7672 | 10.46892 | 7.956381 | 8.470484 | 8.600619 |
| NM_001025277 | RGD1308( | Rattus norvegicus similar to RIKEN cI | chr8 | 0.012365 | 2.0910673 | 9.874255 | 9.94831 | 9.756291 | 8.573598 | 9.032032 | 8.78051 |
| NM_001135858 | Tmtc3 | transmembrane and tetratricopeptide re | chr7 | 0.042574 | 4.2447457 | 10.5776 | 11.1621 | 10.88841 | 8.058969 | 8.620105 | 9.691979 |
| NM_031686 | Scn7a | Rattus norvegicus sodium channel, vol | chr3 | 0.009333 | 2.3068635 | 12.58124 | 13.0522 | 12.73862 | 11.31148 | 11.68263 | 11.76015 |
| NM_001109525 | LOC68911 | hypothetical protein LOC689116 | chr11 | 0.015436 | 2.291245 | 12.02571 | 11.8481 | 12.34713 | 10.83539 | 10.90943 | 10.88772 |
| NM_001134713 | Shq1 | SHQ1 homolog | chr4 | 0.044652 | 2.6345348 | 7.833663 | 8.30888 | 8.421614 | 6.173568 | 7.520689 | 6.677249 |
| NM_001034129 | Tloc1 | Rattus norvegicus translocation proteir | chr2 | 0.00034 | 189.86441 | 10.14107 | 10.3648 | 10.62325 | 2.817606 | 2.788281 | 2.816725 |
| NM_199381 | NAPE-PLI | Rattus norvegicus N-acyl-phosphatidyl | chr4 | 0.038398 | 2.024715 | 6.363915 | 7.14898 | 6.666445 | 4.956954 | 6.212197 | 5.95703 |
| NM_001025738 | Fusip1 | Rattus norvegicus FUS interacting prot | chr5 | 0.023764 | 2.4438899 | 8.536344 | 8.9524 | 8.688231 | 6.84528 | 7.905543 | 7.558619 |
| NM_001009698 | Lysmd3 | Rattus norvegicus LysM, putative pept | chr2 | 0.039157 | 3.697425 | 7.87976 | 7.36746 | 9.044413 | 5.618829 | 6.25025 | 6.762991 |
| NM_019272 | Sema4f | Rattus norvegicus sema domain, immu | chr4 | 0.030206 | 2.4004695 | 6.477655 | 6.52519 | 6.289237 | 4.792056 | 5.339952 | 5.370127 |
| NM_053476 | Synj1 | synaptojanin 1 | chr11 | 0.010395 | 3.3250797 | 10.81312 | 10.9119 | 10.88436 | 8.89758 | 9.004471 | 9.507181 |
| NM_017339 | Isl1 | Rattus norvegicus ISL1 transcription fa | chr2 | 0.030904 | 9.548457 | 8.239742 | 7.75925 | 9.264771 | 5.109515 | 5.450537 | 4.937913 |
| NM_001106400 | Ankrd32 | ankyrin repeat domain 32 | chr2 | 0.00626 | 3.9569383 | 10.26086 | 10.5539 | 10.12941 | 7.976301 | 8.635492 | 8.379237 |
| NM_001109339 | Eif2a | eukaryotic translation initiation factor 2 | chr2 | 0.021332 | 2.0258963 | 12.17155 | 12.2993 | 12.45705 | 11.07687 | 11.57229 | 11.2231 |
| NM_001108028 | L2hgdh | L-2-hydroxyglutarate dehydrogenase | chr6 | 0.015648 | 2.9285154 | 8.884852 | 9.17477 | 8.805302 | 7.077056 | 8.0098 | 7.127564 |
| NM_012650 | Shbg | Rattus norvegicus sex hormone binding | chr10 | 0.025189 | 2.3065045 | 6.477041 | 4.92876 | 5.821532 | 4.881867 | 3.937463 | 4.790876 |
| NM_031807 | Tpbg | Rattus norvegicus trophoblast glycopro | chr8 | 0.027648 | 2.46769 | 7.908388 | 7.97719 | 7.218072 | 6.26923 | 7.091528 | 5.833406 |
| NM_199387 | Mterfd1 | Rattus norvegicus MTERF domain cor | chr7 | 0.009063 | 3.6150398 | 11.89043 | 11.9977 | 11.71134 | 9.880872 | 10.49823 | 9.658342 |
| NM_153310 | St18 | Rattus norvegicus suppression of tumo | chr5 | 0.01814 | 15.637469 | 5.957804 | 7.77492 | 6.995013 | 2.817606 | 2.788281 | 3.221041 |
| NM_021851 | Lin7c | Rattus norvegicus lin-7 homolog C (C. | chr3 | 0.002043 | 3.91896 | 6.935052 | 7.12725 | 6.676713 | 4.914704 | 5.330055 | 4.582842 |
| NM_053520 | Elf1 | Rattus norvegicus E74-like factor 1 (E | chr15 | 0.029807 | 11.901433 | 7.141392 | 6.3292 | 7.700732 | 2.817606 | 4.010175 | 3.62435 |

| | | | | | | | | | | | |
|---|---|---|---|---|---|---|---|---|---|---|---|
| NM_001014191 | RGD13057 | Rattus norvegicus similar to hypothetic | chr5 | 0.02185 | 5.573617 | 8.645481 | 9.23804 | 8.66119 | 5.421841 | 7.130309 | 6.556721 |
| NM_031346 | Rod1 | Rattus norvegicus ROD1 regulator of c | chr5 | 0.046145 | 2.1334705 | 6.352901 | 6.58358 | 6.617353 | 5.297278 | 5.051367 | 5.925581 |
| NM_001034006 | Acap2 | ArfGAP with coiled-coil, ankyrin repea | chr11 | 0.01904 | 4.06584 | 7.455291 | 7.64492 | 7.935471 | 4.935875 | 6.10666 | 5.922489 |
| NM_133602 | Khsrp | Rattus norvegicus KH-type splicing reg | chrUn | 0.006872 | 2.6588244 | 6.807402 | 6.75949 | 6.576551 | 5.459 | 5.513831 | 4.938245 |
| NM_030873 | Pfn2 | Rattus norvegicus profilin 2 (Pfn2), mI | chr2 | 0.002714 | 2.705149 | 12.23475 | 12.5364 | 12.05448 | 10.65959 | 11.12281 | 10.73613 |
| NM_001107639 | Prdm1 | PR domain containing 1, with ZNF dor | chr20 | 0.017127 | 2.630554 | 8.076221 | 8.12917 | 7.479098 | 6.310886 | 6.922545 | 6.264957 |
| NM_001107278 | Fndc3a | fibronectin type III domain containing | chr15 | 0.012494 | 3.447654 | 13.24913 | 13.6077 | 13.33709 | 11.17593 | 12.21034 | 11.45081 |
| NM_001107101 | Arl13b | ADP-ribosylation factor-like 13B | chrUn | 0.028086 | 3.8901725 | 9.826979 | 9.98671 | 10.1079 | 7.651217 | 8.685141 | 7.705729 |
| NM_001109105 | Commd6 | COMM domain containing 6 | chr15 | 0.025851 | 6.3297644 | 10.22227 | 10.0405 | 10.38405 | 6.994176 | 8.237061 | 7.429114 |
| NM_001014197 | RGD13103 | Rattus norvegicus similar to chromosor | chr6 | 0.001046 | 7.508541 | 5.567284 | 6.1235 | 5.678761 | 2.817606 | 3.047949 | 2.77839 |
| NM_001007618 | Rchy1 | Rattus norvegicus ring finger and CHY | chr14 | 0.020808 | 2.0672984 | 12.82356 | 12.934 | 12.76254 | 11.87676 | 12.0869 | 11.41316 |
| NM_024377 | Gng5 | Rattus norvegicus guanine nucleotide b | chr2 | 0.014023 | 2.14274 | 8.147813 | 8.34883 | 8.245097 | 7.033775 | 7.484223 | 6.92537 |
| NM_001024303 | LOC49967 | Rattus norvegicus similar to Lix1 homo | chr2 | 0.027193 | 4.4714236 | 12.29627 | 12.414 | 12.15596 | 9.416333 | 10.51771 | 10.44997 |
| NM_001024789 | Nap1l2 | Rattus norvegicus nucleosome assembl | chrX | 0.013167 | 3.4592624 | 10.96923 | 11.3115 | 11.23365 | 8.768044 | 9.777874 | 9.597047 |
| NM_001100671 | Arl6ip2 | ADP-ribosylation factor-like 6 interact | chr6 | 0.011682 | 2.2201288 | 12.53642 | 12.9388 | 12.37267 | 11.44362 | 11.97072 | 10.98165 |
| NM_001017513 | MGC1089 | Rattus norvegicus similar to replication | chr1 | 0.044504 | 3.2869015 | 6.292809 | 6.35853 | 6.571495 | 5.257669 | 4.030859 | 4.784119 |
| NM_001082549 | Spint2 | serine protease inhibitor, Kunitz type 2 | chr1 | 0.020449 | 2.5879984 | 16.56032 | 16.5155 | 16.32471 | 14.79664 | 15.40231 | 15.08603 |
| NM_001108376 | RGD1308 | hypothetical protein LOC361038 | chr15 | 0.033531 | 4.646611 | 11.08392 | 10.9666 | 10.9666 | 9.079232 | 9.342195 | 7.947165 |
| NM_198748 | Scin | Rattus norvegicus scinderin (Scin), mF | chr6 | 0.016597 | 4.073348 | 6.198278 | 6.15191 | 6.062718 | 3.699062 | 4.566701 | 4.068493 |
| NM_001134879 | Atad2 | ATPase family, AAA domain containi | chr7 | 0.026485 | 2.428512 | 8.953621 | 8.82595 | 8.991162 | 7.403204 | 7.96519 | 7.562117 |
| NM_001127379 | Api5 | apoptosis inhibitor 5 | chr3 | 0.003595 | 2.4256058 | 13.64245 | 13.8872 | 13.55673 | 12.2843 | 12.76254 | 12.20453 |
| NM_001009624 | RGD1307 | Rattus norvegicus similar to RIKEN cI | chr10 | 0.001913 | 2.4413428 | 9.899141 | 10.0979 | 9.91663 | 8.718375 | 8.725602 | 8.606633 |
| NM_001009831 | Spg3a | Rattus norvegicus spastic paraplegia 3 | chr6 | 0.028428 | 2.0008647 | 9.145079 | 9.42594 | 8.98784 | 7.957669 | 8.76972 | 7.829598 |
| NM_013145 | Gnai1 | Rattus norvegicus guanine nucleotide b | chr4 | 0.000549 | 51.99212 | 8.649288 | 8.96068 | 8.77249 | 2.817606 | 3.527558 | 2.936625 |
| NM_001013131 | Ttc5 | Rattus norvegicus tetratricopeptide rep | chr15 | 0.005695 | 7.9647994 | 9.508286 | 9.62782 | 9.46159 | 6.301017 | 6.394298 | 6.921467 |
| NM_001135749 | RGD1561 | RGD1561797 | chr2 | 0.029383 | 3.4191873 | 5.759497 | 7.05157 | 6.736795 | 4.582448 | 4.827732 | 4.816719 |
| NM_001134539 | RGD1308 | hypothetical protein LOC304645 | chr19 | 0.019512 | 3.8837051 | 11.75433 | 12.0509 | 11.56207 | 9.697869 | 9.669995 | 10.12708 |
| NM_001025274 | Sfrs18 | splicing factor, arginine/serine-rich 18' | chr5 | 0.00955 | 2.7844698 | 11.10928 | 11.4654 | 10.87218 | 9.346554 | 10.08188 | 9.586245 |
| NM_001109292 | RGD1564 | hypothetical protein LOC500988 | chr8 | 0.0412 | 2.3091695 | 8.412192 | 8.04093 | 8.413767 | 7.389653 | 7.148978 | 6.70613 |
| NM_001135893 | Usp9x | ubiquitin specific peptidase 9, X-linke | chrX | 0.007785 | 4.931455 | 6.769297 | 7.00594 | 7.574399 | 4.063212 | 4.958543 | 5.421841 |
| NM_001012197 | Tra1_predi | Rattus norvegicus tumor rejection antig | chr7 | 0.020975 | 2.365041 | 16.4431 | 16.7622 | 16.11324 | 15.11703 | 15.25439 | 15.22148 |
| NM_001108984 | Rbm25 | RNA binding motif protein 25 | chr6 | 0.008703 | 3.0959303 | 11.98305 | 12.2098 | 11.89174 | 10.25505 | 10.37696 | 10.56151 |
| NM_021692 | Smad5 | Rattus norvegicus MAD homolog 5 (D | chr17 | 0.033094 | 4.478998 | 9.389341 | 9.3564 | 8.884392 | 6.465764 | 7.808049 | 6.866797 |

| | | | | | | | | | | | |
|---|---|---|---|---|---|---|---|---|---|---|---|
| NM_001105717 | Dpysl2 | dihydropyrimidinase-like 2 | chr15 | 0.009201 | 2.33025 | 14.39271 | 14.4842 | 13.88817 | 13.32218 | 13.34632 | 12.43514 |
| NM_001134970 | LOC69103 | SET binding factor 2 | chr1 | 0.01727 | 2.7090518 | 10.43903 | 9.97689 | 10.42272 | 8.758154 | 8.916826 | 8.850292 |
| NM_001107741 | Med19 | mediator complex subunit 19 | chr3 | 0.039458 | 3.1623435 | 7.654681 | 7.33395 | 8.004587 | 5.957267 | 6.279374 | 5.773593 |
| NM_001100556 | Gtf2e1 | general transcription factor IIE, polype | chr11 | 0.005395 | 4.8047204 | 9.684289 | 9.82681 | 9.754137 | 7.180059 | 7.883601 | 7.40822 |
| NM_001106584 | Rpa3 | replication protein A3 | chr4 | 0.046317 | 2.3338728 | 11.24388 | 11.4231 | 11.52476 | 10.18214 | 10.57122 | 9.770223 |
| NM_001166342 | | | chrX | 0.028895 | 4.08751 | 5.554768 | 5.06736 | 5.675211 | 2.817606 | 3.387176 | 3.998887 |
| NM_001108444 | Gab1 | GRB2-associated binding protein 1 | chr19 | 0.001743 | 5.631074 | 10.63113 | 10.9332 | 10.65311 | 8.116385 | 8.630049 | 7.990779 |
| NM_001039002 | RGD13047 | Rattus norvegicus similar to CG8312-I | chr11 | 0.029907 | 2.828229 | 11.81702 | 12.3481 | 11.92799 | 10.00481 | 10.63274 | 10.95589 |
| NM_001108362 | Tmem128 | transmembrane protein 128 | chr14 | 0.012709 | 5.404768 | 11.61245 | 11.732 | 11.53632 | 8.639215 | 9.455865 | 9.482952 |
| NM_057108 | Serpinb5 | Rattus norvegicus serine (or cysteine) | chr13 | 0.005795 | 2.2787557 | 6.673231 | 5.05777 | 6.013793 | 5.316344 | 3.895276 | 4.968432 |
| NM_031514 | Jak2 | Rattus norvegicus Janus kinase 2 (Jak2 | chr1 | 0.030768 | 3.183899 | 9.130239 | 8.73475 | 9.054214 | 7.115596 | 7.661781 | 7.129435 |
| NM_001106502 | Usp8 | ubiquitin specific peptidase 8 | chr3 | 0.006181 | 2.1465948 | 11.03628 | 11.0278 | 11.30305 | 9.862151 | 10.09902 | 10.0998 |
| NM_012598 | Lpl | Rattus norvegicus lipoprotein lipase (L | chr16 | 0.004516 | 10.004647 | 7.998811 | 8.66648 | 8.278396 | 4.943666 | 4.89882 | 5.133407 |
| NM_175603 | Hnrpr | Rattus norvegicus heterogeneous nucle | chr5 | 0.005018 | 2.7065065 | 11.99471 | 12.1331 | 12.31512 | 10.75572 | 10.55254 | 10.82536 |
| NM_001047889 | Wdr75 | Rattus norvegicus WD repeat domain ` | chr9 | 0.002565 | 3.060958 | 12.24509 | 12.6494 | 12.34231 | 10.68823 | 11.13978 | 10.56681 |
| NM_017307 | Slc25a1 | Rattus norvegicus solute carrier family | chr11 | 0.039507 | 2.038679 | 10.66487 | 11.1991 | 10.02458 | 10.02751 | 9.839366 | 8.938744 |
| NM_001106062 | Ogdhl | oxoglutarate dehydrogenase-like | chr16 | 0.007997 | 2.2642052 | 9.895317 | 10.0755 | 9.916212 | 8.916826 | 8.856273 | 8.576948 |
| NM_031018 | Atf2 | Rattus norvegicus activating transcript | chr3 | 0.011521 | 8.0757 | 6.420606 | 6.36199 | 6.835573 | 3.853947 | 3.536749 | 3.186709 |
| NM_001127503 | LOC10015 | hypothetical protein LOC100151767 | chr8 | 0.001668 | 8.680994 | 8.314182 | 8.60759 | 8.521162 | 5.182286 | 5.717238 | 5.189833 |
| NM_001106448 | Ube2q1 | ubiquitin-conjugating enzyme E2Q fan | chr2 | 0.04878 | 2.08283 | 13.56345 | 13.5646 | 13.64245 | 12.62756 | 12.85152 | 12.11573 |
| NM_031011 | Amd1 | Rattus norvegicus S-adenosylmethionii | chr20 | 0.019932 | 2.5205612 | 11.90639 | 11.7124 | 11.93571 | 10.44418 | 10.75475 | 10.35434 |
| NM_001135857 | Tmco3 | transmembrane and coiled-coil domain | chr16 | 0.039691 | 2.2902167 | 13.14061 | 13.2384 | 13.31788 | 11.94425 | 12.46867 | 11.69755 |
| NM_198779 | RGD73514 | Rattus norvegicus hypothetical protein | chr3 | 0.002206 | 5.2974806 | 10.16496 | 10.2298 | 9.668881 | 7.536429 | 7.968303 | 7.342954 |
| NM_001109611 | Adat1 | adenosine deaminase, tRNA-specific 1 | chr19 | 0.018447 | 3.3560543 | 5.606694 | 5.98918 | 6.085827 | 3.799408 | 4.686074 | 3.955924 |
| NM_001047917 | Arsk | Rattus norvegicus arylsulfatase K (Ars | chr2 | 0.009547 | 2.4330378 | 6.850761 | 7.06577 | 6.805987 | 5.528763 | 5.586623 | 5.758853 |
| NM_001025016 | RGD13091 | Rattus norvegicus similar to RIKEN cI | chr14 | 0.002839 | 14.31006 | 9.14232 | 9.44814 | 9.51683 | 4.999121 | 5.999289 | 5.59201 |
| NM_001108684 | Pum1 | pumilio homolog 1 | chr5 | 0.033518 | 2.5413387 | 11.01754 | 11.936 | 11.16993 | 9.684289 | 10.14658 | 10.25581 |
| NM_001025271 | Sfpq | Rattus norvegicus splicing factor prolii | chr5 | 0.004121 | 3.1137514 | 11.18898 | 11.4867 | 11.79739 | 9.758863 | 9.771807 | 10.02641 |
| NM_198791 | Tlr3 | Rattus norvegicus toll-like receptor 3 ( | chr16 | 0.035901 | 2.1061578 | 9.651981 | 9.38515 | 9.663204 | 8.207462 | 8.665154 | 8.603874 |
| NM_001037363 | Lrrn1 | Rattus norvegicus leucine rich repeat n | chr4 | 0.04743 | 5.29106 | 10.07553 | 10.315 | 9.590432 | 6.623757 | 8.681237 | 7.465316 |
| NM_001013223 | Pcbp2 | Rattus norvegicus poly(rC) binding pro | chr7 | 0.010404 | 4.7570424 | 14.21341 | 14.1137 | 14.31349 | 11.88897 | 12.29627 | 11.70517 |
| NM_053345 | Gtf2a2 | Rattus norvegicus general transcriptior | chr8 | 0.008682 | 4.34216 | 12.47417 | 12.5346 | 12.55262 | 10.05105 | 10.78941 | 10.36566 |
| NM_001009635 | RGD13064 | Rattus norvegicus similar to RIKEN cI | chr15 | 0.005447 | 2.0090992 | 12.50448 | 12.7489 | 12.69847 | 11.40485 | 11.88987 | 11.6375 |

| | | | | | | | | | | | |
|---|---|---|---|---|---|---|---|---|---|---|---|
| NM_013001 | Pax6 | Rattus norvegicus paired box gene 6 (I | chr3 | 0.02273 | 2.2941215 | 14.45847 | 14.8493 | 14.50941 | 12.98472 | 13.57892 | 13.65972 |
| NM_001135845 | Rpusd2 | RNA pseudouridylate synthase domair | chr3 | 0.009606 | 10.1933975 | 8.93303 | 9.72581 | 8.630049 | 5.238468 | 7.037409 | 4.964323 |
| NM_001008338 | Lrrc8d | Rattus norvegicus leucine rich repeat c | chr14 | 0.01852 | 2.5115864 | 12.08998 | 12.1681 | 11.20115 | 10.39483 | 11.03253 | 10.04607 |
| NM_001107978 | RGD1559( | hypothetical protein LOC313581 | chr5 | 0.028861 | 2.3027558 | 10.39042 | 10.6108 | 10.33732 | 8.809204 | 9.751057 | 9.168142 |
| NM_001037181 | RGD1308: | Rattus norvegicus similar to KIAA171 | chr16 | 0.026893 | 4.9212885 | 10.03938 | 10.0604 | 9.643868 | 7.007357 | 8.331184 | 7.508026 |
| NM_001127451 | Fam103a1 | hypothetical protein LOC293058 | chr1 | 0.00714 | 3.369356 | 10.60241 | 10.5085 | 10.92274 | 8.640416 | 9.044046 | 9.091814 |
| NM_133535 | Cbwd1 | Rattus norvegicus COBW domain cont | chr1 | 0.016377 | 5.2230134 | 8.689567 | 8.6014 | 9.133694 | 5.730999 | 6.702412 | 6.836601 |
| NM_001105791 | Pfas | phosphoribosylformylglycinamidine sy | chr10 | 0.016876 | 4.919104 | 9.465833 | 9.51356 | 9.315891 | 7.129435 | 7.756931 | 6.513727 |
| NM_001107377 | Rkhd2 | ring finger and KH domain containing | chr18 | 0.027088 | 5.0072303 | 10.6674 | 11.1772 | 10.20462 | 7.682359 | 8.823612 | 8.57124 |
| NM_001106408 | Dimt1l | DIM1 dimethyladenosine transferase 1 | chr2 | 0.042598 | 7.1590843 | 7.470502 | 7.1666 | 7.729201 | 4.306947 | 5.499536 | 4.040495 |
| NM_001040271 | Dut | deoxyuridine triphosphatase isoform 2 | chr3 | 0.036411 | 9.873547 | 9.919311 | 9.42015 | 10.51966 | 6.532198 | 7.27875 | 6.13746 |
| NM_134443 | Creb1 | Rattus norvegicus cAMP responsive el | chr9 | 0.018094 | 2.3195121 | 7.478205 | 7.60139 | 7.534012 | 5.968844 | 6.664533 | 6.338765 |
| NM_053394 | Klf5 | Rattus norvegicus Kruppel-like factor : | chr15 | 0.01961 | 2.6014962 | 7.804695 | 8.46833 | 7.749944 | 6.123281 | 7.023527 | 6.738139 |
| NM_001000163 | Olr143_pr | Rattus norvegicus olfactory receptor 1 | chr1 | 0.018499 | 3.708337 | 7.38424 | 6.99819 | 7.884271 | 5.651341 | 5.459 | 5.484039 |
| NM_001106930 | Efcab1 | EF hand calcium binding domain 1 | chrUn | 0.027938 | 32.43318 | 6.890845 | 9.23471 | 9.712735 | 3.515012 | 3.814957 | 3.450124 |
| NM_001107425 | Nfat5 | nuclear factor of activated T-cells 5 | chr19 | 0.049836 | 2.245787 | 13.489 | 13.3257 | 12.98356 | 11.78231 | 12.46977 | 12.04447 |
| NM_053592 | Dut | Rattus norvegicus deoxyuridine tripho | chr3 | 0.030843 | 7.223929 | 10.11069 | 9.69652 | 10.88207 | 7.501172 | 7.585225 | 7.044528 |
| NM_080477 | Pfkfb2 | Rattus norvegicus 6-phosphofructo-2-k | chr13 | 0.012601 | 5.5035896 | 11.35347 | 11.8046 | 11.56447 | 8.555905 | 9.897474 | 8.888035 |
| NM_001106109 | Nrsn1 | neurensin 1 | chr17 | 0.04436 | 3.4419007 | 8.403764 | 9.30381 | 8.51788 | 7.285011 | 6.83913 | 6.751693 |
| NM_001033066 | Ddhd1 | Rattus norvegicus DDHD domain cont | chr15 | 0.029331 | 5.7732363 | 7.911369 | 8.79323 | 7.344451 | 5.457089 | 5.461779 | 5.542043 |
| NM_017353 | Slc7a5 | Rattus norvegicus solute carrier family | chr19 | 0.029063 | 11.05177 | 8.659582 | 8.77614 | 9.536239 | 5.441183 | 6.210236 | 4.921926 |
| NM_001042561 | Wsb1 | WD repeat and SOCS box-containing | chr10 | 0.034194 | 32.57413 | 11.40998 | 10.7005 | 10.51459 | 5.880923 | 7.520288 | 4.146868 |
| NM_133620 | Zhx1 | Rattus norvegicus zinc-fingers and hor | chr7 | 0.000384 | 3.2760339 | 11.42538 | 12.0229 | 11.45372 | 9.658342 | 10.37163 | 9.736169 |
| NM_017051 | Sod2 | Rattus norvegicus superoxide dismutas | chr1 | 0.006035 | 2.8074358 | 10.22023 | 9.91717 | 10.7457 | 8.631494 | 8.659582 | 9.124264 |
| NM_173102 | Tubb5 | Rattus norvegicus tubulin, beta 5 (Tubl | chr20 | 0.035821 | 14.505712 | 11.81354 | 10.9914 | 11.79005 | 6.615365 | 8.389467 | 8.014552 |
| NM_133563 | Giot1 | Rattus norvegicus gonadotropin induci | chr7 | 0.044982 | 4.0821276 | 9.060693 | 8.63677 | 9.004471 | 6.218307 | 7.329894 | 7.065765 |
| NM_139094 | Rbm16 | Rattus norvegicus RNA binding motif | chr1 | 0.013518 | 2.2671647 | 12.39362 | 12.8427 | 12.15885 | 11.02416 | 11.57992 | 11.24846 |
| NM_001107252 | Nkiras1 | NFKB inhibitor interacting Ras-like 1 | chr15 | 0.009505 | 4.341559 | 8.778739 | 9.16997 | 8.967615 | 6.378282 | 7.45754 | 6.725862 |
| NM_001107700 | Prcc | papillary renal cell carcinoma | chr2 | 0.00451 | 2.272932 | 5.740455 | 6.76856 | 6.545516 | 4.591264 | 5.431507 | 5.478092 |
| NM_181770 | Gpr119 | Rattus norvegicus G protein-coupled r | chrX | 0.002851 | 2.8711798 | 11.69193 | 12.1385 | 12.24028 | 10.24605 | 10.45412 | 10.8056 |
| NM_013022 | Rock2 | Rattus norvegicus Rho-associated coil | chr6 | 0.004779 | 11.049977 | 6.382565 | 6.61233 | 6.280699 | 2.817606 | 2.788281 | 3.271793 |
| NM_021741 | Ip63 | Rattus norvegicus IP63 protein (Ip63), | chr1 | 0.039318 | 2.1736505 | 9.066168 | 9.29879 | 9.534925 | 7.531853 | 8.216846 | 8.790823 |
| NM_001107645 | Rhobtb3 | Rho-related BTB domain containing 3 | chr2 | 0.035233 | 8.295093 | 9.737495 | 10.313 | 10.12993 | 6.621007 | 8.310796 | 6.091839 |

| | | | | | | | | | | | |
|---|---|---|---|---|---|---|---|---|---|---|---|
| NM_175582 | Id4 | Rattus norvegicus inhibitor of DNA bi | chr17 | 0.042006 | 3.74084 | 8.324174 | 7.5924 | 7.164081 | 5.8189 | 6.453973 | 5.097699 |
| NM_001108048 | Rps6ka5 | ribosomal protein S6 kinase, polypepti | chr6 | 0.000522 | 2.9713554 | 9.457809 | 10.2499 | 9.405431 | 7.958495 | 8.642495 | 7.798802 |
| NM_001105725 | Ccng2 | cyclin G2 | chr14 | 0.035575 | 8.121009 | 8.45917 | 7.80617 | 8.917659 | 4.946552 | 5.951003 | 5.22047 |
| NM_001107412 | Hmgxb4 | HMG box domain containing 4 | chr19 | 0.029797 | 2.9930005 | 9.944202 | 10.2164 | 9.956018 | 8.177417 | 9.183824 | 8.010633 |
| NM_001134710 | Cnpy3 | canopy 3 homolog | chr9 | 0.02173 | 2.076573 | 8.075281 | 7.28201 | 8.269549 | 7.329894 | 6.131327 | 7.003002 |
| NM_001131012 | Mier1 | mesoderm induction early response 1 | chr5 | 0.016035 | 2.2002604 | 13.3545 | 13.2521 | 13.18864 | 12.01866 | 12.39887 | 11.96474 |
| NM_012667 | Tacr1 | Rattus norvegicus tachykinin receptor | chr4 | 0.023759 | 2.8467631 | 5.009414 | 5.39954 | 4.689205 | 3.837275 | 3.433381 | 3.299532 |
| NM_001005555 | Ptbp2 | polypyrimidine tract binding protein 2 | chr2 | 0.012593 | 15.9229765 | 8.607228 | 8.97744 | 8.707438 | 5.246602 | 4.107843 | 4.958543 |
| NM_172332 | Wrnip1 | Rattus norvegicus Werner helicase inte | chr17 | 0.038691 | 3.1658394 | 10.9609 | 11.0916 | 11.24527 | 9.479206 | 9.900675 | 8.930143 |
| NM_053927 | Epb4.1l3 | Rattus norvegicus erythrocyte protein l | chr9 | 0.024917 | 9.929059 | 11.55752 | 12.3708 | 11.42862 | 7.420331 | 8.888507 | 9.113148 |
| NM_134353 | Pabpc1 | Rattus norvegicus poly(A) binding pro | chr7 | 0.01081 | 2.5402822 | 15.31177 | 15.6327 | 15.6683 | 14.16195 | 14.36634 | 14.04947 |
| NM_001107810 | Pcmtd2 | protein-L-isoaspartate (D-aspartate) | chr3 | 0.009109 | 16.357727 | 11.80704 | 12.6598 | 11.46393 | 7.207883 | 8.454321 | 8.172881 |
| NM_001134592 | RGD1565( | hypothetical protein LOC365834 | chr2 | 0.032275 | 2.390056 | 8.12442 | 7.80548 | 7.899519 | 6.422875 | 6.882718 | 6.752689 |
| NM_001004229 | Nup35 | Rattus norvegicus nucleoporin 35 (Nu | chr3 | 0.018023 | 2.6774087 | 11.09117 | 11.4208 | 11.38241 | 9.591857 | 10.3672 | 9.672857 |
| NM_001108238 | Ankrd12 | ankyrin repeat domain 12 | chr9 | 0.006684 | 7.0656075 | 7.378676 | 7.5002 | 7.530013 | 4.421073 | 4.364197 | 5.161177 |
| NM_001127558 | Zfp2 | zinc finger protein 2 | chr10 | 0.001295 | 3.7860987 | 7.806991 | 8.51821 | 8.435009 | 5.988857 | 6.626653 | 6.382565 |
| NM_199403 | Dd25 | Rattus norvegicus hypothetical protein | chr13 | 0.020964 | 5.981562 | 6.050894 | 5.54957 | 6.42964 | 3.801785 | 3.39493 | 3.09182 |
| NM_031805 | Ank3 | Rattus norvegicus ankyrin 3, epithelial | chr20 | 0.004671 | 6.473666 | 8.269028 | 8.22521 | 9.532278 | 5.494554 | 5.883091 | 6.565125 |
| NM_001014135 | Wdr1 | Rattus norvegicus WD repeat domain | chr14 | 0.045319 | 8.080029 | 11.31897 | 11.1601 | 11.77134 | 7.556758 | 7.56822 | 10.08236 |
| NM_053868 | Nlgn1 | Rattus norvegicus neuroligin 1 (Nlgn1) | chr2 | 0.008775 | 3.1618648 | 5.084736 | 5.36295 | 5.932302 | 3.423025 | 3.973888 | 4.000745 |
| NM_001107795 | RGD1305( | hypothetical protein LOC311575 | chr3 | 0.013166 | 3.489942 | 7.099766 | 6.20656 | 7.8581 | 5.713562 | 4.170459 | 5.870798 |
| NM_001012169 | Zfp143 | Rattus norvegicus zinc finger protein 1 | chr1 | 0.024245 | 2.5516305 | 9.371103 | 9.73409 | 9.426423 | 7.662482 | 8.766549 | 8.048329 |
| NM_001105900 | Cggbp1 | CGG triplet repeat binding protein 1 | chr11 | 0.000558 | 2.128009 | 12.57054 | 12.8363 | 12.38156 | 11.46354 | 11.71361 | 11.34275 |
| NM_022593 | Tceb1 | Rattus norvegicus transcription elonga | chr5 | 0.016252 | 2.0039172 | 13.55673 | 13.5176 | 13.53029 | 12.48566 | 12.76512 | 12.34535 |
| NM_001009405 | Arhgap29 | Rattus norvegicus Rho GTPase activat | chr2 | 0.008229 | 18.221859 | 7.050006 | 6.29258 | 7.604488 | 2.817606 | 2.788281 | 2.77839 |
| NM_001108953 | Zbtb6 | zinc finger and BTB domain containin | chr3 | 0.023077 | 4.756698 | 6.122735 | 6.72069 | 6.754221 | 4.556331 | 4.01691 | 4.274524 |
| NM_001108448 | Terf2 | telomeric repeat binding factor 2 | chr19 | 0.012331 | 4.0099783 | 10.03614 | 10.0851 | 9.56892 | 7.725358 | 8.518895 | 7.435151 |
| NM_001047102 | Igsf4d_pre | Rattus norvegicus immunoglobulin sup | chr11 | 0.007153 | 8.6842785 | 6.094378 | 7.01081 | 7.013773 | 2.817606 | 4.409919 | 3.53622 |
| NM_001012155 | Tm9sf1 | Rattus norvegicus transmembrane 9 su | chr15 | 0.032134 | 3.0695856 | 9.438466 | 9.75181 | 9.132476 | 7.311136 | 8.654059 | 7.503427 |
| NM_001134766 | Ccdc62 | coiled-coil domain containing 62 | chr12 | 0.034006 | 6.029298 | 10.56544 | 10.4068 | 9.882049 | 7.086658 | 8.621854 | 7.369823 |
| NM_001106951 | Cul4b | cullin 4B | chrX | 0.016042 | 7.1699915 | 7.261727 | 7.92969 | 8.130842 | 3.753581 | 5.676559 | 5.366205 |
| NM_001107944 | Klhl9 | kelch-like 9 | chr5 | 0.021852 | 46.55661 | 11.99161 | 12.5012 | 11.85737 | 5.779829 | 5.975444 | 7.972129 |
| NM_012879 | Slc2a2 | Rattus norvegicus solute carrier family | chr2 | 0.01289 | 17.318106 | 14.98968 | 15.6098 | 14.92506 | 10.78518 | 10.7275 | 11.66922 |

| | | | | | | | | | | | |
|---|---|---|---|---|---|---|---|---|---|---|---|
| NM_001004280 | RGD1303 | Rattus norvegicus similar to RIKEN cI | chr3 | 0.036451 | 2.063889 | 13.60899 | 13.5263 | 13.26155 | 12.66202 | 12.77688 | 11.82187 |
| NM_133297 | 15-Sep | Rattus norvegicus selenoprotein (Sep1 | chr2 | 0.034368 | 2.663555 | 15.54233 | 15.7135 | 15.70729 | 14.23808 | 14.70179 | 13.78315 |
| NM_001107368 | Bmi1 | BMI1 polycomb ring finger oncogene | chr17 | 0.010547 | 4.846551 | 6.855284 | 6.73271 | 8.122845 | 4.771934 | 4.73118 | 5.376848 |
| NM_001037648 | Fam105a | hypothetical protein LOC310190 | chr2 | 0.017003 | 12.595071 | 7.126393 | 6.32354 | 6.922181 | 2.817606 | 3.610863 | 2.979285 |
| NM_001126286 | RGD1565 | hypothetical protein LOC317344 | chrX | 0.020761 | 2.6966755 | 10.87626 | 10.8171 | 10.33019 | 9.149211 | 9.79077 | 8.790018 |
| NM_001007701 | Tram1 | Rattus norvegicus translocation associ | chr5 | 0.031614 | 3.2590458 | 13.39611 | 13.482 | 13.79482 | 11.83949 | 12.22589 | 11.49417 |
| NM_001139465 | Tmem59 | transmembrane protein 59 | chr5 | 0.022353 | 2.5205746 | 14.99957 | 15.0186 | 15.07082 | 13.51901 | 14.08571 | 13.48299 |
| NM_001034107 | Ing3 | Rattus norvegicus inhibitor of growth f | chr4 | 0.03734 | 2.725107 | 8.168207 | 8.47468 | 8.738201 | 6.148832 | 7.357735 | 7.535587 |
| NM_001012181 | Elf2 | Rattus norvegicus E74-like factor 2 (E | chr2 | 0.005073 | 3.4861135 | 10.38642 | 10.7977 | 10.2006 | 8.718912 | 9.119538 | 8.141422 |
| NM_001012042 | Xkr6 | Rattus norvegicus X Kell blood group | chr15 | 0.04819 | 2.9129155 | 5.444879 | 5.47053 | 6.494734 | 3.518225 | 4.629881 | 4.634638 |
| NM_001013058 | Rbm17 | Rattus norvegicus RNA binding motif | chr17 | 0.038495 | 2.0622852 | 12.50117 | 12.4732 | 12.39873 | 11.10325 | 11.40638 | 11.73077 |
| NM_001037315 | Nat1 | N-acetyltransferase 1 | chr16 | 0.02471 | 3.8814044 | 9.213644 | 9.16852 | 9.158693 | 6.830791 | 7.823045 | 7.017284 |
| NM_001106637 | Gem | GTP binding protein (gene overexpres | chr5 | 0.011827 | 7.3714013 | 10.27402 | 11.0209 | 9.884007 | 8.0098 | 7.947643 | 6.575641 |
| NM_001012096 | Tia1 | Rattus norvegicus cytotoxic granule-as | chr4 | 0.047343 | 3.6693633 | 6.799894 | 6.14725 | 7.478205 | 4.579197 | 5.113711 | 5.105848 |
| NM_001134798 | Dcun1d2 | DCN1, defective in cullin neddylation | chr16 | 0.032389 | 5.0846906 | 6.50107 | 5.51482 | 7.290845 | 3.289346 | 3.591314 | 5.387598 |
| NM_001007626 | Ggps1 | Rattus norvegicus geranylgeranyl diph | chr17 | 0.009214 | 3.8936853 | 8.263061 | 8.95808 | 8.520547 | 6.405713 | 7.260841 | 6.191723 |
| NM_001106942 | Cdx4 | caudal type homeo box 4 | chrX | 0.03247 | 3.333882 | 8.37199 | 8.37647 | 8.91951 | 6.1976 | 7.264785 | 6.993979 |
| NM_001047850 | Thoc1 | Rattus norvegicus THO complex 1 (Th | chr18 | 0.017627 | 5.0834804 | 8.21135 | 7.7289 | 8.100037 | 5.286989 | 5.891067 | 5.82478 |
| NM_001108995 | Zfp26 | zinc finger protein 26 | chr8 | 0.031648 | 5.958191 | 7.780781 | 8.33464 | 7.541268 | 4.334828 | 5.892654 | 5.704585 |
| NM_012506 | Atp1a3 | Rattus norvegicus ATPase, Na+/K+ tra | chr1 | 0.0493 | 2.1679423 | 6.129252 | 5.62321 | 6.699596 | 4.757474 | 5.021852 | 5.323756 |
| NM_031665 | Stx6 | Rattus norvegicus syntaxin 6 (Stx6), m | chr13 | 0.0494 | 2.8604705 | 10.77351 | 11.0175 | 10.98443 | 9.493733 | 9.953827 | 8.77916 |
| NM_001012059 | Mcoln3 | Rattus norvegicus mucolipin 3 (Mcoln | chr2 | 0.02447 | 4.247335 | 6.532867 | 5.96523 | 7.023185 | 4.019956 | 4.533887 | 4.707766 |
| NM_001013154 | Pcgf6 | Rattus norvegicus polycomb group rin | chr1 | 0.024246 | 3.270272 | 9.416654 | 9.8798 | 9.360863 | 7.359841 | 8.704671 | 7.464575 |
| NM_001107986 | Srrm1 | serine/arginine repetitive matrix 1 | chr5 | 0.00335 | 3.9720595 | 11.09733 | 11.2745 | 10.7813 | 9.301539 | 9.079232 | 8.802719 |
| NM_021748 | Nsf | Rattus norvegicus N-ethylmaleimide se | chr10 | 0.029104 | 6.158541 | 8.700826 | 8.51788 | 9.443146 | 5.392227 | 6.762547 | 6.639311 |
| NM_001108360 | Zcchc4 | zinc finger, CCHC domain containing | chr14 | 0.042063 | 3.63692 | 8.944186 | 9.24883 | 8.898077 | 6.645666 | 7.034229 | 7.823045 |
| NM_001109061 | RGD1560 | similar to Cell division protein kinase | chr8 | 0.009547 | 12.239206 | 10.30331 | 10.1994 | 10.57122 | 6.086398 | 7.213638 | 6.933549 |
| NM_001106384 | Zfp280b | zinc finger protein 280b | chr20 | 0.018427 | 2.359659 | 8.700141 | 8.88259 | 8.45038 | 7.160356 | 7.933051 | 7.223965 |
| NM_001107588 | Chuk | conserved helix-loop-helix ubiquitous | chr1 | 0.005653 | 5.7682524 | 10.72851 | 11.1238 | 11.04495 | 8.15327 | 8.947307 | 8.212263 |
| NM_001127456 | Slc48a1 | solute carrier family 48 (heme transpo | chr7 | 0.024905 | 3.1825547 | 5.369781 | 4.6082 | 6.479134 | 4.221285 | 2.788281 | 4.436987 |
| NM_017361 | Nup54 | Rattus norvegicus nucleoporin 54 (Nu | chr14 | 0.020021 | 4.661144 | 6.960442 | 8.03551 | 7.529253 | 4.472077 | 5.447075 | 5.943997 |
| NM_001107669 | Plk4 | polo-like kinase 4 | chr2 | 0.037013 | 5.0065713 | 8.003182 | 8.52747 | 7.505784 | 5.895509 | 5.321234 | 5.848219 |
| NM_001079705 | RGD1311 | Rattus norvegicus similar to 4930506M | chr1 | 0.047062 | 3.379253 | 10.50551 | 10.3806 | 11.25495 | 8.829038 | 9.264771 | 8.777181 |

| | | | | | | | | | | | |
|---|---|---|---|---|---|---|---|---|---|---|---|
| NM_012800 | P2ry1 | Rattus norvegicus purinergic receptor I | chr2 | 0.017793 | 6.760114 | 10.28101 | 11.7223 | 11.30151 | 8.092048 | 8.263061 | 8.678531 |
| NM_001108165 | RGD15607 | hypothetical protein LOC315798 | chr8 | 0.017097 | 3.31656 | 10.22886 | 10.2874 | 10.45836 | 8.500478 | 8.953926 | 8.331184 |
| NM_001100988 | RGD15660 | hypothetical protein LOC498119 | chr11 | 0.049072 | 2.8210459 | 9.021743 | 9.2863 | 8.871008 | 6.841825 | 8.061575 | 7.786961 |
| NM_001142366 | Aqp4 | aquaporin 4 isoform 2 | chr18 | 0.032269 | 2.400433 | 8.222371 | 8.28073 | 7.159315 | 6.60907 | 7.457831 | 5.80563 |
| NM_012588 | Igfbp3 | Rattus norvegicus insulin-like growth f | chr14 | 0.033128 | 2.0311744 | 12.40547 | 11.5423 | 11.77025 | 11.32317 | 10.87642 | 10.45148 |
| NM_001012350 | RGD13099 | Rattus norvegicus hypothetical LOC30 | chr9 | 0.003799 | 2.4027357 | 11.06054 | 11.6069 | 11.27687 | 9.642437 | 10.3928 | 10.11499 |
| NM_001005551 | Ca5b | Rattus norvegicus carbonic anhydrase | chrX | 0.042467 | 5.103632 | 7.544043 | 7.6141 | 8.091504 | 4.201023 | 5.636896 | 6.357148 |
| NM_080896 | Hnrph1 | Rattus norvegicus heterogeneous nucle | chr10 | 0.007701 | 4.122924 | 13.71875 | 13.5616 | 13.56661 | 11.41677 | 11.86516 | 11.434 |
| NM_001025676 | Rbm22 | Rattus norvegicus RNA binding motif | chr18 | 0.027972 | 3.9741712 | 10.43756 | 10.5235 | 9.986713 | 8.399252 | 9.144333 | 7.43225 |
| NM_012736 | Gpd2 | Rattus norvegicus glycerol-3-phosphat | chr3 | 0.036318 | 2.9186933 | 4.61337 | 4.89465 | 6.34333 | 3.23723 | 3.768334 | 4.209819 |
| NM_134356 | Ptprg | Rattus norvegicus protein tyrosine pho | chr15 | 0.046068 | 10.094456 | 7.741387 | 7.41843 | 7.228469 | 3.702909 | 5.565852 | 3.113048 |
| NM_001108539 | Hace1 | HECT domain and ankyrin repeat cont | chr20 | 0.049636 | 2.705082 | 8.904822 | 9.4394 | 8.95446 | 6.847433 | 8.110904 | 8.033327 |
| NM_017065 | Gabrb3 | Rattus norvegicus gamma-aminobutyri | chr1 | 0.012553 | 5.281824 | 6.989184 | 7.49005 | 7.108375 | 4.253364 | 4.886002 | 5.24513 |
| NM_001135708 | Urb2 | URB2 ribosome biogenesis 2 | chr19 | 0.030008 | 2.477433 | 8.201783 | 8.44922 | 8.221266 | 6.585594 | 6.993045 | 7.367094 |
| NM_013130 | Smad1 | Rattus norvegicus MAD homolog 1 (D | chr19 | 0.015266 | 8.3790865 | 9.35519 | 10.0527 | 9.930668 | 5.875843 | 6.632606 | 7.629769 |
| NM_001025142 | Trim35 | Rattus norvegicus tripartite motif prote | chr15 | 0.02406 | 7.9487205 | 14.19125 | 14.0441 | 14.45402 | 11.13803 | 11.90118 | 10.678 |
| NM_019243 | Ptgfrn | Rattus norvegicus prostaglandin F2 rec | chr2 | 0.020837 | 2.70754 | 9.772052 | 8.93874 | 9.32145 | 8.10171 | 7.922389 | 7.697198 |
| NM_001108708 | Snx13 | sorting nexin 13 | chr6 | 0.012002 | 2.7597272 | 8.298 | 8.0691 | 8.85457 | 6.65717 | 6.927944 | 7.242981 |
| NM_199087 | Spint2 | Rattus norvegicus serine protease inhib | chr1 | 0.005391 | 2.7683778 | 16.57739 | 16.7447 | 16.56032 | 14.91667 | 15.45883 | 15.09975 |
| NM_001108658 | Topors | topoisomerase I binding, arginine/serin | chr5 | 0.044176 | 2.4644468 | 10.60542 | 10.7634 | 10.8966 | 9.279181 | 9.964299 | 9.118192 |
| NM_001009720 | Sfrs2 | Rattus norvegicus similar to splicing fa | chr10 | 0.025702 | 3.2308455 | 11.20973 | 11.0663 | 11.45188 | 9.604042 | 9.804479 | 9.243623 |
| NM_001083624 | RGD13070 | Rattus norvegicus similar to KIAA064 | chr14 | 0.043903 | 9.161157 | 6.947517 | 6.08754 | 8.003182 | 2.817606 | 4.244738 | 4.389308 |
| NM_001014136 | Ngly1 | Rattus norvegicus N-glycanase 1 (Ngly | chr15 | 0.020386 | 4.9633284 | 11.89635 | 11.85 | 11.70609 | 9.25754 | 10.2089 | 9.052036 |
| NM_194461 | Pigw | Rattus norvegicus phosphatidylinositol | chr10 | 0.038891 | 3.2915163 | 8.898077 | 9.37314 | 8.94337 | 6.599954 | 8.281876 | 7.176493 |
| NM_022184 | Cask | Rattus norvegicus calcium/calmodulin- | chrX | 0.00974 | 5.612109 | 8.965161 | 9.05366 | 9.022064 | 6.257879 | 7.058875 | 6.258499 |
| NM_001004415 | Tacc2 | Rattus norvegicus transforming, acidic | chr1 | 0.023314 | 3.877764 | 10.69546 | 11.1099 | 10.88175 | 8.901804 | 9.581245 | 8.338338 |
| NM_001108178 | Pls1 | plastin 1 (I isoform) | chr8 | 0.002615 | 5.0089054 | 11.42801 | 12.215 | 12.06809 | 9.30942 | 9.891268 | 9.536922 |
| NM_001017380 | Cyld | ubiquitin carboxyl-terminal hydrolase | chr5 | 0.044056 | 3.4381554 | 6.193294 | 5.23305 | 7.075833 | 3.985373 | 4.223743 | 4.948152 |
| NM_001004418 | Tacc2 | transforming acidic coiled coil 2 isofor | chr1 | 0.025737 | 3.927917 | 10.70796 | 11.0262 | 10.83068 | 8.816662 | 9.56592 | 8.260956 |
| NM_019281 | Gja9 | Rattus norvegicus gap junction membr | chr3 | 0.021094 | 6.464254 | 10.9084 | 11.7571 | 12.0869 | 8.704098 | 9.363587 | 8.607228 |
| NM_001107097 | Zbtb11 | zinc finger and BTB domain containin | chr11 | 0.045875 | 3.0836601 | 7.216842 | 8.4662 | 7.946773 | 5.533201 | 6.24875 | 6.973934 |
| NM_001134581 | RGD13045 | hypothetical protein LOC362461 | chr4 | 0.001343 | 3.3267307 | 11.04207 | 11.3404 | 11.16253 | 9.363587 | 9.677561 | 9.301539 |
| NM_017284 | Psmb2 | Rattus norvegicus proteasome (prosom | chr5 | 0.03736 | 7.7859015 | 11.50036 | 11.2135 | 11.9348 | 9.203625 | 8.763054 | 7.799435 |

| | | | | | | | | | | | |
|---|---|---|---|---|---|---|---|---|---|---|---|
| NM_001009671 | RGD1359[illegible] | Rattus norvegicus similar to hypothetic | chr9 | 0.007575 | 3.1564987 | 10.83765 | 11.3601 | 11.27471 | 9.45871 | 9.630389 | 9.408358 |
| NM_001025053 | Galnt4 | UDP-N-acetyl-alpha-D-galactosamine: | chr7 | 0.046289 | 3.6432436 | 8.833635 | 9.03021 | 8.991673 | 6.168033 | 7.369167 | 7.722645 |
| NM_001014117 | Ublcp1 | Rattus norvegicus ubiquitin-like domai | chr10 | 0.022388 | 2.2672305 | 11.98539 | 12.2371 | 11.8776 | 10.44523 | 11.2471 | 10.86497 |
| NM_001037285 | Hnrnpf | heterogeneous nuclear ribonucleoprote | chr4 | 0.005217 | 3.2630188 | 13.4308 | 13.2993 | 13.38111 | 11.52606 | 11.8203 | 11.64626 |
| NM_001012083 | Snx7 | Rattus norvegicus sorting nexin 7 (Snx | chr2 | 0.047123 | 3.3392992 | 9.548574 | 9.49324 | 9.565187 | 7.442823 | 8.536344 | 7.409196 |
| NM_001007617 | Nuak2 | Rattus norvegicus NUAK family, SNF | chr13 | 0.014517 | 2.3382351 | 8.144176 | 7.78999 | 7.474093 | 6.620303 | 6.706912 | 6.404782 |
| NM_001108415 | Elmo1 | engulfment and cell motility 1 | chr17 | 0.028605 | 2.3203065 | 13.07989 | 13.4873 | 12.90287 | 12.17727 | 12.3607 | 11.28915 |
| NM_001037286 | Hnrnpf | heterogeneous nuclear ribonucleoprote | chr4 | 0.00443 | 3.325482 | 13.41292 | 13.5094 | 13.64188 | 11.63388 | 11.99519 | 11.73444 |
| NM_001099647 | LOC68504 | hypothetical protein LOC685045 | chr1 | 0.023374 | 20.12224 | 9.445875 | 9.36312 | 9.813798 | 5.126826 | 6.193892 | 4.309921 |
| NM_053799 | Dars | Rattus norvegicus aspartyl-tRNA synth | chr13 | 0.049384 | 14.950191 | 12.42515 | 12.7523 | 12.4863 | 9.619673 | 9.539948 | 6.797874 |
| NM_022626 | Phka1 | Rattus norvegicus phosphorylase kinas | chrX | 0.020128 | 2.4000285 | 8.092048 | 8.43562 | 8.291938 | 7.083235 | 6.819964 | 7.12725 |
| NM_001017470 | Cep70 | centrosomal protein 70kDa | chr8 | 9.06E-05 | 7.0624046 | 9.879438 | 10.2758 | 9.874255 | 7.107584 | 7.411212 | 7.050266 |
| NM_173328 | Lgr4 | Rattus norvegicus leucine-rich repeat-c | chr3 | 0.034279 | 7.738055 | 10.40732 | 11.3063 | 10.90131 | 8.546921 | 7.583265 | 7.628853 |
| NM_001012185 | Gpiap1 | Rattus norvegicus GPI-anchored meml | chr3 | 0.023 | 2.0517876 | 13.2792 | 13.661 | 13.29299 | 12.18189 | 12.38217 | 12.5585 |
| NM_001013205 | Myef2 | myelin expression factor 2 | chr3 | 0.044157 | 5.8590426 | 12.89565 | 12.9809 | 12.84822 | 10.63325 | 11.21355 | 9.225972 |
| NM_001098240 | Mpv17l_pr | Rattus norvegicus Mpv17 transgene, k | chr6 | 0.01179 | 2.7250404 | 11.21355 | 10.9692 | 11.28703 | 9.708939 | 9.821865 | 9.600168 |
| NM_001107323 | Tubgcp3 | tubulin, gamma complex associated pr | chr16 | 0.019564 | 9.014532 | 8.950233 | 8.7374 | 9.295324 | 5.763835 | 6.352127 | 5.350234 |
| NM_001107949 | Dnajc6 | DnaJ (Hsp40) homolog, subfamily C, r | chr5 | 0.000712 | 2.2603214 | 12.99044 | 13.6549 | 13.02537 | 11.75153 | 12.50316 | 11.88644 |
| NM_053867 | Tpt1 | Rattus norvegicus tumor protein, transl | chr15 | 0.016536 | 3.6687074 | 16.9144 | 16.5543 | 16.83526 | 14.55789 | 14.84762 | 15.2726 |
| NM_053682 | Yme1l1 | Rattus norvegicus YME1-like 1 (S. cer | chr17 | 0.003514 | 3.128501 | 11.11878 | 11.5626 | 11.4328 | 9.277725 | 10.01605 | 9.884007 |
| NM_001025735 | Tcea1 | Rattus norvegicus transcription elonga | chr5 | 0.024636 | 2.6289477 | 13.22047 | 13.4613 | 13.32566 | 11.66632 | 11.78592 | 12.37169 |
| NM_001007149 | Stau2 | Rattus norvegicus staufen, RNA bindir | chr5 | 0.00435 | 2.6632476 | 9.060294 | 9.34736 | 9.3404 | 7.460131 | 8.023914 | 8.024446 |
| NM_053917 | Inpp4b | Rattus norvegicus inositol polyphospha | chr19 | 0.048622 | 2.1771226 | 5.287956 | 5.58198 | 5.385115 | 3.663292 | 4.616291 | 4.608195 |
| NM_001031654 | Anxa8 | Rattus norvegicus annexin A8 (Anxa8) | chr16 | 0.014072 | 2.125917 | 10.51966 | 7.94445 | 9.161922 | 9.682039 | 6.794411 | 7.885321 |
| NM_001108393 | Zmiz1 | zinc finger, MIZ-type containing 1" | chr16 | 0.002279 | 3.1328428 | 11.87676 | 12.2192 | 11.92719 | 10.1266 | 10.72658 | 10.22758 |
| NM_001108450 | Nudt7 | nudix motif 7 | chr19 | 0.021249 | 2.1037173 | 12.5133 | 12.7299 | 12.45435 | 11.3573 | 11.96426 | 11.1572 |
| NM_001015003 | Crbn | Rattus norvegicus cereblon (Crbn), mR | chr4 | 0.011568 | 19.293474 | 8.122845 | 8.72261 | 9.110968 | 4.687342 | 4.383946 | 4.075017 |
| NM_001105715 | Wnt3 | wingless-type MMTV integration site f | chr10 | 0.042368 | 2.173639 | 11.75398 | 12.0995 | 11.63861 | 10.21867 | 10.98471 | 10.92837 |
| NM_001106173 | Tmem188 | transmembrane protein 188 | chr19 | 0.028696 | 7.5787473 | 9.330767 | 9.41665 | 9.796198 | 6.956574 | 6.957612 | 5.863555 |
| NM_001108512 | Suv420h1 | suppressor of variegation 4-20 homolo | chr1 | 0.042657 | 2.588977 | 10.20312 | 10.6097 | 9.783195 | 8.298 | 9.714673 | 8.466204 |
| NM_001017510 | LOC49875 | Rattus norvegicus similar to cDNA sec | chr17 | 0.015505 | 4.781232 | 11.67805 | 11.5472 | 12.04946 | 9.768003 | 9.506279 | 9.228318 |
| NM_001106838 | RGD1310[illegible] | hypothetical protein LOC300836 | chr8 | 0.026395 | 2.7768123 | 9.809828 | 10.6032 | 10.00539 | 7.860848 | 9.464294 | 8.67301 |
| NM_001109017 | Zfx | zinc finger protein X-linked | chrX | 0.00435 | 3.9465897 | 9.329493 | 9.4875 | 9.528514 | 7.232923 | 7.768453 | 7.402313 |

| | | | | | | | | | | | | |
|---|---|---|---|---|---|---|---|---|---|---|---|---|
| NM_001107984 | Pdik1l | PDLIM1 interacting kinase 1 like | chr5 | 0.024311 | 8.088572 | 8.745965 | 8.71771 | 8.32477 | 5.190099 | 5.286494 | 6.264199 |
| NM_001107109 | Morc3 | microrchidia 3 | chr11 | 0.042371 | 5.6738076 | 10.76996 | 10.9639 | 10.57433 | 7.265913 | 9.277725 | 8.251622 |
| NM_001082539 | Hnrpd | heterogeneous nuclear ribonucleoprote | chr14 | 0.007653 | 3.7161932 | 13.13865 | 13.1072 | 13.10724 | 10.95634 | 11.50217 | 11.21315 |
| NM_001025671 | MGC1144 | Rattus norvegicus similar to Ras assoc | chr14 | 0.021745 | 2.7287447 | 10.71355 | 10.9465 | 10.60796 | 9.346877 | 9.826813 | 8.749573 |
| NM_001134553 | RGD1310 | hypothetical protein LOC312248 | chr4 | 0.009492 | 5.770762 | 7.414208 | 7.54745 | 7.896215 | 5.109108 | 5.290526 | 4.871952 |
| NM_001009542 | MGC7299 | Rattus norvegicus similar to programm | chr2 | 0.025342 | 2.4072762 | 12.54251 | 12.7644 | 12.2843 | 10.97191 | 11.40786 | 11.40928 |
| NM_001106437 | Fhdc1 | FH2 domain containing 1 | chr2 | 0.017498 | 4.326441 | 9.539948 | 10.7657 | 9.9266 | 7.415649 | 8.167599 | 8.309505 |
| NM_001107114 | Usp25 | ubiquitin specific peptidase 25 | chr11 | 0.008419 | 2.4101565 | 10.22721 | 10.5738 | 9.864021 | 8.738765 | 9.342711 | 8.776141 |
| NM_001108122 | Jrkl | jerky homolog-like | chr8 | 0.011544 | 11.48319 | 8.258764 | 8.81774 | 8.354762 | 4.449624 | 6.052438 | 4.364847 |
| NM_001106266 | Tjp1 | tight junction protein 1 | chr1 | 0.020371 | 3.9606097 | 12.23069 | 12.516 | 12.08589 | 9.756676 | 11.03844 | 10.08026 |
| NM_001008398 | Gimap9 | GTPase, IMAP family member" | chr4 | 0.042454 | 3.105718 | 6.882718 | 6.89016 | 7.094288 | 5.734416 | 5.442967 | 4.784998 |
| NM_001107668 | Dcun1d1 | DCN1, defective in cullin neddylation | chr2 | 0.014626 | 7.150016 | 6.07938 | 6.13311 | 7.040376 | 3.179633 | 3.924757 | 3.634632 |
| NM_001008559 | Hapln3 | Rattus norvegicus hyaluronan and prot | chr1 | 0.027274 | 5.4958553 | 6.750128 | 6.76992 | 6.457616 | 3.562373 | 5.017526 | 4.022729 |
| NM_001135039 | Utp18 | UTP18, small subunit processome com | chr10 | 0.017806 | 2.3279629 | 8.865813 | 9.7772 | 8.78407 | 7.352471 | 8.834143 | 7.583265 |
| NM_001109225 | RGD1560 | hypothetical protein LOC500057 | chr4 | 0.004541 | 26.711542 | 7.191296 | 8.16636 | 7.410039 | 2.817606 | 2.788281 | 2.943636 |
| NM_017216 | Slc3a1 | Rattus norvegicus solute carrier family | chr6 | 0.007519 | 17.458876 | 7.107584 | 9.53995 | 8.846254 | 3.477223 | 5.618206 | 4.020691 |
| NM_175765 | Psip1 | Rattus norvegicus PC4 and SFRS1 inte | chr5 | 0.005785 | 6.866813 | 9.019588 | 9.933 | 9.508779 | 6.573382 | 6.758872 | 6.790187 |
| NM_001013099 | Slc38a6 | Rattus norvegicus solute carrier family | chr6 | 0.034206 | 2.438347 | 6.443745 | 5.4375 | 6.08174 | 5.329763 | 4.461413 | 4.314104 |
| NM_001013171 | Gulp1 | Rattus norvegicus GULP, engulfment a | chr9 | 0.03586 | 2.3412604 | 12.9518 | 13.6402 | 12.86137 | 11.57034 | 12.09839 | 12.10279 |
| NM_001107253 | Kctd6 | potassium channel tetramerisation dom | chr15 | 0.01603 | 3.5893493 | 6.547576 | 7.04197 | 6.595568 | 4.726916 | 4.777947 | 5.14908 |
| NM_001013207 | Rnpc2 | Rattus norvegicus RNA-binding regior | chr3 | 0.002552 | 3.5718887 | 14.38139 | 14.3895 | 14.09108 | 12.6446 | 12.63871 | 12.06863 |
| NM_080900 | Afap | Rattus norvegicus actin filament associ | chr14 | 0.00667 | 2.9940653 | 13.70209 | 12.6477 | 13.78384 | 12.1198 | 11.29059 | 11.97691 |
| NM_024144 | Pigm | Rattus norvegicus phosphatidylinositol | chr13 | 0.037735 | 6.4282174 | 8.132242 | 7.32287 | 8.828263 | 4.802728 | 5.704149 | 5.723237 |
| NM_001106738 | RGD1308 | hypothetical protein LOC299127 | chr6 | 0.016204 | 2.395097 | 11.09593 | 11.2128 | 11.04817 | 9.549639 | 9.962861 | 10.06411 |
| NM_139103 | Cd48 | Rattus norvegicus CD48 antigen (Cd4 | chr13 | 0.036851 | 8.997919 | 11.25495 | 10.4015 | 10.7875 | 7.869999 | 8.407475 | 6.657674 |
| NM_134346 | Rap1b | Rattus norvegicus RAS related protein | chr7 | 0.032267 | 3.8860831 | 9.876461 | 9.44694 | 10.96618 | 8.173414 | 7.945057 | 8.296155 |
| NM_013055 | Map3k12 | Rattus norvegicus mitogen activated p | chr7 | 0.032934 | 2.0005844 | 10.72658 | 11.2184 | 10.41511 | 10.0955 | 9.99226 | 9.271097 |
| NM_001108607 | Cse1l | chromosome segregation 1-like | chr3 | 0.034101 | 2.819027 | 10.56877 | 10.6813 | 10.55391 | 8.509734 | 9.417774 | 9.390917 |
| NM_013082 | Sdc2 | Rattus norvegicus syndecan 2 (Sdc2), | chr7 | 0.00941 | 2.808091 | 11.54963 | 11.8002 | 11.61674 | 9.957974 | 10.12563 | 10.41425 |
| NM_022692 | Rab5a | Rattus norvegicus RAB5A, member R | chr9 | 0.042035 | 21.50219 | 7.685744 | 6.75422 | 9.403125 | 2.817606 | 4.12648 | 3.61977 |
| NM_001024305 | Prpf38b | PRP38 pre-mRNA processing factor 3 | chr2 | 0.007349 | 2.9336061 | 11.08641 | 11.139 | 11.304 | 9.474353 | 9.842091 | 9.554964 |
| NM_001108595 | Nat5 | N-acetyltransferase 5 | chr3 | 0.042423 | 2.0608597 | 7.691212 | 8.27024 | 7.705729 | 6.643373 | 6.844788 | 7.049277 |
| NM_057131 | Prpsap2 | Rattus norvegicus phosphoribosyl pyrc | chr10 | 0.014815 | 2.0379095 | 11.79254 | 11.8097 | 11.37129 | 10.52857 | 10.82451 | 10.5392 |

| | | | | | | | | | | | |
|---|---|---|---|---|---|---|---|---|---|---|---|
| NM_198778 | C11orf8h | Rattus norvegicus putative C11orf8 ho | chr3 | 0.008695 | 5.0744424 | 7.17131 | 7.00343 | 7.137209 | 4.655787 | 5.096833 | 4.52958 |
| NM_001037657 | Rbm12 | Rattus norvegicus RNA-binding protei | chr3 | 0.004926 | 2.2090015 | 9.834618 | 10.0411 | 9.793911 | 8.851442 | 8.832106 | 8.555905 |
| NM_001107308 | Gmip | Gem-interacting protein | chr16 | 0.019698 | 8.328025 | 7.929786 | 8.16141 | 8.710856 | 4.692149 | 5.931629 | 5.004356 |
| NM_024385 | Hhex | Rattus norvegicus hematopoietically ex | chr1 | 0.009694 | 3.2162573 | 10.8657 | 10.45 | 10.15018 | 8.889386 | 9.052684 | 8.467636 |
| NM_001037287 | Hnrnpf | heterogeneous nuclear ribonucleoprote | chr4 | 0.000225 | 3.5533664 | 13.33637 | 13.3179 | 13.31058 | 11.45762 | 11.49316 | 11.5265 |
| NM_017015 | Gusb | Rattus norvegicus glucuronidase, beta | chr12 | 0.013621 | 2.2795835 | 10.14884 | 10.1422 | 10.23895 | 8.708282 | 9.186123 | 9.069304 |
| NM_001107954 | Reck | reversion-inducing-cysteine-rich protei | chr5 | 0.018155 | 5.803915 | 11.2682 | 11.5993 | 11.04511 | 8.27432 | 9.742156 | 8.285053 |
| NM_145785 | Hdgfrp3 | Rattus norvegicus hepatoma-derived gr | chr1 | 0.000837 | 2.1775177 | 10.83261 | 11.0951 | 10.68938 | 9.695834 | 10.03444 | 9.518778 |
| NM_001082540 | Hnrpd | heterogeneous nuclear ribonucleoprote | chr14 | 0.003857 | 4.0553193 | 13.11555 | 13.1625 | 12.98849 | 10.846 | 11.24104 | 11.12004 |
| NM_001033951 | Npuk68 | Rattus norvegicus nuclear protein UKp | chr6 | 0.010008 | 3.8820908 | 10.22183 | 10.5898 | 10.52546 | 8.058681 | 8.4449 | 8.962976 |
| NM_032063 | Dll1 | Rattus norvegicus delta-like 1 (Drosop | chr1 | 0.00296 | 16.706535 | 6.476307 | 6.89432 | 7.200674 | 2.817606 | 2.788281 | 2.77839 |
| NM_001033884 | Rexo4 | Rattus norvegicus REX4, RNA exonuc | chr3 | 0.013205 | 11.780249 | 10.71926 | 10.8786 | 10.68414 | 6.833165 | 6.827634 | 7.946339 |
| NM_053490 | Xpo1 | Rattus norvegicus exportin 1, CRM1 h | chr14 | 0.019982 | 2.0394447 | 12.13556 | 12.1599 | 12.25324 | 11.3185 | 11.20478 | 10.94092 |
| NM_053972 | RragB | Rattus norvegicus Ras-related GTP bir | chrX | 0.025581 | 2.5022583 | 12.28688 | 13.0723 | 12.76931 | 11.06027 | 11.33644 | 11.76207 |
| NM_012816 | Amacr | Rattus norvegicus alpha-methylacyl-Co | chr2 | 0.022354 | 2.3438587 | 9.983193 | 10.1977 | 10.09267 | 8.387611 | 9.08949 | 9.109789 |
| NM_001025686 | Sec22l1 | Rattus norvegicus SEC22 vesicle traffi | chr2 | 0.027432 | 4.242504 | 10.44105 | 11.3047 | 11.27453 | 8.657723 | 8.516955 | 9.590896 |
| NM_001012152 | Tbc1d14 | Rattus norvegicus TBC1 domain famil | chr14 | 0.003443 | 2.2173495 | 12.00103 | 12.3 | 11.87268 | 10.75194 | 11.2798 | 10.69546 |
| NM_173290 | Yy1 | Rattus norvegicus YY1 transcription fa | chr6 | 0.025601 | 27.695044 | 9.98999 | 9.82272 | 10.5905 | 5.979052 | 5.814062 | 4.235431 |
| NM_001014253 | Selt | Rattus norvegicus selenoprotein T (Sel | chr2 | 0.008064 | 6.222832 | 10.82734 | 10.7476 | 12.07344 | 7.86858 | 8.575504 | 9.291542 |
| NM_031068 | Actr3 | Rattus norvegicus ARP3 actin-related p | chr13 | 0.00612 | 3.3662906 | 13.71308 | 13.6647 | 13.63493 | 11.68669 | 12.05391 | 12.01866 |
| NM_017327 | Gnao | Rattus norvegicus guanine nucleotide b | chr19 | 0.046653 | 4.9857764 | 7.825426 | 7.51553 | 8.270235 | 4.484804 | 5.862042 | 6.310886 |
| NM_001044241 | RGD13098 | Rattus norvegicus similar to RIKEN cD | chr1 | 0.048723 | 4.409904 | 5.513341 | 4.57475 | 6.412965 | 3.398812 | 3.270415 | 3.409589 |
| NM_001108339 | Gatc | glutamyl-tRNA(Gln) amidotransferase | chr12 | 0.019617 | 3.5440264 | 7.461635 | 7.19239 | 7.050006 | 5.643586 | 5.812758 | 4.771521 |
| NM_139084 | Magi3 | Rattus norvegicus membrane associate | chr2 | 0.016938 | 2.183166 | 10.38978 | 10.2778 | 10.28457 | 8.998569 | 9.400261 | 9.174078 |
| NM_001108982 | Cfl2 | cofilin 2, muscle" | chr6 | 0.020223 | 3.077067 | 9.193144 | 9.59186 | 8.676378 | 7.105764 | 8.245097 | 7.245848 |
| NM_001024886 | Dcun1d3 | Rattus norvegicus DCN1, defective in | chr1 | 0.030698 | 2.044808 | 6.870142 | 6.89634 | 6.522625 | 6.112186 | 5.942946 | 5.13808 |
| NM_031762 | Cdkn1b | Rattus norvegicus cyclin-dependent kin | chr4 | 0.012626 | 2.829263 | 12.18939 | 12.7244 | 12.15038 | 10.87064 | 10.88382 | 10.80845 |
| NM_001106470 | RGD13048 | hypothetical protein LOC295428 | chr2 | 0.00214 | 2.3576937 | 10.85922 | 10.9937 | 10.92099 | 9.52199 | 9.855058 | 9.684723 |
| NM_001108034 | Mpp5 | membrane protein, palmitoylated 5 (M | chr6 | 0.021361 | 2.4125714 | 6.784769 | 6.74507 | 6.963661 | 5.523157 | 5.796829 | 5.361801 |
| NM_017038 | Ppm1a | Rattus norvegicus protein phosphatase | chr6 | 0.047721 | 4.365298 | 10.20217 | 9.58231 | 10.54832 | 8.028202 | 8.313757 | 7.612603 |
| NM_012986 | Nedd4 | neural precursor cell expressed, develo | chr8 | 0.019489 | 3.6782749 | 15.16542 | 15.2516 | 15.05083 | 13.15055 | 13.88632 | 12.79386 |
| NM_001107261 | Supt16h | suppressor of Ty 16 homolog | chr15 | 0.007657 | 2.3540199 | 11.97691 | 11.7203 | 11.99519 | 10.53373 | 10.64389 | 10.80943 |
| NM_001013182 | RGD13051 | Rattus norvegicus similar to HN1-like | chr10 | 0.025139 | 3.260328 | 11.92269 | 11.9429 | 12.29888 | 9.775053 | 10.74352 | 10.5308 |

| | | | | | | | | | | | |
|---|---|---|---|---|---|---|---|---|---|---|---|
| NM_001107197 | Igsf9 | immunoglobulin superfamily, member | chr13 | 0.007926 | 6.2477875 | 7.599751 | 7.01051 | 7.058875 | 4.498996 | 4.489924 | 4.750178 |
| NM_012517 | Cacna1c | Rattus norvegicus calcium channel, vo | chr4 | 0.025514 | 2.2930996 | 10.16898 | 10.2094 | 9.789537 | 8.629231 | 9.021743 | 8.925074 |
| NM_053714 | Ank | Rattus norvegicus progressive ankylos | chr2 | 0.007803 | 3.934255 | 12.03549 | 12.0961 | 12.7027 | 10.28173 | 10.24427 | 10.38003 |
| NM_013006 | Lypla1 | Rattus norvegicus lysophospholipase 1 | chr5 | 0.032711 | 2.3067994 | 11.94952 | 12.6571 | 11.81908 | 10.46575 | 11.28665 | 11.05563 |
| NM_001047892 | LOC31716 | Rattus norvegicus similar to Set alpha | chr18 | 0.013975 | 4.6928296 | 6.307856 | 5.6948 | 6.357148 | 3.633972 | 3.942112 | 4.09235 |
| NM_017220 | Pts | Rattus norvegicus 6-pyruvoyl-tetrahyd | chr8 | 0.025378 | 5.5886436 | 8.053043 | 7.60938 | 8.690538 | 4.840967 | 5.789124 | 6.275373 |
| NM_133319 | Taf2 | TAF2 RNA polymerase II, TATA box | chr7 | 0.023164 | 2.060842 | 11.0006 | 11.1998 | 11.06868 | 10.01419 | 10.40366 | 9.721494 |
| NM_030829 | Gprk5 | Rattus norvegicus G protein-coupled r | chr1 | 0.006707 | 2.07349 | 9.75181 | 9.94786 | 9.684468 | 8.531183 | 8.945646 | 8.751122 |
| NM_001106172 | Nod2 | nucleotide-binding oligomerization do | chr19 | 0.025286 | 2.2348716 | 5.056175 | 5.08153 | 4.43844 | 3.998887 | 3.556562 | 3.540125 |
| NM_139040 | RGD6210 | Rattus norvegicus similar to RIKEN cI | chr19 | 0.003676 | 5.0597396 | 9.022933 | 9.10759 | 9.053658 | 6.737519 | 6.983604 | 6.445872 |
| NM_001017448 | RGD1311 | Rattus norvegicus similar to RIKEN cI | chr20 | 0.018411 | 10.128335 | 6.557157 | 6.37023 | 7.003002 | 2.817606 | 3.946592 | 3.145217 |
| NM_001009688 | Thumpd1 | Rattus norvegicus THUMP domain co | chr1 | 0.038559 | 3.309436 | 11.45651 | 11.4187 | 11.46794 | 9.705224 | 10.30892 | 9.149211 |
| NM_001106726 | RGD1310 | hypothetical protein LOC299002 | chr6 | 0.042079 | 3.6696131 | 5.663314 | 5.60782 | 6.4557 | 4.396996 | 3.869518 | 3.833432 |
| NM_017206 | Slc6a6 | Rattus norvegicus solute carrier family | chr4 | 0.004902 | 4.60961 | 7.352073 | 7.32069 | 8.151993 | 4.895424 | 5.398176 | 5.917224 |
| NM_001134564 | RGD1564 | hypothetical protein LOC315843 | chr8 | 0.018067 | 3.8405242 | 6.530395 | 7.58991 | 6.744726 | 4.724107 | 5.13808 | 5.178938 |
| NM_001047916 | Syncrip | Rattus norvegicus synaptotagmin bindi | chr8 | 0.018621 | 8.481107 | 6.602971 | 6.07335 | 6.602564 | 3.022314 | 3.838801 | 3.16501 |
| NM_001126089 | Cdc42se2 | CDC42 small effector 2 | chr10 | 0.000504 | 2.0583885 | 11.58651 | 11.4911 | 11.64463 | 10.56151 | 10.40341 | 10.63274 |
| NM_001008289 | Sbds | Rattus norvegicus Shwachman-Bodian | chr12 | 0.036078 | 5.799171 | 11.74921 | 11.6739 | 11.68564 | 8.43877 | 10.05972 | 9.002695 |
| NM_019133 | Syn1 | Rattus norvegicus synapsin I (Syn1), n | chrX | 0.000597 | 4.9341307 | 7.802013 | 8.1851 | 7.75458 | 5.609585 | 5.807937 | 5.415785 |
| NM_001009631 | Tmco1 | Rattus norvegicus transmembrane and | chr13 | 0.040902 | 2.5962763 | 11.38341 | 11.1483 | 12.20011 | 9.850511 | 10.32888 | 10.42314 |
| NM_133317 | Tob1 | Rattus norvegicus transducer of ErbB- | chr10 | 0.030421 | 6.478122 | 10.70655 | 10.9634 | 11.04856 | 7.24187 | 9.153375 | 8.236517 |
| NM_001134856 | Cdc14a | CDC14 cell division cycle 14 homolog | chr2 | 0.008471 | 2.0837266 | 11.69263 | 12.1604 | 11.78656 | 10.66155 | 11.25537 | 10.54517 |
| NM_001100815 | Gtpbp10 | GTP-binding protein 10 | chr4 | 0.021927 | 8.570526 | 8.139533 | 8.44365 | 8.26193 | 4.745595 | 6.258499 | 4.542863 |
| NM_001108261 | RGD1305 | hypothetical protein LOC360461 | chr10 | 0.007823 | 11.109496 | 9.396022 | 9.0077 | 9.460384 | 5.672637 | 6.148524 | 5.62178 |
| NM_017268 | Hmgcs1 | Rattus norvegicus 3-hydroxy-3-methyl | chr2 | 0.033775 | 4.253696 | 10.09687 | 9.589 | 10.35434 | 8.565396 | 7.703968 | 7.504698 |
| NM_001037097 | Pomt2 | Rattus norvegicus protein-O-mannosyl | hr6_randon | 0.025746 | 3.1706157 | 7.457831 | 7.68145 | 7.387602 | 5.486299 | 6.559991 | 5.486299 |
| NM_022387 | Pafah1b2 | Rattus norvegicus platelet-activating fa | chr8 | 0.005691 | 2.3640375 | 11.00759 | 11.2428 | 11.38178 | 9.886295 | 10.06703 | 9.955103 |
| NM_212508 | Nrm | Rattus norvegicus nurim (nuclear enve | chr20 | 0.010215 | 7.8097224 | 6.551503 | 5.98737 | 6.565125 | 3.097059 | 3.573568 | 3.537556 |
| NM_001033663 | Araf | v-raf murine sarcoma 3611 viral oncog | chrX | 0.003427 | 2.1310115 | 11.65186 | 11.8333 | 12.02877 | 10.6674 | 10.62732 | 10.94463 |
| NM_001107378 | Znf608 | zinc finger protein 608 | chr18 | 0.007621 | 4.1311107 | 7.168492 | 7.78033 | 7.090886 | 5.425328 | 5.415278 | 5.059512 |
| NM_053973 | Rraga | Rattus norvegicus Ras-related GTP bir | chr5 | 0.012815 | 3.5943022 | 13.10564 | 12.9087 | 13.1233 | 10.89906 | 11.4328 | 11.26865 |
| NM_021767 | Nrxn1 | Rattus norvegicus neurexin 1 (Nrxn1), | chr6 | 0.025648 | 6.462133 | 7.999837 | 8.52962 | 9.425938 | 4.612605 | 6.651389 | 6.615365 |
| NM_172008 | Canx | Rattus norvegicus calnexin (Canx), mR | chr10 | 0.005908 | 3.2271016 | 5.29878 | 5.84558 | 6.134721 | 3.832857 | 3.927629 | 4.447879 |

| | | | | | | | | | | | |
|---|---|---|---|---|---|---|---|---|---|---|---|
| NM_001014221 | LOC36333 | Rattus norvegicus similar to RIKEN cI | chrUn | 0.008996 | 2.1588411 | 11.67864 | 11.0226 | 10.57165 | 10.68278 | 9.700493 | 9.558807 |
| NM_173097 | Reg3g | Rattus norvegicus regenerating islet-de | chr4 | 0.027564 | 19.798466 | 12.47178 | 8.84685 | 10.07519 | 6.769297 | 5.611517 | 6.091048 |
| NM_012825 | Aqp4 | Rattus norvegicus aquaporin 4 (Aqp4), | chr18 | 0.011044 | 2.522217 | 7.853818 | 8.07907 | 6.884209 | 6.238188 | 6.856165 | 5.718663 |
| NM_001105732 | Sec23a | SEC23 homolog A | chr6 | 0.009339 | 4.913643 | 7.752923 | 7.87294 | 8.665154 | 5.241464 | 6.02309 | 6.136084 |
| NM_001047886 | Ilf2 | Rattus norvegicus interleukin enhancer | chr2 | 0.025137 | 27.943642 | 7.65892 | 6.22983 | 8.908876 | 2.817606 | 2.788281 | 2.77839 |
| NM_001079893 | Dusp14_pr | Rattus norvegicus dual specificity phos | chr10 | 0.029151 | 3.3210652 | 7.134895 | 5.85203 | 6.179269 | 5.198229 | 4.715478 | 4.057548 |
| NM_001107900 | Cpa6 | carboxypeptidase A6 | chr5 | 0.030995 | 2.1581285 | 4.559844 | 6.17791 | 4.690358 | 3.175517 | 4.953325 | 3.969928 |
| NM_001106425 | Armc1 | armadillo repeat containing 1 | chr2 | 0.007765 | 8.902115 | 9.371633 | 10.0084 | 9.780608 | 6.732345 | 6.785655 | 6.180177 |
| NM_001013895 | Prkd2 | Rattus norvegicus protein kinase D2 (F | chr1 | 0.040469 | 2.657598 | 9.166003 | 8.7647 | 9.139247 | 7.385696 | 7.932249 | 7.521634 |
| NM_181478 | Rdh10 | Rattus norvegicus retinol dehydrogena | chr5 | 0.022144 | 2.51428 | 8.851442 | 9.0671 | 8.77069 | 7.317394 | 8.139533 | 7.24187 |
| NM_001012087 | 7-Mar | Rattus norvegicus membrane-associate | chr3 | 0.00199 | 28.492098 | 9.894786 | 10.4194 | 10.11638 | 5.456252 | 5.236911 | 5.239925 |
| NM_017309 | Ppp3r1 | Rattus norvegicus protein phosphatase | chr14 | 0.044682 | 14.696012 | 10.91298 | 10.5595 | 11.18694 | 7.694879 | 7.706372 | 5.626124 |
| NM_001024759 | Tmem5 | Rattus norvegicus transmembrane prot | chr7 | 0.040336 | 2.2120848 | 9.424246 | 9.89247 | 9.431623 | 8.344219 | 9.121433 | 7.846462 |
| NM_022247 | Pdcl | Rattus norvegicus phosducin-like (Pdc | chr3 | 0.016282 | 4.524902 | 9.809015 | 10.1764 | 10.02559 | 8.144878 | 7.940408 | 7.392045 |
| NM_138528 | Ero1l | Rattus norvegicus ERO1-like (S. cerev | chr15 | 0.001041 | 12.1441555 | 6.52708 | 6.95419 | 8.616995 | 3.122407 | 3.359662 | 4.809626 |
| NM_019210 | Pak3 | Rattus norvegicus p21 (CDKN1A)-act | chrX | 0.029987 | 2.6386287 | 7.993611 | 8.40021 | 8.454012 | 6.127192 | 7.379417 | 7.141859 |
| NM_001107897 | Gdap1 | ganglioside-induced | chr5 | 0.005398 | 2.0928402 | 12.21685 | 12.5093 | 12.16174 | 11.15156 | 11.30756 | 11.23234 |
| NM_001107921 | Casp8ap2 | caspase 8 associated protein 2 | chr5 | 0.043815 | 2.5956464 | 9.274572 | 9.7177 | 9.013869 | 7.480373 | 8.183004 | 8.214479 |
| NM_001013077 | Plekha3 | Rattus norvegicus pleckstrin homology | chr3 | 0.03185 | 2.4337585 | 8.967615 | 9.79391 | 8.728417 | 7.746244 | 8.076939 | 7.817204 |
| NM_001039378 | Trappc1 | Rattus norvegicus trafficking protein p | chr10 | 0.000189 | 2.191722 | 12.23405 | 12.285 | 11.76937 | 11.09207 | 11.18345 | 10.61669 |
| NM_001014022 | Btbd10 | K+ channel tetramerization protein | chr1 | 0.006177 | 2.63755 | 11.17091 | 11.4335 | 11.37676 | 9.677821 | 10.25444 | 9.851284 |
