# Supplementary material for: Role of microRNAs in the age-associated decline of pancreatic beta cell function in rat islets
Source: Diabetologia. 2015 Oct 16;59(1):161–9. doi: 10.1007/s00125-015-3783-5 (PMC4670458; doi:10.1007/s00125-015-3783-5)
Supplement: Supplementary file 8 — (PDF 1457 kb) [file 125_2015_3783_MOESM8_ESM.pdf]

ESM Table 2

Analysis of the down-regulated mRNAs in the islets of 12 month-old rats

Two µg of RNA isolated from the islets of three 3 month-old and three 12 month-old rats were analyzed by microarray.
The signal intensities were quantile normalized and expressed on a Log2 scale.
Column B - E - Probe annotations (SeqID, Gene Symbol, description, chromosome)
Column F - P-value calculated from paired t-test
Column G - FCA absolute, an absolute fold change calculated between two groups
Column H,I - Normalized Intensity for each group (log2 transformed)
Column J,O - Normalized Intensity for each sample (log 2 transformed)
Fold Change cut off 2.0, p-value cut-off 0.5

						Normalized Intensity					
SeqID	Gene Symbol	Description	chromo-some	p-value	FC Absolute	12 month	12 month	12 month	3 month	3 month	3 month
NM_031645	Ramp1	Rattus norvegicus receptor (calcitonin	chr9	0.02212969	2.9301913	9.17365	7.628853	8.175745	10.26534	9.326156	10.039736
NM_133583	Ndrg2	Rattus norvegicus N-myc downstream	chr15	0.01019273	2.4824648	11.069812	10.255045	10.560946	12.530565	11.684126	11.60643
NM_0011093:	Cbln3	cerebellin 3 precursor	chr15	0.04470981	3.8176754	5.4076653	5.4684744	6.153372	7.3258405	8.140943	7.3608103
NM_0011082'	Bcl6b	B-cell CLL/lymphoma 6, member B (z	chr10	0.0212292	2.6852944	9.133327	8.680256	8.566919	10.752488	9.683753	10.219502
NM_0011137·	Rnf26	ring finger protein 26	chr8	0.04917873	2.030671	10.928369	11.167817	10.79933	12.03371	11.746794	12.180882
NM_053741	Rap2a	Rattus norvegicus RAS related protein	chr15	0.01252942	3.7114968	10.012714	10.24117	10.392804	11.519622	12.164773	12.638294
NM_0011095:	LOC689927	hypothetical protein LOC689927	chr20	0.02681877	2.1654615	7.858603	8.666811	7.8593073	9.325956	9.500982	8.901804
NM_001166275			chr20	0.03647004	2.556574	9.564713	9.575809	9.491161	11.355867	10.448885	10.889567
NM_053307	Msra	Rattus norvegicus methionine sulfoxid	chr15	0.0241373	2.0367043	10.533049	10.326903	10.563963	11.454983	11.671664	11.375977
NM_0011150:	RGD156531	hypothetical protein LOC315091	chr7	0.01531918	3.0606613	5.4879904	5.9575605	6.760291	6.8444333	7.9699416	8.232996
NM_053904	Oplah	Rattus norvegicus 5-oxoprolinase (AT	chr7	0.02038884	2.518631	10.566814	9.844669	10.215506	11.869386	11.526055	11.229466
NM_0011093:	Capns2	calpain, small subunit 2"	chr19	0.00208098	3.256042	7.269436	7.313903	7.7581024	8.832106	9.029193	9.589502
NM_031010	Alox15	Rattus norvegicus arachidonate 15-lip	chr10	0.02675867	2.5229983	6.965504	6.9836044	6.841825	8.72732	8.217281	7.851751
NM_031644	Ptgds2	Rattus norvegicus prostaglandin D2 sy	chr4	0.02582266	3.6247118	5.899621	4.6915913	3.2851005	7.7402143	6.030936	5.678761
NM_0010174	RGD131145	Rattus norvegicus similar to hypotheti	chr1	0.00887289	3.068193	6.453973	7.877406	7.240291	7.9656096	9.298307	9.159924
NM_021588	Mb	Rattus norvegicus myoglobin (Mb), m	chr7	0.02508447	5.093149	3.1512532	4.4381514	3.7239268	6.2404523	6.275598	5.842954
NM_0010088:	RT1-M6-1	RT1 class I, M6, gene 1"	chr20	0.03450153	2.0868008	3.0577817	3.655763	3.10598	4.261447	4.317689	4.424268
NM_031783	Nefl	Rattus norvegicus neurofilament, light	chr15	0.03321449	2.481675	8.189296	8.434178	6.903185	9.984005	9.574641	7.901955

NM_019128	Inexa	Rattus norvegicus internexin, alpha (In	chr1	0.04672956	2.1406057	5.3718214	5.31864	5.5854173	6.1347213	6.8963413	6.538874
NM_0010147	Gm52_predi	Rattus norvegicus envelope glycoprote	chr12	0.000903	2.2281861	3.148586	2.9657092	3.0488672	4.323481	4.1699743	4.1373158
NM_0010002	Olr237_pred	Rattus norvegicus olfactory receptor 2	chr1	0.04162434	3.391029	7.363387	8.24318	7.511731	9.79933	9.399	9.205137
NM_0011060	Abca13	ATP-binding cassette, sub-family A (A	chr14	0.04456293	3.4977634	3.104823	4.4033737	3.3111165	5.1362762	5.4421067	5.6602283
NM_0010001	Olr56_predi	Rattus norvegicus olfactory receptor 5	chr1	0.04241655	2.6933253	6.072437	7.303214	6.628951	8.109201	8.39992	7.7836456
NM_0010000	Olr1608_pre	Rattus norvegicus olfactory receptor 1	chr15	0.02417151	3.3834035	6.4419994	7.5628457	6.397281	8.620105	8.794386	8.263061
NM_0010336	Bcl2l1	Bcl2-like 1 isoform 1	chr3	0.01179739	2.6077876	6.895183	7.1816	6.8763094	8.3405	8.790018	7.9710536
NM_0010140	LOC308650	Rattus norvegicus similar to Murine h	chrX	0.03994559	3.4268682	5.2831492	5.3092937	5.555466	6.8985863	7.7855854	6.7944107
NM_133577	P2ry14	Rattus norvegicus purinergic receptor	chr2	0.04115389	2.7881536	9.199197	8.764403	8.697477	10.071042	10.652618	10.375346
NM_0010995	Vom2r71	vomeronasal 2 receptor, 71"	chr14	0.04334999	2.620331	6.490964	8.460217	6.9545245	8.357946	9.298786	8.418222
NM_0010250	Klhl38	kelch-like 38	chr7	0.04913714	2.3645604	6.4086933	6.604034	5.4732037	7.4048924	7.5210834	7.2846723
NM_019236	Hes2	Rattus norvegicus hairy and enhancer	chr5	0.04164477	4.062554	5.1051197	5.7074056	5.3215275	7.978251	7.2602415	6.96272
NM_0011150	Gltpd2	glycolipid transfer protein domain con	chr10	0.0095496	2.795343	3.1172996	2.9418726	2.9676435	4.7333155	4.5834765	4.159099
NM_0011059	RGD156246	hypothetical protein LOC289236	chr13	0.01129811	3.1337066	5.9888573	6.334289	6.4877386	7.394038	7.8811336	8.479326
NM_0010377	Trpm1	Rattus norvegicus transient receptor p	chr1	0.03060449	2.4760609	6.734559	7.1457186	7.2239647	7.574399	8.673882	8.780104
NM_175837	Cyp4a22	Rattus norvegicus cytochrome P450, f	chr5	0.02971795	2.112502	12.726661	13.171459	12.621169	14.183374	14.101242	13.471531
NM_053954	Kcns1	Rattus norvegicus K+ voltage-gated cl	chr3	0.03088713	2.3841362	6.0827394	6.9675198	6.5877085	7.78727	7.990262	7.6208334
NM_0010005	Olr37	Rattus norvegicus olfactory receptor 3	chr1	0.0084448	3.6319785	3.0357454	3.096769	3.1581151	5.023434	4.617006	5.2324557
NM_031680	P2ry4	Rattus norvegicus pyrimidinergic rece	chrX	0.00879589	2.105208	5.218111	5.8419967	5.293386	6.432176	6.7189426	6.424263
NM_139326	Pomc	Rattus norvegicus pro-opiomelanocort	chr6	0.00525597	2.1184313	10.94959	10.873892	10.921536	12.178942	11.832959	11.982104
NM_0011066	Rragd	Ras-related GTP binding D	chr5	0.00797337	2.6216538	8.968431	8.750059	9.034918	10.435406	10.308243	10.18119
NM_0011358	Saps1	SAPS domain family, member 1"	chr1	0.008967	4.055469	7.978251	8.924483	8.688933	10.35087	10.634104	10.6663
NM_053918	Cga	Rattus norvegicus glycoprotein hormo	chr5	0.04798378	2.921919	3.5396194	3.6094224	4.452808	5.623214	5.281613	5.337772
NM_0011089	Ferd3l	Fer3-like	chr6	0.00828306	3.5271208	8.646273	9.686288	9.126465	10.793358	11.293304	10.827836
NM_0011073	Nsd1	nuclear receptor binding SET domain	chr17	0.03916217	2.0688145	7.047468	7.131119	6.84528	8.512867	7.8874884	7.769924
NM_0011075	Tpcn2	two pore segment channel 2	chr1	0.01816188	2.7835515	8.531183	8.55892	8.176668	10.034024	9.674085	9.989442
NM_0010005	Olr710_pred	Rattus norvegicus olfactory receptor 7	chr3	0.02037919	2.1908507	5.4207134	5.966004	5.977517	6.83116	7.107584	6.8199644
NM_053869	Phox2a	Rattus norvegicus paired-like homeob	chr1	0.03264421	2.7847514	6.0388927	5.9592204	5.59201	7.9010983	7.581746	6.5399246
NM_0011355	LOC690806	hypothetical protein LOC690806	chr17	0.04421532	2.007751	7.4270654	7.3679185	7.2692113	7.999837	8.645481	8.435618
NM_0010130	Defcr4	Rattus norvegicus defensin related cry	chr16	0.01617818	2.4287312	4.8679795	5.301805	5.304431	6.4106402	6.277996	6.626188
NM_0011083	Gga3	golgi associated, gamma adaptin ear c	chr10	0.00428828	2.1917133	12.158854	12.40547	12.543588	13.224932	13.455071	13.824087
NM_0011346	RGD156097	hypothetical protein LOC500693	chr6	0.04295553	2.1818173	4.6969876	5.114274	4.285683	6.160331	5.772875	5.54033
NM_0010995	Vom2r48	vomeronasal 2 receptor, 48"	chr4	0.01094961	3.101634	2.9244432	3.0220485	2.8801699	4.7276936	4.8294454	4.168609

NM_0010142	LOC365592	Rattus norvegicus similar to CG10585	chr20	0.01674506	2.156632	10.080255	10.123637	10.11871	11.408281	10.957586	11.283074
NM_0010001	Olr92_predi	Rattus norvegicus olfactory receptor 9	chr1	0.02828368	2.5599935	6.080806	6.8127885	6.870484	7.84731	7.7723536	8.212835
NM_173317	LOC286983	Rattus norvegicus putative pheromone	chr1	0.04111739	2.9710264	2.9722908	2.8591118	2.836575	5.083263	3.8351443	4.462456
NM_0011068	Spink8	serine peptidase inhibitor, Kazal type 8	chr8	0.01959877	2.543205	8.4074745	7.940864	7.611873	9.509956	9.154389	9.335812
NM_0010373	Asb12	Rattus norvegicus ankyrin repeat and S	chrX	0.000166	2.5527987	3.2089925	3.0674357	3.045683	4.554057	4.393491	4.4308023
NM_017249	Mbc2	Rattus norvegicus membrane bound C	chr7	0.04267844	2.7358773	7.7549295	8.748272	9.048834	9.584688	9.585682	10.737675
NM_030993	Ddn	Rattus norvegicus dendrin (Ddn), mRN	chr7	0.01328983	6.9933305	5.6455703	6.02929	5.6675625	8.987086	8.24318	8.530096
NM_0011074	Cdh5	cadherin 5	chr19	0.04723086	4.189015	3.1314614	2.9437723	3.998381	4.449875	5.8651524	5.95842
NM_0010001	Olr140	olfactory receptor Olr140	chr1	0.03142842	3.5428054	3.6395118	3.6078768	2.8492799	4.9740705	5.291776	5.305499
NM_001163921			chr4	0.02674443	2.5534444	9.179913	9.8920555	10.352448	10.803371	10.796306	11.882071
NM_144741	Retn	Rattus norvegicus resistin (Retn), mRN	chr12	0.02093033	3.1206188	3.801473	2.8274217	2.8071926	5.739058	3.9910378	4.6314874
NM_080773	Chrm1	Rattus norvegicus cholinergic receptor	chr1	0.02652474	2.281809	3.6108627	3.8121285	3.8789601	4.770941	4.6757603	5.4257846
NM_0010004	Olr1341_pre	Rattus norvegicus olfactory receptor 1	chr8	0.03613259	3.5812137	2.888843	2.8196244	2.8043761	4.8098793	4.000745	5.223566
NM_0011137	Pabpnl1	poly(A)binding protein nuclear-like 1	chr19	0.0165017	2.1223574	8.925074	8.774442	9.007698	9.896663	9.693394	10.374162
NM_138542	Rhov	Rattus norvegicus ras homolog gene fa	chr3	0.01713705	2.1222382	10.220801	9.77627	10.084879	11.397264	11.053034	10.888409
NM_022627	Prkab2	Rattus norvegicus protein kinase, AMI	chr2	0.02596705	3.4794335	3.3666651	2.8209996	2.8068538	5.62466	4.067575	4.6988397
NM_0011066	Mul1	mitochondrial ubiquitin ligase activato	chr5	0.0147649	4.444383	8.616603	9.195894	8.376474	11.122806	10.830678	10.691435
NM_0011090	Praf2	PRA1 domain family, member 2"	chrX	0.00663461	2.1270092	10.146575	10.162439	9.950413	11.224524	11.10261	11.198771
NM_0010994	Vom2r17	vomeronasal 2 receptor 17	chr1	0.01120767	2.0790884	5.0736613	5.7062516	5.1767416	6.071338	6.602704	6.4504666
NM_0011090	Col6a3	procollagen, type VI, alpha 3"	chr9	0.02317635	4.3174767	6.5599914	7.070961	6.4430704	9.273588	8.66119	8.469811
NM_030834	Slc16a3	Rattus norvegicus solute carrier family	chr10	0.0285072	2.4092646	9.29237	9.722409	10.346416	10.994234	10.828733	11.344005
NM_0010070	Scospondin	Rattus norvegicus subcommissural org	chr4	0.0028094	2.6238167	7.4489346	7.5832653	7.1748495	8.903572	8.827609	8.650867
NM_022714	Crhr2	Rattus norvegicus corticotropin releasi	chr4	0.04050594	2.2299938	3.7376268	5.1928425	5.5623045	5.3475895	6.262247	6.354056
NM_0010005	Olr1433_pre	Rattus norvegicus olfactory receptor 1	chr10	0.02782773	2.267889	3.0629766	3.0493588	3.22678	4.6395516	4.099474	4.1441393
NM_0011071	Col8a1	collagen, type VIII, alpha 1"	chr11	0.00866735	2.1011143	10.595554	10.816329	11.832335	11.629532	12.076923	12.751229
NM_019136	Avpr2	Rattus norvegicus arginine vasopressin	chrX	0.0217374	4.76909	5.480772	5.978501	6.097044	8.10405	7.5576205	8.655789
NM_173149	Havcr1	Rattus norvegicus kidney injury molec	chr10	0.03666707	2.7251318	3.7792885	2.8202696	2.8052413	4.888279	4.0376897	4.817809
NM_0011070	Slc7a4	solute carrier family 7 (cationic amino	chr11	0.04139284	2.4658449	10.43606	10.697016	11.062036	12.124844	11.470764	12.505751
NM_0011062	Map4k1	mitogen activated protein kinase kinas	chr1	0.02477919	2.411237	10.079988	8.864538	9.417774	10.957586	10.234942	10.979094
NM_0010825	Trim21_pre	Rattus norvegicus tripartite motif prote	chr1	0.02658219	2.758351	8.231651	8.654059	9.647749	9.210476	10.321209	11.393196
NM_145781	Timm13	Rattus norvegicus translocase of inner	chr7	0.04289228	2.0693932	8.899929	8.95719	9.301237	10.383219	9.889329	10.033427
NM_0010088	Krt33b	keratin 33B	chr10	0.03344815	4.874952	6.181541	6.456428	7.142996	7.8499427	8.534445	10.252738
NM_0011276	Sh3bgr	SH3 domain binding glutamic acid-ric	chr11	0.02451728	2.1085706	10.450799	10.580073	10.113081	11.561632	11.343181	11.467936

NM_0011009	Pard6g	par-6 partitioning defective 6 homolog	chr18	0.04191818	2.9272177	5.289357	5.7057085	6.038056	7.4941206	6.9345837	7.2530065
NM_0010479	LOC498152	Rattus norvegicus hypothetical protein	chr12	0.0413501	2.460814	7.790258	8.075281	7.8861837	9.490841	8.853948	9.304344
NM_0011089	RGD156155	hypothetical protein LOC365547	chr20	0.04278163	2.0010896	9.500982	9.761145	9.409206	10.720681	10.334118	10.618893
NM_0011095	Rab20	RAB20, member RAS oncogene famil	chr16	0.02230008	3.1686838	2.8007772	2.7964146	2.7851596	4.9700093	4.215742	4.1882505
NM_0011090	Myoz1	myozenin 1	chr15	0.0125908	2.2327785	6.92304	6.424263	6.3510976	8.242107	7.3228655	7.6099486
NM_013109	Otx1	Rattus norvegicus orthodenticle homo	chr14	0.01432452	6.3603344	4.4248466	5.223566	5.297744	7.09137	7.334629	8.527466
NM_0011272	Fam84a	family with sequence similarity 84, me	chr6	0.01096798	2.3367147	8.475394	8.82515	8.86053	9.844669	9.79167	10.198183
NM_019287	Apob	Rattus norvegicus apolipoprotein B (A	chr6	0.000118	2.5281453	9.190692	8.98852	8.297307	10.511805	10.355496	9.62345
NM_024364	Hr	Rattus norvegicus hairless homolog (m	chr15	0.0048365	2.9824264	7.1121545	7.898123	8.319545	8.894906	9.305031	9.859346
NM_017148	Csrp1	Rattus norvegicus cysteine and glycine	chr13	0.000157	2.8752747	13.589726	12.705269	13.111293	15.104414	14.201307	14.671664
NM_0010250	LOC500102	Rattus norvegicus hypothetical protein	chr4	0.0282584	6.475957	3.6855774	2.8016062	2.804205	6.7485723	6.0485296	4.5795665
NM_173330	Hit39	Rattus norvegicus zinc finger protein I	chr1	0.0295283	2.0286705	5.9639673	6.829321	6.7959237	7.34149	7.7019134	7.607412
NM_020104	Myl1	Rattus norvegicus myosin, light polype	chr9	0.00735255	3.6717849	8.12177	7.295958	7.3342867	9.674686	9.337932	9.368838
NM_0010123	Ccl9	Rattus norvegicus chemokine (C-C mo	chr10	0.0476258	4.111549	7.0263877	6.2849503	7.4160447	9.1255045	7.496732	10.224191
NM_199402	Spata20	Rattus norvegicus spermatogenesis ass	chr10	0.04415126	2.2122092	7.9671345	8.585985	8.110317	9.093155	9.310229	9.696515
NM_0011095	Pmp2	peripheral myelin protein 2	chr2	0.02168884	2.145743	3.8977349	3.214731	2.7977796	4.9582424	4.6201143	3.6363199
NM_0011059	Ppox	protoporphyrinogen oxidase	chr13	0.02533018	2.0344894	11.454983	11.307022	11.833849	12.681691	12.459378	12.528788
NM_053296	Glrb	Rattus norvegicus glycine receptor, be	chr2	0.00845313	2.1053164	6.7258615	7.203126	7.1637425	7.988513	8.128569	8.19776
NM_0010139	LOC294513	Rattus norvegicus similar to DnaJ (Hs	chr20	0.03174557	2.2182906	9.783195	10.210316	9.971557	11.000598	10.967148	11.445668
NM_0010242	Ptprcap	protein tyrosine phosphatase, receptor	chr1	0.04627845	2.8232863	9.305234	8.327798	7.96205	10.392804	9.573616	10.120789
NM_0011095	Grpel2	GrpE-like 2, mitochondrial"	chr18	0.000157	2.7125044	3.3150747	2.879342	2.9500556	4.786936	4.3168497	4.3595634
NM_0010028	Btnl3	Rattus norvegicus butyrophilin-like 3 (	chr20	0.01212486	2.7593505	3.2039123	2.7811809	2.7710047	4.894652	3.9298396	4.324593
NM_0010003	Olr416_pred	Rattus norvegicus olfactory receptor 4	chr3	0.04466922	2.3289802	4.7182183	4.4554234	4.1818132	5.4263406	5.7994714	5.7887383
NM_012632	Prp15	Rattus norvegicus proline-rich protein	chr4	0.04966166	2.1844683	6.123974	6.0694747	6.000486	7.4011164	7.5549335	6.6197333
NM_0011033	Adat3	adenosine deaminase, tRNA-specific 3	chr7	0.02049173	2.241071	7.850872	8.149721	8.014552	9.352034	9.117335	9.038341
NM_0010375	Defb17	beta-defensin 17	chr9	0.02191598	3.1326947	2.7809107	3.4840045	2.80166	4.6366453	4.6374655	4.7346773
NM_053806	Kcnk6	Rattus norvegicus potassium channel,	chr1	0.0267079	2.2910662	8.934693	9.009577	9.323499	9.916985	10.604204	10.334637
NM_0011057	Tek	TEK tyrosine kinase, endothelial"	chr5	0.024263	2.1858838	11.447993	10.840576	10.531585	12.39576	11.791226	12.017823
NM_024140	Nrgn	Rattus norvegicus neurogranin (Nrgn),	chr8	0.000306	2.5779185	7.968779	7.228717	7.002469	9.345871	8.629784	8.322931
NM_0010010	Olr1138_pre	Rattus norvegicus olfactory receptor 1	chr8	0.01824992	2.156465	4.6915913	5.3100734	4.700758	5.6503353	6.264957	6.113137
NM_172041	Kcnk2	potassium channel, subfamily K, mem	chr13	0.04568984	2.493621	2.7825916	2.7792869	2.7683415	4.6649256	3.9455743	3.6744466
NM_0011007	E2f1	E2F transcription factor 1	chr3	0.00235725	2.7217784	8.658879	8.841654	8.919821	9.986142	10.277824	10.490036
NM_0011033	LOC691995	hypothetical protein LOC691995	chr10	0.0073253	2.2416859	9.169968	8.687707	8.563698	10.46892	9.913928	9.532278

NM_0011064	Igsf3	immunoglobulin superfamily, member	chr2	0.02277622	2.296091	2.9418726	2.8599067	2.8560126	4.3882027	3.699062	4.168067
NM_0011068	Adamts15	ADAM metallopeptidase with thromb	chr8	0.00972183	2.6750886	8.4025755	7.4882255	7.1364155	9.543524	9.084731	8.657723
NM_130403	Ppp1r14a	Rattus norvegicus protein phosphatase	chr1	0.0278752	2.2583442	10.663095	9.620708	10.24605	11.94285	11.078892	11.033906
NM_023023	Dpysl5	Rattus norvegicus dihydropyrimidinas	chr6	0.02448134	2.018152	11.395909	11.321613	11.180844	12.183972	12.24645	12.507045
NM_0011079	Tbc1d2	TBC1 domain family, member 2"	chr5	0.006758	2.294293	9.821865	9.876122	9.975301	10.924233	10.971907	11.371293
NM_012613	Npr1	Rattus norvegicus natriuretic peptide r	chr2	0.000637	2.398686	10.173203	9.419476	9.548574	11.374042	10.697587	10.856356
NM_017131	Casq2	Rattus norvegicus calsequestrin 2 (Cas	chr2	0.02175019	2.3920338	9.919675	9.090727	8.793666	10.920096	10.239292	10.419394
NM_031545	Nppb	Rattus norvegicus natriuretic peptide p	chr5	0.02115885	3.2549782	3.9339845	2.7916644	2.7859185	5.168031	4.8877935	4.5636864
NM_0011095	Gpr137	G protein-coupled receptor 137	chr1	0.00787155	2.1667602	11.387907	11.342053	11.615702	12.336332	12.634916	12.721035
NM_031100	Rpl10	Rattus norvegicus ribosomal protein L	chrX	0.04047697	3.3007014	8.123626	8.58068	8.28369	10.525658	9.770223	9.860434
NM_0011069	Foxo4	forkhead box O4	chrX	0.03305785	2.2316778	9.10542	9.30031	9.489367	9.995504	10.29915	11.074827
NM_0010349	C1qtnf6	Rattus norvegicus C1q and tumor necr	chr7	0.01600253	2.074074	10.07913	10.279651	10.104888	11.262959	11.062624	11.295487
NM_013090	Vamp1	Rattus norvegicus vesicle-associated n	chr4	0.0000536	2.1747918	12.090617	12.065664	11.895815	13.201272	13.180538	13.032919
NM_0011058	Rdm1	RAD52 motif 1	chr10	0.01659235	2.0018928	9.094359	8.91951	8.985323	10.304015	9.680184	10.01909
NM_012874	Ros1	Rattus norvegicus v-ros UR2 sarcoma	chr20	0.04758538	2.4704018	11.855784	11.170914	12.145421	12.952108	13.058369	13.075882
NM_0011262	RGD131026	hypothetical protein LOC314472	chr6	0.01553148	2.0309448	13.078463	13.187525	13.432274	14.101242	13.986114	14.677357
NM_012601	Mpg	Rattus norvegicus N-methylpurine-DN	chr10	0.00899741	2.5858881	9.444559	9.57838	9.141992	10.783435	10.739886	10.75359
NM_0011063	Cd248	CD248 molecule, endosialin"	chr1	0.01908734	2.1738071	10.099022	9.561015	9.496639	10.907354	10.80538	10.804617
NM_0010072	Os-9	Rattus norvegicus amplified in osteosa	chr7	0.00897899	2.299589	13.793116	13.574062	14.022643	15.09015	14.908012	14.995787
NM_0011076	Sprr3	small proline-rich protein 3	chr2	0.01202339	3.141612	4.1995335	4.372265	3.281734	5.877612	5.6948037	5.235633
NM_0011081	Tbx18	T-box18	chr8	0.00422057	2.2166364	7.5232	7.37744	7.059968	8.763054	8.377469	8.265204
NM_013174	Tgfb3	Rattus norvegicus transforming growtl	chr6	0.04648441	2.1538622	11.113262	10.42201	10.616686	11.747247	11.891156	11.834333
NM_031727	Limk1	Rattus norvegicus LIM motif-containi	chr12	0.0362703	2.9121077	9.859346	10.33775	9.9327755	11.669217	11.277139	11.809711
NM_0010120	Fkhl18	Rattus norvegicus forkhead-like 18 (D	chr3	0.00315788	2.6876154	9.656369	9.145079	9.268324	11.206009	10.598973	10.543774
NM_0010249	Trex1	Rattus norvegicus three prime repair e	chr8	0.0477699	2.091807	10.15573	10.141067	10.072542	11.420834	10.725122	11.417631
NM_031587	Pxmp2	Rattus norvegicus peroxisomal membr	chr12	0.0321843	2.0638752	10.649665	10.339924	10.080255	11.316474	11.516601	11.372834
NM_0011057	Rasgrf1	RAS protein-specific guanine	chr8	0.03520169	2.3020253	3.4102201	2.7776392	2.7926908	4.52958	3.6272984	4.4323835
NM_139113	Nr2f6	Rattus norvegicus nuclear receptor sub	chr16	0.02133711	2.0904164	11.554194	11.554194	11.765531	12.472285	12.448134	13.144871
NM_0011052	Mrvi1	MRV integration site 1 homolog isofo	chr1	0.01083893	2.758076	9.808561	9.214572	8.808646	11.254268	10.421565	10.546933
NM_133385	Ucn2	Rattus norvegicus urocortin 2 (Ucn2),	chr8	0.00023	2.9226136	3.472051	2.763986	3.5605497	4.976445	4.3491635	5.112755
NM_130824	Rasgrp4	Rattus norvegicus RAS guanyl releasi	chr1	0.01257322	2.5111864	5.2652006	4.6451936	4.3778596	6.715402	5.6745224	5.8834367
NM_013191	S100b	Rattus norvegicus S100 protein, beta p	chr20	0.04505778	2.6543648	8.747962	7.610977	7.352824	9.566442	9.151761	9.218662
NM_031663	Slc18a3	Rattus norvegicus solute carrier family	chr16	0.02454429	2.6326041	7.0918593	7.3557773	6.2037044	8.863409	8.356026	7.621377

NM_017026	Mbp	myelin basic protein isoform 5	chr18	0.03926319	3.9262788	7.3905997	7.73708	6.799166	9.208141	9.103269	9.534925
NM_0010242	Spib	Spi-B transcription factor (Spi-1/PU.1	chr1	0.01135295	2.7373886	6.95266	6.613896	6.298784	8.204179	7.960594	8.058969
NM_0010316	Abca17	Rattus norvegicus ATP-binding casset	chr10	0.03647195	5.8157167	4.2690363	5.1789384	2.7680306	6.6800365	6.9266386	6.2292023
NM_0011344	RGD131178	hypothetical protein LOC287871	chr10	0.01953548	2.1685686	12.126345	12.442948	12.119797	13.4308	13.244791	13.363732
NM_183053	Rhcg	Rattus norvegicus Rhesus blood group	chr1	0.0088276	2.223516	3.7880335	3.464786	2.747579	4.7725997	4.5816393	4.104688
NM_130399	Ada	Rattus norvegicus adenosine deaminas	chr3	0.01400428	2.4703512	10.386696	9.887968	10.386969	11.854427	11.341648	11.379703
NM_139341	Slc15a3	Rattus norvegicus solute carrier family	chr1	0.01288531	3.0978916	8.826508	8.185808	8.218982	10.778849	9.490486	9.855822
NM_0011066	Leprel2	leprecan-like 2	chr4	0.02284651	2.3406506	12.81063	12.061901	12.860472	13.859558	13.666005	13.888167
NM_0010120	Ppp1r3c	Rattus norvegicus protein phosphatase	chr1	0.03045324	2.0797853	9.603048	9.2999525	8.7987795	11.005044	10.05224	9.813798
NM_0011091	Spata2L	spermatogenesis associated 2-like	chr19	0.00857597	2.6016192	9.6396055	9.605146	9.966362	11.268201	10.914931	11.166212
NM_0011272	Zc3hc1	zinc finger, C3HC-type containing 1"	chr4	0.00268552	2.048068	10.198183	10.368587	10.258215	11.163796	11.365635	11.398347
NM_0010069	MGC94704	Rattus norvegicus evolutionarily conse	chr8	0.01558943	2.157429	11.307022	11.371293	11.746029	12.597037	12.575822	12.57942
NM_138866	Eif2b5	Rattus norvegicus eukaryotic translatic	chr11	0.04065665	2.152123	9.332103	9.715736	9.408358	10.394101	10.446754	10.932625
NM_0010248	RGD130980	Rattus norvegicus similar to RIKEN c	chr1	0.01514625	3.0500107	9.005929	8.949164	9.251012	11.009992	10.416644	10.605909
NM_0011275	Tmprss13	transmembrane protease, serine 13"	chr8	0.01679496	2.747712	3.3271234	2.743627	2.739972	4.4431314	4.2244306	4.5178533
NM_0010076	Wdr79	Rattus norvegicus WD repeat domain	chr10	0.00264198	2.3080075	10.061435	10.21734	10.114991	11.351233	11.302409	11.36007
NM_0011304	Recql4	RecQ protein-like 4	chr7	0.03596673	2.47864	7.1474466	7.326955	6.9792166	8.077727	8.530096	8.774442
NM_0010994	Vom2r57	vomeronasal 2 receptor 57	chr7	0.01432293	2.8534338	2.7508433	3.3450615	2.7557	4.520253	4.5034585	4.3659897
NM_0011057	Nola2	nucleolar protein family A, member 2'	chr10	0.01043772	2.1424143	12.115728	11.895815	11.958601	13.364433	13.067566	12.835857
NM_0011133	Npy1r	neuropeptide Y receptor Y1	chr16	0.01055468	2.0269198	9.703872	9.28253	8.715163	10.520903	10.35056	9.887968
NM_0010391	Tor1b	Rattus norvegicus torsin family 1, mer	chr3	0.01774234	2.1768427	11.363071	11.529788	11.291788	12.568871	12.358004	12.624484
NM_0010034	Il22ra2	Rattus norvegicus interleukin 22 recep	chr1	0.04886242	4.045391	5.373993	5.796426	5.564315	6.9744964	7.3043604	8.504715
NM_0010109	Cndp2	Rattus norvegicus CNDP dipeptidase 2	chr18	0.02992199	2.164769	11.306317	11.328311	11.578935	12.813702	12.219233	12.523268
NM_0011088	Ubxn6	UBX domain protein 6	chrUn	0.04098916	2.083443	10.454115	10.647933	10.630121	11.494172	11.333479	12.081425
NM_0010243	LOC500377	Rattus norvegicus similar to Tubulin a	hr4_randoi	0.0209293	2.5712624	7.250502	8.055205	7.1603556	8.8076935	9.62345	8.122351
NM_0011356	Wdr4	WD repeat domain 4	chr20	0.01601088	2.2954533	10.347188	10.403109	10.459748	11.756544	11.303045	11.746794
NM_133380	Il4ra	Rattus norvegicus interleukin 4 recept	chr1	0.02283981	2.0827804	9.509956	8.8344755	9.147355	10.634867	10.135792	9.896663
NM_031075	P2rx3	Rattus norvegicus purinergic receptor	chr3	0.01748307	2.8559568	9.174773	9.648093	9.262142	10.851318	10.758872	11.016739
NM_0010372	Mmd2_pred	Rattus norvegicus monocyte to macroj	chr12	0.01175686	2.3776157	9.796198	9.901854	9.242069	10.820206	11.13028	10.738181
NM_0011072	Nfatc4	nuclear factor of activated T-cells,	chr15	0.00618755	3.0548744	7.940864	7.4370637	7.341194	9.802238	8.963797	8.786426
NM_0010318	Bmp10	bone morphogenetic protein 10	chr4	0.02680348	4.144261	2.7667418	2.7694657	2.775581	4.8065786	5.4197693	4.2387853
NM_0010076	C1qtnf1	Rattus norvegicus C1q and tumor necr	chr10	0.04723948	2.5505817	8.588577	8.171833	8.430752	10.387387	9.65592	9.200331
NM_0010994	Vom2r56	vomeronasal 2 receptor 56	chr7	0.04711589	2.8699782	2.7552366	3.0403795	2.7606063	4.8837686	3.9840207	4.2515535

NM_0011086	Gpr162	G protein-coupled receptor 162	chr4	0.01686852	2.1536407	5.8334055	6.3777685	6.3896675	6.9891844	7.211463	7.720527
NM_198972	Gkn1	Rattus norvegicus gastrokine 1 (Gkn1)	chr4	0.03728422	2.2922974	3.9129558	3.5868447	2.736349	5.5587535	4.423372	3.8444066
NM_012824	Apoc1	Rattus norvegicus apolipoprotein C-I (	chr1	0.02493057	2.392729	4.758406	4.2875586	3.717041	6.394591	5.484628	4.6597576
NM_0011074	Slc2a12	solute carrier family 2 (facilitated gluc	chr1	0.04328307	2.7030754	8.713597	9.186123	8.39992	10.548535	10.013804	10.041108
NM_0010005	Olr1307	Rattus norvegicus olfactory receptor 1	chr8	0.00611861	2.3664892	5.2997265	5.1095147	4.6086173	6.48157	6.222	6.0425334
NM_0010009	Olr308_pred	Rattus norvegicus olfactory receptor 3	chr1	0.01130746	2.3468063	2.7622185	2.765641	2.7731204	4.106446	3.7331834	4.153447
NM_0011095	Chml	choroideremia-like (Rab escort protein	chr13	0.00294113	2.0160873	5.7447867	5.527625	5.160286	6.853587	6.4461045	6.1676793
NM_0011092	Plekha8	pleckstrin homology domain containin	chr4	0.03237238	2.5954847	2.7464702	2.7482216	2.7502775	4.6162906	3.9789045	3.7777863
NM_0011096	Gys1	glycogen synthase 1 (muscle)	chr1	0.02939319	2.7668922	9.9579735	10.219502	9.539578	11.868064	11.237916	11.015872
NM_0010243	Lysmd1	Rattus norvegicus LysM, putative pept	chr2	0.04272334	2.1104267	10.278349	10.609386	10.097862	11.629089	11.229466	11.359647
NM_019247	Pitx3	Rattus norvegicus paired-like homeod	chr1	0.03893182	2.669293	4.678622	5.052798	5.8024373	6.5877085	5.964578	7.2309427
NM_013158	Dbh	Rattus norvegicus dopamine beta hydr	chr3	0.01839523	4.424386	7.9513083	7.8986907	6.8665123	10.612543	10.034693	8.505707
NM_0010243	nod3l	hypothetical protein LOC501101	chr9	0.0157878	2.4411218	10.669448	10.612543	11.000598	12.220052	11.937428	11.987743
NM_0011066	Gpx7	glutathione peroxidase 7	chr5	0.01923295	2.1362145	9.557903	9.674686	9.385361	10.476734	10.63889	10.787498
NM_0010123	Dgat2	Rattus norvegicus diacylglycerol O-ac	chr1	0.03420406	3.0194225	8.474016	8.4206085	8.498826	9.845153	10.613776	9.717342
NM_0010076	Pigq	Rattus norvegicus phosphatidylinosito	chr10	0.01807668	2.4440184	13.191073	13.368549	13.374611	14.822105	14.55789	14.422009
NM_0010001	Olr44_predi	Rattus norvegicus olfactory receptor 4	chr1	0.02550572	3.8225567	5.643586	5.0427713	5.3668175	7.283032	7.606926	6.9668317
NM_0011067	Hoxc12	homeo box C12	chr7	0.00661806	3.761455	3.6622334	3.5267522	2.7783382	5.526188	5.7292438	4.445764
NM_0011086	Fam176b	hypothetical protein LOC362597	chr5	0.01505333	2.2743938	9.849812	9.551208	9.581758	11.240774	10.816329	10.482121
NM_0010122	Serpinb6b	Rattus norvegicus serine (or cysteine)	chr17	0.03752884	2.0471137	9.740994	8.987086	8.196997	10.403657	10.050538	9.571656
NM_012867	Ninj1	Rattus norvegicus ninjurin 1 (Ninj1), r	chr17	0.03778564	2.0626097	11.417631	10.907354	11.601738	12.39362	12.343174	12.323343
NM_012750	Gfra2	Rattus norvegicus glial cell line derive	chr15	0.03059105	2.428996	8.736229	7.7281313	8.114153	9.819786	9.465493	9.134316
NM_0011077	RGD130652	hypothetical protein LOC310764	chr2	0.02112028	2.5284045	8.954726	8.956283	8.075281	10.298372	9.949534	9.753067
NM_0011062	Lix1	Lix1 homolog	chr1	0.03410577	2.533319	6.5877085	6.909828	6.2270827	7.9259458	7.8119597	8.0098
NM_080481	Atp5i	Rattus norvegicus ATP synthase, H+ t	chr14	0.02915521	2.088959	15.505056	15.855818	15.359539	16.80368	16.552544	16.552544
NM_0011057	Nthl1	nth endonuclease III-like 1	chr10	0.01956924	2.2415185	8.96715	8.645481	8.832106	10.21734	10.043682	9.677144
NM_0010006	Olr749_pred	Rattus norvegicus olfactory receptor 7	chr3	0.03618314	2.740161	3.321204	3.77347	2.7605639	4.5692797	4.8717227	4.777018
NM_198759	Arhgap27	Rattus norvegicus Rho GTPase activat	chr10	0.02497114	2.0128222	5.111186	5.365044	4.8504186	6.188687	6.0648766	6.100744
NM_0010243	Gimap7	GTPase, IMAP family member 7"	chr4	0.04678703	2.1872902	9.198391	8.011603	8.207064	9.846365	9.2445755	9.713553
NM_0010007	Olr876_pred	Rattus norvegicus olfactory receptor 8	chr7	0.0038347	2.530299	8.215708	9.099012	7.902491	9.44506	10.385192	9.404883
NM_0010174	LOC361914	Rattus norvegicus similar to solute car	chr2	0.00948112	3.0048811	7.8172035	8.225386	7.990262	9.453352	9.52199	9.819436
NM_0011345	RGD156307	hypothetical protein LOC305812	chr15	0.04693203	3.0872576	4.045147	2.7957206	2.755722	4.9460297	4.859963	4.669575
NM_0011346	Cytl1	cytokine like 1	chr14	0.02683484	2.193412	11.994416	10.651768	10.682779	12.941826	12.163848	11.622817

NM_053870	Kcnj4	Rattus norvegicus potassium inwardly-	chr7	0.00576977	2.2076128	5.153009	5.154975	4.8586283	6.2267246	6.4705677	5.89678
NM_0010120	Serpinc1	Rattus norvegicus serine (or cysteine)	chr13	0.0027825	2.9189234	4.1750407	4.102029	3.4599857	5.7303386	5.7837706	4.859256
NM_012751	Slc2a4	Rattus norvegicus solute carrier family	chr10	0.00953081	2.2176528	7.131851	6.597852	6.5670843	8.059571	7.818022	7.866295
NM_0011061	Dym	dymeclin	chr18	0.01394315	2.2364538	11.731962	11.829773	11.937428	13.144382	12.966756	12.871663
NM_030997	Vgf	Rattus norvegicus VGF nerve growth	chr12	0.000547	2.047915	8.649491	8.912512	8.564589	9.656369	9.9256935	9.646997
NM_0010083	Rac2	Rattus norvegicus RAS-related C3 bot	chr7	0.0132297	2.373045	10.776492	9.310858	9.620233	11.803237	10.50438	11.140184
NM_0010131	Rfxank	Rattus norvegicus regulatory factor X-	chr16	0.01620764	2.152816	10.628984	10.727052	10.652618	11.972276	11.577522	11.777533
NM_053688	Pde6h	Rattus norvegicus phosphodiesterase 6	chr4	0.04851729	2.0627112	5.4562516	5.678761	5.526586	6.9291415	6.325967	6.5401144
NM_0010001	Olr159_pred	Rattus norvegicus olfactory receptor 1	chr1	0.03706755	2.5013762	2.7371325	2.735914	2.742158	4.325918	3.5346496	4.322802
NM_0010005	Olr1381_pre	Rattus norvegicus olfactory receptor 1	chr10	0.01025529	4.022345	2.7484314	2.750781	3.5559695	5.150215	4.463396	5.465681
NM_017069	Gabra3	Rattus norvegicus gamma-aminobutyr	chrX	0.02872355	3.2428854	4.07022	2.741155	2.747649	6.093074	3.8515496	4.706234
NM_021688	Kcnk1	Rattus norvegicus potassium channel,	chr19	0.02409822	2.0134869	12.40016	12.076257	12.385977	13.154253	13.379454	13.357776
NM_053292	Vars	valyl-tRNA synthetase	chr20	0.00599537	2.069828	12.666533	12.380661	12.847731	13.593852	13.585037	13.864567
NM_031747	Cnn1	Rattus norvegicus calponin 1 (Cnn1), r	chr8	0.000836	3.5593777	9.470114	8.493619	8.495941	11.202621	10.407322	10.344608
NM_0011084	Nomo1	nodal modulator 1	chr1	0.000492	2.048351	13.875061	14.112839	13.811415	14.953456	15.136936	14.812313
NM_199101	Plekha4	Rattus norvegicus pleckstrin homolog	chr1	0.01489284	2.4797947	8.850292	8.281876	8.806927	10.189916	9.856318	9.823523
NM_019198	Fgf17	Rattus norvegicus fibroblast growth fa	chr15	0.03126637	3.1133635	4.9397326	5.2054834	4.841518	6.5634594	6.337549	7.0011477
NM_133420	Chrna2	Rattus norvegicus cholinergic receptor	chr15	0.00162937	2.2295961	2.755457	2.824994	2.7648342	3.9930723	3.9006593	3.9219015
NM_022864	Cplx1	Rattus norvegicus complexin 1 (Cplx1	chr14	0.02196927	3.3851688	8.260496	8.864248	7.8464622	10.192068	10.102995	9.953827
NM_0011072	Sall2	sal-like 2	chr15	0.01547262	2.2669234	10.40341	10.449342	10.260863	11.860817	11.397741	11.397264
NM_0010042	Pa2g4	Rattus norvegicus proliferation-associ	chr7	0.02459682	2.5942123	9.991604	9.826145	10.414785	11.507553	11.49175	11.359122
NM_0010008	Olr1622_pre	Rattus norvegicus olfactory receptor 1	chr15	0.01955042	2.9644375	3.4773693	2.786185	2.7856927	5.086342	3.9496393	4.7165413
NM_0010007	Olr209_pred	Rattus norvegicus olfactory receptor 2	chr1	0.00307418	3.1267912	6.3139205	6.3371086	6.292119	7.893012	8.162356	7.821829
NM_017342	Sftpc	Rattus norvegicus surfactant associate	chr15	0.04423914	2.5431461	2.78156	2.7882838	2.8016267	4.6903577	3.7102957	4.0106597
NM_031701	Cldn5	Rattus norvegicus claudin 5 (Cldn5), m	r11_rando	0.01060626	4.938748	7.877692	6.876765	7.5595374	10.51771	9.308012	9.400709
NM_0010003	Olr769_pred	Rattus norvegicus olfactory receptor 7	chr3	0.00114038	4.064098	2.7754261	2.786845	2.7857385	4.781853	4.700476	4.934485
NM_033359	Ngb	Rattus norvegicus neuroglobin (Ngb),	chr6	0.04407923	2.1724827	9.076916	8.8453	8.942948	10.569033	10.048632	9.605534
NM_012491	Add2	Rattus norvegicus adducin 2 (beta) (A	chr4	0.01292253	2.0426738	6.432746	6.4756665	6.8444333	7.3905997	7.3474317	8.106191
NM_145784	Gpr37l1	G protein-coupled receptor 37-like 1	chr13	0.00253471	2.7319167	5.8548126	6.102319	5.451414	7.2922616	7.43225	7.0337753
NM_0010479	LOC501706	Rattus norvegicus hypothetical protein	chr10	0.01147824	3.3078833	4.474045	2.7572522	4.656739	5.9716716	4.852805	6.2412863
NM_017240	Myh7	Rattus norvegicus myosin, heavy poly	chr15	0.04293954	3.1134899	8.603326	8.836316	8.161414	10.772156	9.811501	9.932996
NM_0010147	Nbpwr1	Rattus norvegicus neuropeptides B/W	chr5	0.00957495	3.2503006	2.7594383	2.7634306	2.7684278	4.1249084	4.639225	4.628883
NM_0011086	Ccl27	chemokine (C-C motif) ligand 27	chr5	0.0199072	2.1087518	9.077638	9.08949	9.091095	9.877696	10.185521	10.424172

NM_0010000	Olr1516_pre	Rattus norvegicus olfactory receptor 1	chr10	0.02562144	2.3731253	4.324593	3.905713	2.7663176	5.4040623	5.557473	3.7754536
NM_0011090	Golt1a	golgi transport 1 homolog A	chr13	0.0323497	2.5288174	10.866301	11.211556	10.988625	12.692455	12.240631	12.148787
NM_053902	Kynu	Rattus norvegicus kynureninase (L-ky	chr3	0.0106249	2.7401888	2.8050709	2.827663	2.8928719	4.3761506	3.9821448	4.530136
NM_0011067	Saps2	SAPS domain family, member 2"	chr7	0.04858534	2.3397975	6.3000994	6.2685103	6.06851	8.076939	7.3147793	6.924553
NM_0010391	Tusc5	Rattus norvegicus tumor suppressor ca	chr10	0.04570834	2.7758508	8.725602	8.59913	7.7090006	10.022169	9.61629	9.814064
NM_0010479	LOC500846	Rattus norvegicus hypothetical protein	chr7	0.02947742	2.093459	3.3855402	2.741536	4.423372	4.7150903	3.905713	5.12731
NM_0010070	MGC94915	Rattus norvegicus similar to hypotheti	chr6	0.03145079	2.0996509	7.248388	7.4510207	7.082532	8.473037	8.134935	8.384417
NM_031766	Cpz	Rattus norvegicus carboxypeptidase Z	chr14	0.0373662	2.4608254	7.707815	7.4761567	7.1255345	9.4763	8.353163	8.377469
NM_0010001	Olr40_predi	Rattus norvegicus olfactory receptor 4	chr1	0.0330134	2.0518732	3.1171436	2.831302	2.8958845	4.3158894	4.09127	3.5479946
NM_0010340	Syn2	synapsin II isoform 1	chr4	0.00338928	2.3816419	7.716495	7.8327355	7.542781	9.108366	8.978179	8.761335
NM_0010002	Olr440_pred	Rattus norvegicus olfactory receptor 4	chr3	0.02512077	2.1589026	2.8806357	2.739875	2.7401547	3.633048	4.0075793	4.050933
NM_0011375	Gsx2	GS homeobox 2	chr14	0.02089332	3.9177895	7.339363	7.34149	7.0390706	9.637757	8.734746	9.25754
NM_0011074	Kptn	kaptin (actin binding protein)	chr1	0.04139818	2.3033466	9.038341	8.05546	9.479206	10.391235	9.603048	10.189916
NM_0010141	Mall	Rattus norvegicus mal, T-cell different	chr3	0.02260535	2.6530237	9.030721	8.14612	8.03773	10.153906	9.41601	9.867571
NM_0011052	Mrvi1	MRV integration site 1 homolog isofo	chr1	0.00563902	2.67057	9.873594	9.089066	8.828825	11.116458	10.486195	10.440273
NM_0010375	Defb21	beta-defensin 21	chr3	0.02252264	3.1145976	2.7513561	2.7592385	2.7579167	4.737695	3.9125772	4.5353756
NM_0010039	G4	Rattus norvegicus G4 protein (G4), ml	chr20	0.02476351	2.8351548	9.648093	9.983193	9.727255	11.461318	11.011742	11.395764
NM_175578	Dscr1l1	Rattus norvegicus Down syndrome cri	chr9	0.04441942	2.1659741	11.693621	12.006251	12.20628	13.290113	12.82103	13.140055
NM_0011092	RGD155949	hypothetical protein LOC500516	chr5	0.03289872	3.0418706	4.235673	2.781364	2.7759433	5.297744	4.442581	4.867532
NM_0011094	LOC680454	hypothetical protein LOC680454	chr10	0.01011328	2.0922136	6.8221893	6.3771944	6.604884	7.905018	7.2470307	7.84731
NM_0010776	Myl1	fast myosin alkali light chain 1f	chr9	0.0127144	3.3641806	7.974357	7.4833136	6.878362	9.419476	9.164141	9.003184
NM_0010141	RGD130793	Rattus norvegicus similar to Hypotheti	chr10	0.03205257	2.0031173	10.967148	10.607958	10.797717	11.781669	11.977946	11.619946
NM_013189	Gnaz	Rattus norvegicus guanine nucleotide	chr20	0.04627392	2.0690303	2.7469788	2.9434423	3.048264	4.263607	3.7605739	3.8613687
NM_0011060	Tssk6	testis-specific serine kinase 6	chr16	0.01388202	2.496498	5.796426	5.6513405	6.076441	7.0918593	7.2548566	7.137209
NM_0010010	Olr1105_pre	Rattus norvegicus olfactory receptor 1	chr7	0.01044013	2.5673425	6.7220664	6.5729046	6.9012322	8.008442	8.204179	8.064412
NM_0011084	Cog4	component of oligomeric golgi compl	chr19	0.00529597	2.0209568	12.100584	11.856578	12.159465	12.995372	12.856543	13.30983
NM_0011073	RGD156345	hypothetical protein LOC306883	chr17	0.01543251	3.4063342	4.6898327	4.14026	4.369902	6.8777175	5.5718174	6.055118
NM_0011062	Rpl27a	ribosomal protein L27a	chr1	0.04350694	2.5283728	6.9441237	7.4584303	6.5275908	8.156666	8.371536	8.416571
NM_0010005	Olr742_pred	Rattus norvegicus olfactory receptor 7	chr3	0.03809086	2.0263317	2.7888522	2.7962515	2.8022287	4.204866	3.5301023	3.708976
NM_0010349	Tarbp2	Rattus norvegicus TAR (HIV) RNA b	chr7	0.01576816	2.0797758	11.067966	11.333479	11.5265	12.383387	12.321743	12.392101
NM_0011066	Frmd3	FERM domain containing 3	chr5	0.00833643	2.0450356	6.3108864	6.189416	6.014631	7.3474317	7.38354	6.880339
NM_013078	Otc	Rattus norvegicus ornithine transcarba	chrX	0.0085217	2.2598546	7.3845816	7.777534	6.9363375	8.350418	9.007698	8.269028
NM_019621	Dlgh4	Rattus norvegicus discs, large homolo	chr10	0.00638664	2.3747602	10.528566	10.88207	10.843868	11.974841	12.054482	11.968529

NM_0011080'	Myo1f	myosin IF	chr7	0.03893981	2.188359	9.466185	8.438322	8.888035	10.317163	9.3914	10.473525
NM_0010036:	Pard6a	Rattus norvegicus par-6 (partitioning c	chr19	0.0406966	2.0034072	8.490755	8.598725	8.09781	9.350302	9.33309	9.511264
NM_0011094:	Rprml	reprimo-like	chr10	0.01664855	2.9721825	9.533812	9.586948	9.347358	10.80538	11.065508	11.3118
NM_0010247·	Chaf1b	Rattus norvegicus chromatin assembly	chr11	0.00347288	2.9483454	7.1851125	6.9792166	7.168492	8.586797	8.700141	8.725602
NM_0010089:	V1rc34	vomeronasal 1 receptor, C34"	chr4	0.01805676	2.8209224	2.8751874	2.8096716	2.7976232	4.081105	4.6988397	4.1910377
NM_0010005'	Olr1091_pre	Rattus norvegicus olfactory receptor 1	chr7	0.02618475	2.2856693	2.7543309	2.7636054	2.7575746	4.2170825	3.5730667	4.0632124
NM_173304	Cyp2d10	Rattus norvegicus cytochrome P450, f	chr7	0.04710682	2.5787165	2.7592795	2.7662334	2.7609546	4.7389407	3.7815382	3.8659477
NM_0010002:	Olr276_pred	Rattus norvegicus olfactory receptor 2	chr1	0.0149623	2.8670447	3.4276416	2.766179	2.7606692	5.0347137	3.9253235	4.5531464
NM_0011096·	LOC691729	hypothetical protein LOC691729	chr3	0.02257641	2.044931	8.420233	8.569658	8.785782	9.3914	9.36405	10.116379
NM_0010249'	Samd14	Rattus norvegicus sterile alpha motif d	chr10	0.01084105	3.040919	6.553094	5.9373145	5.911875	7.825426	7.659376	7.731002
NM_053991	Vip	vasoactive intestinal peptide	chr1	0.01658727	5.3134217	9.409968	9.435669	7.824862	12.439484	11.445354	10.014585
NM_0010252'	Mbp	Rattus norvegicus myelin basic proteir	chr18	0.00801578	3.7342026	7.361843	7.3399334	7.0317817	9.213644	8.97174	9.250578
NM_0011009'	Aebp1	AE binding protein 1	chr14	0.00966678	2.7433293	11.600756	10.987646	11.220454	12.775563	12.63985	12.761228
NM_0011276:	Ckmt2	sarcomeric mitochondrial creatine kin:	chr2	0.00463806	2.2939765	5.4948525	5.076478	4.7684684	6.533441	6.3868403	6.01307
NM_0010470'	Ythdf2_pred	Rattus norvegicus YTH domain family	chr5	0.03516085	2.097979	11.915121	12.48208	11.69082	13.045402	13.167744	13.081875
NM_139329	Hsd3b7	Rattus norvegicus hydroxy-delta-5-ste:	chr1	0.00249088	2.5200264	9.634491	9.797321	9.489712	11.055626	10.999914	10.866301
NM_144743	Ces6	Rattus norvegicus carboxylesterase 6 (	chr19	0.04411062	2.2773182	6.172658	5.8890963	6.0532317	7.276396	7.559171	6.841428
NM_0010252'	Mbp	myelin basic protein isoform 2	chr18	0.02814971	4.1555896	7.2894006	7.3916807	6.5033035	9.203318	8.91951	9.226716
NM_0011094'	Ddo	D-aspartate oxidase	chr20	0.04033176	3.0004241	6.4417768	6.3590226	6.48342	8.605309	7.924423	7.509986
NM_0010089·	V1rd23	vomeronasal 1 receptor, D23"	chr1	0.00614951	2.2354565	2.7681575	2.783789	2.774013	4.0367017	3.7625592	4.008408
NM_0010076	Wipi2	Rattus norvegicus WD repeat domain,	chr12	0.02425647	2.0073602	10.116379	10.388907	10.087997	11.071592	11.146437	11.39115
NM_031607	Hspb7	cardiovascular heat shock protein	chr5	0.01158795	2.7870765	5.4179564	4.479747	4.8686	6.6797	6.2720428	6.250819
NM_133562	Pib5pa	Rattus norvegicus phosphatidylinosito	chr14	0.00307178	2.653243	9.38792	9.424246	9.473072	10.751623	10.983997	10.772887
NM_0011081:	Mfrp	membrane frizzled-related protein	chr8	0.04235556	2.591349	2.7727108	2.794892	2.7789583	4.6153417	3.6324608	4.2198687
NM_172042	Kcnk2	Rattus norvegicus potassium channel,	chr13	0.04736238	2.5836225	9.059258	9.645447	8.56014	10.551537	10.428688	10.392804
NM_0010246:	Tas2r38	taste receptor, type 2, member 38"	chr4	0.01089693	2.2842114	2.767676	2.7810621	2.7726126	3.7691104	3.926306	4.201023
NM_0011072:	Osbp2	oxysterol binding protein 2	chr14	0.00611448	2.4004385	8.377243	8.553621	8.321624	9.712735	9.620708	9.708939
NM_019383	Atp5h	Rattus norvegicus ATP synthase, H+ t	chr10	0.0122827	2.0550618	11.934803	11.848467	12.22196	13.099193	12.99469	13.028892
NM_0010077:	Scly	Rattus norvegicus selenocysteine lyase	chr9	0.01134944	2.1469212	11.265684	11.101547	11.293304	12.305865	12.432834	12.228649
NM_0011085'	Bccip	BRCA2 and CDKN1A interacting pro	chr1	0.000822	2.1930103	5.8238544	5.5341945	2.8097234	7.010509	6.6085825	3.9474201
NM_0011066:	Mrps35	mitochondrial ribosomal protein S35	chr4	0.00877495	5.882854	5.2537594	5.5385904	4.791586	8.093222	7.6155496	7.544714
NM_0010338'	RGD130835	Rattus norvegicus similar to Hypotheti	chr10	0.01506485	2.0293276	6.707716	6.2526546	6.4391117	7.506152	7.490047	7.4662895
NM_0011090'	Ascc2	activating signal cointegrator 1 comple	chr14	0.03764549	2.2937121	10.200054	9.941419	10.690299	11.450034	11.524756	11.450034

NM_0010173	Paqr4	Rattus norvegicus progestin and adipo	chr10	0.00157735	2.7276337	8.942948	8.454321	8.642495	10.44105	9.966362	9.975301
NM_013010	Prkag1	Rattus norvegicus protein kinase, AMI	chr7	0.000654	2.1309144	11.117998	11.031757	11.382765	12.252847	12.132005	12.422086
NM_0010148	Faim3	Rattus norvegicus Fas apoptotic inhibi	chr13	0.02322939	5.168683	9.135487	8.362599	7.3935585	11.72353	10.015233	10.262272
NM_022259	Cd244	Rattus norvegicus CD244 natural kille	chr13	0.01326161	2.2926939	2.7774978	2.7954974	2.9741602	4.0878778	3.7155805	4.3348284
NM_0010142	RGD135924	Rattus norvegicus ankyrin repeat conta	chr9	0.0024691	2.3360043	8.7556095	8.841111	8.585227	9.8633	10.154731	9.836049
NM_0010174	MGC10882	Rattus norvegicus similar to RIKEN c	chr13	0.01874284	2.6652625	5.50497	5.508668	5.337772	7.308416	6.774415	6.511411
NM_0011083	Zcchc24	zinc finger, CCHC domain containing	chr16	0.00491346	2.274272	9.489712	9.032032	9.176499	10.512619	10.331284	10.410552
NM_0010778	Tmem58_pr	Rattus norvegicus transmembrane prot	chr13	0.03576278	2.5591965	8.55892	8.74481	9.047404	10.315932	9.604042	10.498232
NM_0010005	Olr859_pred	Rattus norvegicus olfactory receptor 8	chr5	0.02469388	2.8969743	6.66762	6.955375	6.574116	8.630049	8.066651	8.10405
NM_0010085	C1qa	Rattus norvegicus complement compo	chr5	0.0335669	2.0273836	12.701951	11.299089	11.812197	13.469343	12.194835	13.207919
NM_053850	Blvra	Rattus norvegicus biliverdin reductase	chr3	0.02227611	2.0159385	10.994234	10.777842	10.963919	11.734442	11.800247	12.23566
NM_0010243	LOC500445	hypothetical protein LOC500445	chr5	0.01885169	2.2266676	5.2319136	5.480772	5.111186	6.2042913	6.497485	6.586754
NM_0010318	Gpr33_pred	Rattus norvegicus G protein-coupled r	chr6	0.01054105	2.518409	2.7800694	2.7963986	2.7865808	4.1260977	3.8835883	4.3509
NM_0011083	Lhx4	LIM homeobox 4	chr13	0.00983157	2.0390563	6.5614967	6.7755284	6.3044834	7.3887267	7.865985	7.470502
NM_020100	Ramp3	Rattus norvegicus receptor (calcitonin	chr14	0.03299748	2.4917011	11.506259	11.125195	11.294344	12.997053	12.752992	12.127142
NM_0011084	Igfl3	IGF-like family member 3	chr1	0.00898176	3.9746144	2.8046029	2.8414993	2.8135989	4.4442296	5.1334066	4.8545094
NM_0010257	Fbxw5	Rattus norvegicus F-box and WD-40 c	chr3	0.00334946	3.202222	10.684423	10.724806	10.698587	12.547509	12.256403	12.341126
NM_0011376	Zfp259	zinc finger protein 259	chr8	0.02820048	2.1065845	4.370241	3.8574953	3.8077805	5.3494196	4.6717625	5.2390523
NM_0011095	Fam101a	hypothetical protein LOC689711	chr12	0.04069434	2.5019937	9.669077	9.730135	9.464508	10.61318	10.896601	11.323174
NM_0010120	Snx11	Rattus norvegicus sorting nexin 11 (Sr	chr10	0.00336796	2.017697	9.260658	9.429996	9.25251	10.245439	10.357523	10.378329
NM_019193	Sox10	Rattus norvegicus SRY-box containing	chr7	0.01189861	4.6915507	6.592254	6.6591043	5.7062516	8.859253	8.446769	8.34178
NM_0011072	Arhgap22	Rho GTPase activating protein 22	chr16	0.02676117	2.9565678	7.2180724	7.5600467	7.636265	9.2322645	8.669754	9.204131
NM_0011070	Arf4l	ADP-ribosylation factor 4-like	chr10	0.01617006	2.3153944	9.742156	9.716491	8.853377	11.089819	10.616686	10.239292
NM_0010123	RGD131009	Rattus norvegicus similar to 2310043F	chr16	0.02338082	2.6442196	7.01433	6.502868	6.4208765	8.536847	7.4821515	8.127602
NM_131909	Hrh4	Rattus norvegicus histamine H4 recept	chr18	0.0446154	2.1886165	8.14947	8.492773	8.215708	9.56726	9.131108	9.549639
NM_053907	Dnase1l3	Rattus norvegicus deoxyribonuclease I	chr15	0.03685946	2.9351552	7.721069	7.261727	6.935052	9.567726	8.201783	8.808646
NM_012893	Actg2	Rattus norvegicus actin, gamma 2 (Ac	chr4	0.01139831	5.2339544	10.560408	9.6638155	9.2999525	12.602469	11.894997	12.190415
NM_017239	Myh6	Rattus norvegicus myosin, heavy poly	chr15	0.02416742	3.511263	4.2088137	4.029253	2.8443272	5.6798215	5.6120315	5.22651
NM_0011429	RGD156107	tripartite motif-containing 43-like	chr8	0.00963028	2.066023	6.280371	6.3986034	6.315603	7.299549	7.636924	7.198674
NM_0010039	Gpsm3	Rattus norvegicus G-protein signalling	chr20	0.02476098	2.5084026	9.337262	9.0275755	9.165363	10.573765	10.039377	10.897367
NM_173139	Gnat3	Rattus norvegicus guanine nucleotide	chr4	0.02067981	3.2160394	6.575448	7.48055	6.451318	8.729072	9.063263	7.7708354
NM_0010122	Bin2	Rattus norvegicus bridging integrator 2	chr7	0.04122103	2.2833266	9.495679	8.6292305	8.964463	10.255573	9.81815	10.58906
NM_052808	Psp	Rattus norvegicus parotid secretory pr	chr3	0.00894515	2.040562	6.690297	6.8842087	6.767456	7.8953795	7.8995185	7.6339617

NM_0010096	Car8	Rattus norvegicus carbonic anhydrase	chr5	0.02822587	2.7944252	10.539932	10.961492	10.300188	11.785916	12.172031	12.291323
NM_0010076	RGD135933	Rattus norvegicus similar to hypotheti	chr2	0.00235738	2.6107187	9.242069	9.508779	9.923265	10.615514	10.782484	11.429457
NM_0011065	Cstl1	cystatin-like 1	chr3	0.0169156	2.1030838	5.851689	5.8298106	5.63425	6.9176784	7.1502304	6.4653606
NM_133426	Kcng3	Rattus norvegicus potassium voltage-g	chr6	0.02532321	6.531386	3.2739098	3.4618754	3.0391757	6.859736	5.7323136	5.3050795
NM_0011078	Stard8	StAR-related lipid transfer (START) d	chrX	0.01535174	3.264352	8.951925	9.005095	8.928203	10.853256	10.284569	10.867792
NM_130430	Psmd9	Rattus norvegicus proteasome (proson	chr12	0.03807239	2.1765463	9.344133	9.375827	9.588278	10.896601	10.398827	10.378933
NM_022301	Pik4ca	Rattus norvegicus phosphatidylinosito	chr11	0.04562831	2.351581	8.094564	8.677035	8.564394	9.500982	9.375827	10.160077
NM_0011093	Rab17	RAB17, member RAS oncogene famil	chr9	0.00382063	2.0442038	9.308012	8.82025	9.2402525	10.216923	9.944539	10.30167
NM_019325	Myh4	Rattus norvegicus myosin, heavy poly	chr10	0.01065382	5.081432	2.8058543	2.9221184	2.8573227	5.6376896	5.0565653	4.926746
NM_022261	Bspry	Rattus norvegicus B-box and SPRY do	chr5	0.01824221	2.1448672	10.277824	10.048632	10.228857	11.39115	11.404204	11.062624
NM_133581	Wfdc1	Rattus norvegicus WAP four-disulfide	chr19	0.04671058	2.0434227	9.871074	9.059258	9.183058	10.737272	9.7988615	10.670219
NM_0010007	Olr1406	olfactory receptor Olr1406	chr10	0.04863973	2.7458212	6.903876	7.4946704	6.5760674	8.687707	8.284591	8.374027
NM_0010339	Kcng3	potassium voltage-gated channel, subf	chr6	0.01060321	6.476079	3.659585	2.9939995	2.9273539	6.7416887	5.845579	5.0790324
NM_0010316	Coq4	Rattus norvegicus coenzyme Q4 homo	chr3	0.04411869	2.275654	8.435618	8.568428	8.764403	10.102704	9.67548	9.549115
NM_173299	Np4	Rattus norvegicus defensin NP-4 precu	chr16	0.03641752	2.2727494	6.0226293	5.9217005	5.3618007	6.7424245	7.326289	6.790733
NM_0011058	Nkiras2	NFKB inhibitor interacting Ras-like 2	chr10	0.009132	2.2440922	10.582218	10.739886	10.704969	11.97187	11.811607	11.741996
NM_0010375	Defb13	beta-defensin 13	chr16	0.03657126	3.2482748	8.704671	9.091814	8.43932	10.969234	10.199363	10.166231
NM_0010085	Slc10a4	Rattus norvegicus solute carrier family	chr14	0.04138217	4.845173	2.9147336	3.47912	3.1565866	6.1092463	5.5283704	4.742467
NM_0011094	Emid1	EMI domain containing 1	chr14	0.00730779	2.0934157	9.92809	9.69498	9.696515	11.084956	10.577602	10.854601
NM_0011064	Krtcap2	keratinocyte associated protein 2	chr2	0.02770745	2.0598395	10.158583	10.338528	10.141359	11.140681	11.108781	11.516601
NM_022230	Stc2	Rattus norvegicus stanniocalcin 2 (Stc	chr10	0.04558187	2.125276	5.663832	5.230711	5.8040895	6.624459	6.7836685	6.553456
NM_0010352	Myl2	Rattus norvegicus myosin, light polype	chr12	0.04749985	8.952269	4.395287	5.298127	3.044962	8.354123	7.0337753	6.837239
NM_0010091	Trim10	tripartite motif-containing 10	chr20	0.00965502	2.1561115	7.8951077	7.9716825	8.066651	9.083924	9.216735	8.958077
NM_0010130	Fhl5	Rattus norvegicus four and a half LIM	chr5	0.00599861	2.4897056	8.33183	8.491398	8.127092	9.833384	9.789537	9.275324
NM_053565	Socs3	Rattus norvegicus suppressor of cytok	chr10	0.01577613	2.40058	6.8444333	6.8674073	6.658528	8.424718	8.017595	7.718202
NM_0010009	Olr1445	olfactory receptor Olr1445	chr10	0.0024308	2.64533	7.609377	7.4594984	7.605485	8.927305	9.00021	8.95719
NM_017289	Gabrd	Rattus norvegicus gamma-aminobutyr	chr5	0.03618369	3.5876782	7.50216	7.855902	7.912246	10.050059	9.215589	9.533812
NM_0011094	Nkx6-3	NK6 homeobox 3	chr16	0.00576212	2.6035051	3.3917155	3.1971219	3.2104332	4.573788	4.737695	4.629153
NM_017168	Plcg2	Rattus norvegicus phospholipase C, ga	chr19	0.02432585	2.5711493	9.41143	9.354811	9.342195	10.931657	10.289193	10.974827
NM_0010003	Olr428_pred	Rattus norvegicus olfactory receptor 4	chr3	0.01345908	3.4762518	6.1805067	5.7552977	5.875843	8.260496	7.6823587	7.26139
NM_0011092	RGD156043	hypothetical protein LOC500546	chr5	0.01004267	2.3129628	5.107663	5.3534555	5.047443	6.3813934	6.3270125	6.429382
NM_0010003	Olr485_pred	Rattus norvegicus olfactory receptor 4	chr3	0.02661395	2.1988878	4.409919	4.1693316	3.4906163	5.4149566	5.064678	5.000553
NM_0011092	Lbx2	ladybird homeobox 2	chr4	0.03836958	2.0708756	7.5545487	7.905543	7.1816	8.97174	8.588798	8.231876

NM_019308	Gja6	Rattus norvegicus gap junction membr	chrX	0.01844528	2.5119677	2.866101	3.2014155	3.2444465	4.5245304	4.2275934	4.546293
NM_0010121	Cd97	Rattus norvegicus CD97 antigen (Cd9	chr19	0.00150831	2.0112479	9.2787075	8.656558	9.047726	10.235643	9.638784	10.132835
NM_053595	Pgf	Rattus norvegicus placental growth fa	chr6	0.01610462	2.3955398	7.9502225	7.5193405	7.3538322	9.534121	8.6292305	8.441095
NM_023091	Gabre	Rattus norvegicus gamma-aminobutyr	chrX	0.03664518	2.0065696	7.823376	7.731002	7.2281127	9.123862	8.360287	8.312535
NM_0010010	Olr544_pred	Rattus norvegicus olfactory receptor 5	chr3	0.04096144	3.579722	5.730999	5.446387	5.574669	6.957612	7.9940434	7.319942
NM_020087	Notch3	Rattus norvegicus Notch gene homolo	chr7	0.04527948	3.0540476	8.001468	8.541523	8.329792	9.947497	9.442774	10.314683
NM_0011062	Rasip1	Ras interacting protein 1	chr1	0.00828211	2.2267044	11.044125	10.931657	10.818069	12.352086	11.883596	12.022897
NM_0011059	Mmp17	matrix metallopeptidase 17	chr12	0.02949084	2.3230782	8.299373	7.908388	8.289461	9.48578	9.5082855	9.15127
NM_0010392	Zdhhc19	Rattus norvegicus zinc finger, DHHC	chr11	0.00240601	4.0517435	3.977333	3.8342748	4.2198687	5.8278427	6.028158	6.2311044
NM_053733	Bcl2l10	Rattus norvegicus Bcl2-like 10 (Bcl2l	chr8	0.03938374	2.4654775	7.5140405	7.990779	7.386295	9.297548	8.854977	8.644192
NM_0010005	Olr858_pred	Rattus norvegicus olfactory receptor 8	chr5	0.01840903	2.3066669	6.8598957	7.179472	7.148978	8.089987	8.086558	8.6292305
NM_0011094	LOC681325	hypothetical protein LOC681325	chrX	0.04162987	2.343546	5.665205	5.4136243	5.154975	6.922181	6.1805067	6.817194
NM_0011063	Pnma3	paraneoplastic antigen MA3	chrX	0.03531217	2.20272	7.1063523	7.364779	7.1775417	8.646273	8.146382	8.273877
NM_172328	Tac4	Rattus norvegicus tachykinin 4 (Tac4)	chr10	0.0240471	2.3045895	9.501845	9.857247	10.111514	10.706546	10.732169	11.645423
NM_012633	Prph1	Rattus norvegicus peripherin 1 (Prph1	chr7	0.0047581	8.182665	8.573087	8.261433	7.1990647	12.025423	11.092066	10.013804
NM_0010257	Grap	Rattus norvegicus GRB2-related adap	chr10	0.01444885	2.5958507	8.871008	8.77249	8.572323	10.548991	9.872874	9.922576
NM_019180	Mcpt6	Rattus norvegicus mast cell protease 6	chr10	0.0456786	2.798975	7.7582293	7.966325	7.8126473	9.590896	8.794135	9.606868
						7.7582293	7.966325	7.8126473	9.590896	8.794135	9.606868
