# Supplementary material for: Role of microRNAs in the age-associated decline of pancreatic beta cell function in rat islets
Source: Diabetologia. 2015 Oct 16;59(1):161–9. doi: 10.1007/s00125-015-3783-5 (PMC4670458; doi:10.1007/s00125-015-3783-5)
Supplement: Supplementary file 9 — (PDF 121 kb) [file 125_2015_3783_MOESM9_ESM.pdf]

ESM Table 3

Pathway analysis of the genes differentially expressed between 3- and 12-month-ol rats

Definition	Fischer-P-value	FDR	Enrichment	Genes
Long-term potentiation	0.0000749	0.00992150	4.125084	ARAF//CACNA1C//CALM1//CALM2//CAMK2D//CREBBP/PPP3CB//PPP3R1//RAP1B
Amphetamine addiction	0.0000749	0.00992150	4.125084	ATF2//CACNA1C//CACNA1D//CALM1//CALM2//CAMK2D//CREB1//PPP3CB//PPP3R1
Renal cell carcinoma	0.0001074	0.00992150	3.968781	ARAF//CREBBP//GAB1//HIF1A//PAK1//PAK2//PAK3//RAP1B//TCEB1
Insulin secretion	0.0001497	0.0103721	3.824553	ATF2//ATP1A3//CACNA1C//CACNA1D//CAMK2D//CREB1//GPR119//KCNN2//RIMS2//SLC2A2
Axon guidance	0.0002761	0.01529967	3.558828	CFL2//DPYSL2//GNAI1//PAK1//PAK2//PAK3//PPP3CB//PPP3R1//RASA1//ROCK2//SEMA4A//SEMA4F
MAPK signaling pathway	0.0009936	0.04587429	3.002759	ATF2//CACNA1C//CACNA1D//CHUK//DUSP1//DUSP6//MAP3K12//PAK1//PAK2//PPM1A//PPP3CB//PPP3R1//RAP1B//RASA1//RASA2//RPS6KA5//TP53
Ubiquitin mediated proteolysis	0.0014047	0.04967515	2.852401	CUL4B//HERC3//KLHL9//NEDD4//RCHY1//TCEB1//UBA6//UBE2A//UBE2D1//UBE2G1//UBE2Q1
Circadian entrainment	0.0014346	0.04967515	2.843251	CACNA1C//CACNA1D//CALM1//CALM2//CAMK2D//CREB1//GNAI1//GNG5//RPS6KA5
Adrenergic signaling in cardiomyocytes	0.0029196	0.08986143	2.534664	ATF2//ATP1A3//CACNA1C//CACNA1D//CALM1//CALM2//CAMK2D//CREB1//GNAI1//RPS6KA5//SCN7A
Maturity onset diabetes of the young (MODY)	0.0055674	0.1401976	2.254346	HHEX//NEUROD1//PAX6//SLC2A2

ESM Table 3

Pathway analysis of the genes differentially expressed between 3- and 12-month-ol rats

Hepatitis B	0.0062196	0.1435713	2.206231	ATF2//CDKN1B//CHUK//CREB1//CREBBP//SMAD4//TLR3//TLR4//TP53//YWHAZ
MicroRNAs in cancer	0.0071903	0.1532087	2.143253	BMI1//CDKN1B//CREBBP//GLS//RECK//RPS6KA5//SERPINB5//SLC7A1//TP53//WNT3
Ras signaling pathway	0.0126560	0.208592	1.8977	CALM1//CALM2//CHUK//GAB1//GNG5//PAK1//PAK2//PAK3//RAB5A//RAP1B//RASA1//RASA2//RASSF5
Tuberculosis	0.0128631	0.208592	1.890654	CALM1//CALM2//CAMK2D//CREB1//CREBBP//JAK2//NOD2//PPP3CB//PPP3R1//RAB5A//TLR4
ErbB signaling pathway	0.0130258	0.208592	1.885195	ARAF//CAMK2D//CDKN1B//GAB1//PAK1//PAK2//PAK3
GABAergic synapse	0.0130258	0.208592	1.885195	CACNA1C//CACNA1D//GABRB3//GLS//GNAI1//GNG5//NSF
FoxO signaling pathway	0.0135547	0.208592	1.86791	ARAF//CCNG2//CDKN1B//CHUK//CREBBP//FOXO3//PLK4//SMAD4//SOD2
RNA transport	0.0183175	0.2634599	1.737134	EIF2S1//NUP205//NUP35//NUP54//PABPC1//PNN//SRRM1//THOC1//UPF2//XPO1
Melanogenesis	0.0190223	0.2634599	1.720735	CALM1//CALM2//CAMK2D//CREB1//CREBBP//GNAI1//WNT3
Neurotrophin signaling pathway	0.0231756	0.2918028	1.634968	CALM1//CALM2//CAMK2D//FOXO3//GAB1//RAP1B//RPS6KA5//TP53
Serotonergic synapse	0.0231756	0.2918028	1.634968	ARAF//CACNA1C//CACNA1D//DUSP1//GABRB3//GNAI1//GNG5//KCNN2
RNA degradation	0.0245590	0.2957768	1.609788	BTG1//CNOT2//CNOT6L//DHX36//PABPC1//TOB1

ESM Table 3

Pathway analysis of the genes differentially expressed between 3- and 12-month-ol rats

Peroxisome	0.0273407	0.3155575	1.56319	ACSL3//AMACR//IDH1//PEX1//PEX3//SOD2
Vasopressin-regulated water reabsorption	0.0294534	0.3263445	1.530863	AQP4//CREB1//NSF//RAB5A
Glioma	0.0333381	0.339407	1.477058	ARAF//CALM1//CALM2//CAMK2D//TP53
Ribosome biogenesis in eukaryotes	0.0351853	0.339407	1.453638	SBDS//TAF9//UTP15//UTP18//WDR75//XPO1
Measles - Rattus norvegicus (rat)	0.0360125	0.339407	1.443546	CDKN1B//CHUK//EIF2S1//JAK2//RCHY1//TACR1//TLR4//TP53
Cholinergic synapse - Rattus norvegicus (rat)	0.0363423	0.339407	1.439587	CACNA1C//CACNA1D//CAMK2D//CREB1//GNAI1//GNG5//JAK2
Basal transcription factors	0.0367588	0.339407	1.434638	GTF2A2//GTF2E1//TAF2//TAF9
Prostate cancer	0.0386906	0.3457199	1.412394	ARAF//CDKN1B//CHUK//CREB1//CREBBP//TP53
Glutamatergic synapse	0.0427453	0.3639458	1.369111	CACNA1C//CACNA1D//GLS//GNAI1//GNG5//PPP3CB//PPP3R1
HIF-1 signaling pathway	0.0462046	0.3639458	1.335314	CAMK2D//CDKN1B//CREBBP//HIF1A//PFKFB2//TCEB1//TLR4
Protein processing in endoplasmic reticulum	0.0468010	0.3639458	1.329744	CANX//EIF2S1//ERO1L//LMAN1//NGLY1//SEC23A//TRAM1//UBE2D1//UBE2G1
Transcriptional misregulation in cancer	0.048281	0.3639458	1.316224	BMI1//CDKN1B//DUSP6//HHEX//IGFBP3//MLLT3//PRCC//SMAD1//TP53
Proteoglycans in cancer	0.0492657	0.3639458	1.307455	ANK3//ARAF//CAMK2D//GAB1//HIF1A//PAK1//ROCK2//SDC2//TLR4//TP53//WNT3
