# Supplementary material for: Role of microRNAs in the age-associated decline of pancreatic beta cell function in rat islets
Source: Diabetologia. 2015 Oct 16;59(1):161–9. doi: 10.1007/s00125-015-3783-5 (PMC4670458; doi:10.1007/s00125-015-3783-5)
Supplement: Supplementary file 10 — (PDF 1047 kb) [file 125_2015_3783_MOESM10_ESM.pdf]

ESM Table 4

Analysis of microRNA changes occurring in the islets of one year-old rats

100 ng of RNA from the islets of 4 one-year-old (old) and 3 three month-old (young) rats was analyzed by microarray.
Signal intensity is given on a Log2 scale after quantile normalization of the data.

False Discovery Rates were calculated using the Benjamini-Hochberg procedure.
The microRNAs that were further investigated in the paper are highlighted.

MicroRNA	Fold Change	mean old	mean young	p-Value	Adjusted p-Value	old_01	old_02	old_3	old_4	young_1	young_2	young_3
rno-miR-383	4.18378693	7.177008	5.112199	9.98E-07	0.0002568	7.221566	6.928196	7.147593	7.410678	5.315344	5.18246	4.838792
rno-miR-130b	-3.24536646	3.92518	5.623561	1.67E-06	0.0002568	4.051069	3.689792	3.965546	3.994311	5.788084	5.420248	5.66235
rno-miR-488	2.11041673	4.383552	3.306024	6.20E-06	0.000634	4.429343	4.35494	4.320581	4.429343	3.270473	3.341759	3.30584
rno-miR-124	6.83709803	5.843526	3.070142	0.0004282	0.0328611	5.975075	4.429343	7.023825	5.945863	3.058408	3.148472	3.003546
rno-miR-29b	1.56973986	14.83601	14.18548	0.0006039	0.0370766	14.80544	14.80544	14.92773	14.80544	14.09774	14.36098	14.09774
rno-miR-129-1*	1.83605191	9.803542	8.926936	0.0011662	0.0495753	9.691077	9.624007	9.997914	9.901172	9.140748	9.106158	8.533901
rno-miR-181a	-2.06632734	6.562357	7.609426	0.0012919	0.0495753	6.376406	6.414711	7.07441	6.383901	7.439699	7.410678	7.977899
rno-miR-484	-2.34769639	5.139005	6.370251	0.0012119	0.0495753	4.953488	4.903962	5.847905	4.850667	6.27502	6.173038	6.662696
rno-miR-214	2.27042059	7.11889	5.93593	0.0015272	0.0520952	6.551795	7.306292	7.633774	6.983697	6.083659	6.116408	5.607724
rno-miR-199a-5p	2.34751205	7.019042	5.787909	0.0021415	0.0657436	6.591661	7.253131	7.568679	6.662696	5.99643	6.051954	5.315344
rno-miR-218a	1.60660382	6.175224	5.49121	0.0033574	0.0815319	5.927395	5.99643	6.335207	6.441864	5.325341	5.686493	5.461795
rno-miR-3544	-1.42454277	5.167813	5.678312	0.003277	0.0815319	5.141867	5.204696	5.103379	5.22131	5.686493	5.500537	5.847905
rno-let-7b*	-1.48476096	5.083133	5.653363	0.0037181	0.0815319	5.204696	5.240497	4.8279	5.059438	5.645251	5.526755	5.788084
rno-miR-702-3p	-1.54031688	5.458494	6.081721	0.0036973	0.0815319	5.500537	5.461795	5.28772	5.583923	6.013357	5.847905	6.383901
rno-miR-2985	-1.56776125	5.866944	6.51565	0.0041504	0.0849444	5.945863	5.891146	5.703372	5.927395	6.441864	6.238007	6.867079
rno-miR-129-2*	1.72446879	10.35229	9.566136	0.0052306	0.0888361	10.28066	10.13345	10.34061	10.65443	9.691077	9.901172	9.106158
rno-miR-425*	-1.39984022	4.375635	4.860897	0.0055715	0.0888361	4.409966	4.449917	4.172152	4.470504	4.8279	4.752127	5.002664
rno-miR-92b	-1.46813725	4.531089	5.085076	0.0052033	0.0888361	4.529641	4.632032	4.273043	4.689639	5.082727	4.918199	5.254302
rno-miR-19a	-1.69177525	6.205565	6.964102	0.005243	0.0888361	6.051954	6.041377	6.574131	6.154797	6.629245	6.928196	7.334866
rno-miR-19b	-1.86836244	8.719861	9.621636	0.0057874	0.0888361	8.421676	8.533901	9.348618	8.575251	9.348618	9.573971	9.942318
rno-miR-15b	-1.68335677	8.914011	9.665352	0.0068163	0.0996471	8.801041	8.723606	9.02524	9.106158	9.314833	9.520108	10.16111
rno-miR-539	1.47187886	5.248758	4.6911	0.0078882	0.1057553	5.420248	5.325341	4.9341	5.315344	4.752127	4.850667	4.470504
rno-miR-409-3p	-1.3703779	5.5769	6.031474	0.007923	0.1057553	5.63076	5.63076	5.383729	5.66235	6.030238	5.891146	6.173038

rno-miR-199a-3p	2.16117112	8.562508	7.450695	0.0092761	0.1132668	7.928395	8.969388	9.226254	8.125996	7.523711	7.753963	7.07441
rno-miR-142-5p	1.73435615	5.041736	4.247336	0.0126646	0.1132668	4.596469	5.686493	5.024167	4.859815	4.50573	4.205025	4.031253
rno-miR-34b	1.56619034	5.082546	4.435286	0.0110973	0.1132668	5.402038	4.9341	4.838792	5.155253	4.247351	4.230607	4.8279
rno-miR-330*	1.4536175	4.086283	3.546635	0.011472	0.1132668	4.273043	4.051069	3.826543	4.194475	3.78523	3.581583	3.273092
rno-miR-3563-3p	1.41565941	4.403343	3.901868	0.0129132	0.1132668	4.580894	4.34453	4.158304	4.529641	3.98023	4.074274	3.651101
rno-miR-126*	1.35212777	5.577149	5.141917	0.0120695	0.1132668	5.461795	5.66235	5.461795	5.722654	5.127335	5.315344	4.983072
rno-miR-29a	1.31811983	14.16355	13.76507	0.0121494	0.1132668	14.36098	14.09774	14.09774	14.09774	13.71472	13.86575	13.71472
rno-miR-1*	-1.36988721	5.000648	5.454706	0.0115354	0.1132668	5.103379	5.012181	4.903962	4.983072	5.420248	5.240497	5.703372
rno-miR-582*	-1.40687846	5.125328	5.617826	0.0127142	0.1132668	5.155253	5.22131	4.884254	5.240497	5.500537	5.461795	5.891146
rno-miR-3549	-1.40725182	5.222406	5.715287	0.0111934	0.1132668	5.28772	5.27116	5.043026	5.28772	5.583923	5.54858	6.013357
rno-miR-668	-1.40772637	4.298746	4.792113	0.0099447	0.1132668	4.386943	4.326134	4.094966	4.386943	4.736845	4.596469	5.043026
rno-miR-3085	-1.41577292	5.819062	6.320652	0.0106577	0.1132668	5.686493	5.927395	5.891146	5.771215	6.202586	6.130125	6.629245
rno-miR-142-3p	1.81234968	7.493458	6.635597	0.0135548	0.1155923	7.023825	8.267033	7.410678	7.272297	6.843262	6.591661	6.471868
rno-miR-338	1.3490638	6.836873	6.404914	0.0155097	0.1218316	6.748943	6.713223	6.788751	7.096575	6.574131	6.320306	6.320306
rno-miR-1188-3p	-1.28446497	4.540744	4.901911	0.0162707	0.1218316	4.50573	4.569286	4.470504	4.617455	4.838792	4.807504	5.059438
rno-miR-298	-1.33552338	4.770879	5.188285	0.0159688	0.1218316	4.850667	4.884254	4.596469	4.752127	5.103379	5.059438	5.402038
rno-miR-191*	-1.35178763	4.965113	5.399981	0.0156889	0.1218316	5.059438	5.002664	4.795685	5.002664	5.349996	5.204696	5.645251
rno-miR-181b	-1.77261467	4.692067	5.517946	0.015637	0.1218316	4.326134	4.752127	5.204696	4.485312	5.18246	5.28772	6.083659
rno-miR-667	-1.26073396	4.790855	5.125119	0.019182	0.1402112	4.8279	4.8279	4.700117	4.807504	5.141867	5.012181	5.22131
rno-miR-217	-3.38332314	7.058676	8.817117	0.021189	0.1478415	5.607724	7.714975	8.497293	6.414711	8.681056	8.421676	9.348618
rno-miR-216b-5p	-3.58049422	5.67572	7.515879	0.020767	0.1478415	4.308455	6.215123	7.096575	5.082727	6.748943	7.377017	8.421676
rno-miR-34c	1.51816608	4.204259	3.601929	0.0222525	0.1518115	4.555837	4.018022	3.984409	4.258766	3.226371	3.573187	4.006229
rno-miR-139-5p	-1.82504072	3.586121	4.45405	0.023281	0.1553752	3.253048	3.182753	4.547564	3.361119	4.569286	4.437923	4.35494
rno-miR-499	1.41903583	5.669599	5.164688	0.0250931	0.1639059	5.86689	5.570363	5.349996	5.891146	5.240497	5.383729	4.869838
rno-miR-323	1.29600922	5.408156	5.03408	0.0281621	0.1662647	5.526755	5.402038	5.155253	5.54858	5.043026	5.155253	4.903962
rno-miR-328b-3p	-1.27407418	3.952405	4.301855	0.0280027	0.1662647	4.094966	4.074274	3.723028	3.917353	4.34453	4.280517	4.280517
rno-miR-18a	-1.28188534	4.182369	4.540637	0.0273914	0.1662647	4.128691	4.137326	4.326134	4.137326	4.449917	4.409966	4.762027
rno-miR-92a	-1.62607603	7.72782	8.429214	0.0279648	0.1662647	7.508718	7.568679	8.31017	7.523711	7.949734	8.497293	8.840616
rno-miR-216a	-4.02528896	8.049213	10.0583	0.0271359	0.1662647	6.441864	8.632249	9.901172	7.221566	9.520108	9.774448	10.88036
rno-miR-652	1.24097362	10.08679	9.775318	0.0291128	0.1686347	10.16111	10.11027	9.942318	10.13345	9.774448	9.860427	9.691077
rno-miR-381*	1.32104393	3.930122	3.528444	0.0297005	0.1688527	4.146035	3.994311	3.710537	3.869605	3.407547	3.759296	3.418488
rno-miR-145	1.4200581	4.973953	4.468003	0.0324203	0.1750498	4.752127	5.18246	4.918199	5.043026	4.649142	4.736845	4.018022
rno-miR-143	1.36291715	10.8436	10.39691	0.0329598	0.1750498	10.65443	11.03799	10.72962	10.95239	10.47824	10.65443	10.05806
rno-miR-219-5p	-1.38839527	6.608642	7.08206	0.0330713	0.1750498	6.687816	6.320306	6.713223	6.713223	6.763604	7.042878	7.439699
rno-miR-20b-5p	-1.50096574	6.044724	6.630615	0.032268	0.1750498	5.788084	5.945863	6.414711	6.030238	6.154797	6.713223	7.023825
rno-miR-26b	1.21670336	12.1357	11.85272	0.0361592	0.1881506	12.17531	12.07288	12.22171	12.07288	11.91213	11.87045	11.77559

rno-miR-770*	1.3638457	4.932947	4.485267	0.0381175	0.1950347	5.082727	4.983072	4.762027	4.903962	4.850667	4.485312	4.119821
rno-miR-221	1.2511288	5.809303	5.486073	0.0389436	0.195995	5.750296	5.703372	5.975075	5.80847	5.445872	5.66235	5.349996
rno-miR-378	-1.25359123	5.406879	5.732946	0.0406123	0.1975838	5.27116	5.445872	5.526755	5.383729	5.607724	5.645251	5.945863
rno-miR-202*	-1.28587573	4.834278	5.197029	0.0402169	0.1975838	4.736845	4.689639	5.082727	4.8279	5.204696	5.002664	5.383729
rno-miR-20a	-1.49476712	6.981332	7.561253	0.0411901	0.1975838	6.808489	6.748943	7.439699	6.928196	7.07441	7.680953	7.928395
rno-miR-195	1.4613613	9.074317	8.527004	0.0447214	0.1989777	8.681056	9.314833	9.424412	8.876968	8.533901	8.876968	8.170144
rno-miR-22*	1.33389301	5.906404	5.490761	0.0429758	0.1989777	6.130125	5.823635	5.570363	6.101493	5.63076	5.570363	5.27116
rno-miR-24-2*	1.33294049	3.971829	3.557216	0.0440445	0.1989777	3.98023	3.831888	3.98023	4.094966	3.821212	3.695567	3.15487
rno-miR-23a	1.22678081	11.3094	11.01452	0.0443458	0.1989777	11.13879	11.31776	11.39052	11.39052	10.95239	11.13879	10.95239
rno-miR-16	-1.26814145	11.37645	11.71916	0.0446605	0.1989777	11.39052	11.25691	11.21484	11.64352	11.64352	11.64352	11.87045
rno-miR-3584-3p	-1.25104864	4.33204	4.655178	0.0500535	0.2195203	4.35494	4.320581	4.214717	4.437923	4.517695	4.529641	4.918199
rno-miR-29c	1.26342493	13.42032	13.08298	0.0512648	0.2216661	13.42529	13.30489	13.5258	13.42529	13.30489	13.14729	12.79676
rno-miR-10a-5p	1.35118628	8.920939	8.486713	0.0524702	0.2220788	8.467839	9.226254	9.071114	8.918551	8.389541	8.681056	8.389541
rno-miR-376b-5p	1.27588976	6.050092	5.698588	0.05301	0.2220788	6.320306	6.013357	5.750296	6.116408	5.703372	5.80847	5.583923
rno-miR-186	-1.20290528	8.060154	8.326677	0.0542538	0.2220788	8.080092	7.928395	8.125996	8.106131	8.421676	8.216854	8.3415
rno-miR-290	-1.2664682	5.822941	6.163752	0.0542169	0.2220788	5.703372	6.030238	5.927395	5.63076	6.101493	6.013357	6.376406
rno-miR-33*	1.20584496	3.627176	3.357132	0.0569696	0.2301273	3.749924	3.456581	3.651101	3.651101	3.345456	3.287312	3.438628
rno-miR-140	1.23516727	8.198909	7.894202	0.0617111	0.2460429	8.047962	8.047962	8.389541	8.31017	7.817323	8.047962	7.817323
rno-miR-130a	1.3817282	10.18531	9.718831	0.0626731	0.2461412	9.860427	10.34061	10.54227	9.997914	9.47225	10.11027	9.573971
rno-miR-377	1.32859648	4.65316	4.243257	0.06372	0.2461412	4.918199	4.649142	4.308455	4.736845	4.429343	4.402696	3.897733
rno-miR-382*	1.23484917	5.947997	5.643662	0.064141	0.2461412	6.083659	5.847905	5.80847	6.051954	5.722654	5.788084	5.420248
rno-miR-369-3p	1.31724354	4.618594	4.221072	0.0666142	0.2503607	4.884254	4.665679	4.258766	4.665679	4.326134	4.429343	3.90774
rno-miR-450a	1.2202172	4.945561	4.658423	0.0668716	0.2503607	4.807504	4.953488	5.18246	4.838792	4.665679	4.762027	4.547564
rno-miR-133b	1.30229468	5.021982	4.640926	0.0748719	0.2769358	4.795685	4.859815	5.012181	5.420248	4.580894	4.903962	4.437923
rno-miR-183*	1.25868012	4.199638	3.867726	0.0766151	0.2800098	4.377547	4.230607	3.917353	4.273043	3.90774	4.084673	3.610766
rno-miR-494	-2.04701983	8.323455	9.35698	0.081321	0.2937122	8.497293	7.977899	8.601774	8.216854	10.88036	8.389541	8.801041
rno-miR-434*	1.24859406	5.555663	5.235358	0.085565	0.3036416	5.771215	5.526755	5.22131	5.703372	5.402038	5.22131	5.082727
rno-miR-365	-1.23595079	7.382794	7.688415	0.0860483	0.3036416	7.306292	7.463035	7.508718	7.253131	7.463035	7.604778	7.997433
rno-miR-193	-1.5503342	7.100873	7.733453	0.0889888	0.3104495	6.843262	7.410678	7.306292	6.843262	7.377017	7.221566	8.601774
rno-miR-342-5p	1.21667746	4.367792	4.084846	0.0948482	0.3271729	4.547564	4.247351	4.128691	4.547564	3.965546	4.214717	4.074274
rno-miR-134	1.47306823	4.510088	3.951264	0.100936	0.3405204	4.172152	4.736845	5.002664	4.128691	4.185123	4.35494	3.313728
rno-miR-93	-1.19623939	6.600634	6.85914	0.1006409	0.3405204	6.713223	6.376406	6.490625	6.82228	6.867079	6.867079	6.843262
rno-miR-140*	1.20612979	7.798629	7.528244	0.1080658	0.3606108	7.768578	7.680953	7.852739	7.892247	7.783261	7.548341	7.253131
rno-miR-216b-3p	-1.35898918	3.6172	4.059734	0.1093404	0.360941	3.266238	3.793345	3.939962	3.469257	3.658347	3.939962	4.580894
rno-miR-362	1.1770357	3.580731	3.345573	0.1125046	0.3635675	3.610766	3.407547	3.581583	3.723028	3.484869	3.361119	3.190731
rno-miR-200c	-1.16922197	10.99126	11.21681	0.1114978	0.3635675	11.16633	10.88036	10.88036	11.03799	11.31776	11.16633	11.16633

rno-miR-497	1.35067847	8.620033	8.186349	0.1138418	0.3640566	8.187568	8.756301	9.002362	8.533901	8.31017	8.533901	7.714975
rno-miR-664-2*	1.23762812	3.978959	3.671381	0.1221223	0.3865108	4.185123	3.860413	3.852279	4.018022	3.994311	3.702025	3.317808
rno-miR-542-3p	1.17801058	4.132017	3.895664	0.1235934	0.3871751	3.984409	3.984409	4.34453	4.214717	3.984409	3.78523	3.917353
rno-miR-150	1.2167158	6.300421	6.017428	0.132452	0.3909883	6.116408	6.506282	6.202586	6.376406	5.945863	6.335207	5.771215
rno-miR-384-3p	1.17827594	4.629593	4.392916	0.1321751	0.3909883	4.859815	4.580894	4.377547	4.700117	4.402696	4.449917	4.326134
rno-let-7d	1.17074976	11.7712	11.54377	0.1305438	0.3909883	11.91213	11.77559	11.52663	11.87045	11.46114	11.52663	11.64352
rno-let-7a-1*	-1.15447576	3.912187	4.119425	0.1287806	0.3909883	3.929721	3.821212	4.018022	3.879794	4.137326	3.973598	4.247351
rno-miR-212	-1.18409153	5.780982	6.024763	0.128205	0.3909883	5.891146	5.722654	5.686493	5.823635	5.847905	5.927395	6.298987
rno-miR-378*	-1.20217738	4.095845	4.361494	0.1277368	0.3909883	4.084673	4.194475	4.119821	3.984409	4.074274	4.34453	4.665679
rno-miR-3557-3p	1.73689222	4.665786	3.869278	0.1351959	0.3952869	3.78523	5.127335	5.63076	4.119821	3.860413	4.580894	3.166527
rno-miR-505	1.15904205	5.058696	4.845763	0.1399792	0.4054114	5.024167	5.155253	4.850667	5.204696	4.795685	4.9341	4.807504
rno-miR-379*	1.15682213	3.86491	3.654743	0.1436654	0.412199	4.018022	3.826543	3.77048	3.844594	3.641559	3.821212	3.501456
rno-miR-10b	1.23874358	7.653952	7.345075	0.1463066	0.4125234	7.358678	7.852739	7.377017	8.027375	7.195173	7.463035	7.377017
rno-miR-29a*	1.13727058	3.894881	3.709306	0.146466	0.4125234	3.958541	3.958541	3.810164	3.852279	3.810164	3.627961	3.689792
rno-miR-134*	1.1638939	3.959354	3.740394	0.1500289	0.418717	4.074274	4.031253	3.667354	4.064535	3.77048	3.740166	3.710537
rno-miR-25	-1.16443071	8.69293	8.912555	0.1524356	0.4216012	8.723606	8.575251	8.632249	8.840616	8.756301	8.840616	9.140748
rno-miR-299	1.19864916	5.90671	5.6453	0.1617805	0.4425222	6.154797	5.975075	5.500537	5.99643	5.526755	5.722654	5.686493
rno-miR-505*	1.17057198	4.200143	3.972929	0.1635356	0.4425222	4.137326	4.280517	4.074274	4.308455	3.695567	4.051069	4.172152
rno-miR-337	1.13798986	8.027466	7.840978	0.1643242	0.4425222	8.106131	8.027375	7.928395	8.047962	7.892247	7.949734	7.680953
rno-miR-543*	1.19672698	5.03563	4.776536	0.1686134	0.446244	5.325341	5.024167	4.665679	5.127335	4.884254	4.8279	4.617455
rno-miR-17-5p	-1.15063599	5.106391	5.308822	0.1685007	0.446244	5.043026	5.043026	5.315344	5.024167	5.155253	5.445872	5.325341
rno-miR-376a*	1.1706758	4.417008	4.189666	0.1715334	0.4500919	4.632032	4.402696	4.230607	4.402696	4.320581	4.308455	3.939962
rno-miR-541*	1.17344277	4.136666	3.905919	0.1780358	0.4631949	4.320581	4.158304	3.776217	4.291564	3.885248	3.98023	3.852279
rno-miR-21	1.32537082	11.94155	11.53516	0.1831145	0.4645964	11.77559	11.64352	12.57152	11.77559	11.21484	12.07288	11.31776
rno-miR-487b	1.19972868	8.297618	8.03491	0.1811674	0.4645964	8.533901	8.497293	7.892247	8.267033	8.125996	8.125996	7.852739
rno-miR-423*	1.1477824	5.447484	5.248635	0.1817724	0.4645964	5.383729	5.420248	5.583923	5.402038	5.383729	5.349996	5.012181
rno-miR-411*	1.21097698	6.582787	6.306616	0.193116	0.4749662	6.867079	6.591661	6.083659	6.788751	6.290015	6.414711	6.215123
rno-miR-421*	1.2061751	5.341701	5.071262	0.1947322	0.4749662	5.583923	5.315344	4.859815	5.607724	4.983072	5.103379	5.127335
rno-miR-410	1.14908413	8.161157	7.960673	0.1909416	0.4749662	8.267033	8.240293	7.949734	8.187568	8.047962	8.080092	7.753963
rno-miR-30c-2*	1.13732426	5.358971	5.173327	0.1910304	0.4749662	5.445872	5.28772	5.240497	5.461795	5.012181	5.325341	5.18246
rno-miR-30c-1*	1.11006853	3.691296	3.540648	0.196891	0.4749662	3.667354	3.753235	3.68625	3.658347	3.545512	3.515463	3.560968
rno-miR-99b	-1.1644938	8.468258	8.68796	0.1976051	0.4749662	8.632249	8.3415	8.267033	8.632249	8.918551	8.513082	8.632249
rno-miR-144	-1.41842109	4.690027	5.194313	0.1980315	0.4749662	4.402696	4.485312	4.689639	5.18246	5.823635	4.258766	5.500537
rno-miR-483*	1.63138088	6.945383	6.239289	0.2026632	0.482307	6.238007	7.272297	8.187568	6.083659	6.383901	6.763604	5.570363
rno-miR-877	1.28534571	4.166715	3.804559	0.208213	0.491703	3.805028	4.470504	4.632032	3.759296	3.759296	4.185123	3.469257
rno-miR-30a	-1.11841152	9.990444	10.15189	0.2146012	0.5029203	9.997914	9.860427	10.16111	9.942318	10.16111	10.16111	10.13345

rno-miR-210	-1.12443058	7.889278	8.058473	0.2173763	0.5055646	8.027375	7.768578	7.783261	7.977899	8.170144	7.977899	8.027375
rno-miR-152	1.165747	8.818995	8.59774	0.2196328	0.5069719	8.951434	8.951434	8.421676	8.951434	8.467839	8.601774	8.723606
rno-miR-127*	1.19775216	6.043637	5.783308	0.2289616	0.5206756	6.335207	6.101493	5.607724	6.130125	6.051954	5.771215	5.526755
rno-miR-32	1.11062668	4.389238	4.237864	0.2282876	0.5206756	4.449917	4.291564	4.437923	4.377547	4.128691	4.326134	4.258766
rno-miR-341	1.15531618	7.84857	7.640283	0.2355988	0.5233705	7.997433	7.892247	7.651862	7.852739	7.928395	7.633774	7.358678
rno-miR-27a	1.15350176	10.36948	10.16346	0.2357737	0.5233705	10.13345	10.47824	10.65443	10.21182	9.997914	10.28066	10.21182
rno-miR-200b*	1.14725207	4.682785	4.484603	0.2377301	0.5233705	4.569286	4.529641	4.983072	4.649142	4.280517	4.617455	4.555837
rno-miR-106b	-1.1176142	8.79202	8.952442	0.2403754	0.5233705	8.840616	8.601774	8.756301	8.969388	8.969388	8.918551	8.969388
rno-miR-483	-1.11985118	4.790663	4.95397	0.2376141	0.5233705	4.762027	4.850667	4.953488	4.596469	5.024167	4.884254	4.953488
rno-miR-3547	-1.13287164	4.269891	4.449875	0.2390788	0.5233705	4.205025	4.273043	4.429343	4.172152	4.386943	4.273043	4.689639
rno-miR-146b	1.16021845	6.540351	6.325954	0.2450569	0.529806	6.763604	6.335207	6.298987	6.763604	6.130125	6.357113	6.490625
rno-miR-204	1.15147811	9.485523	9.282036	0.2482051	0.5328598	9.624007	9.348618	9.278389	9.691077	9.02524	9.348618	9.47225
rno-miR-381	1.14127447	7.962365	7.771719	0.2512999	0.5357574	8.125996	8.080092	7.714975	7.928395	7.977899	7.768578	7.568679
rno-miR-872	1.14855475	6.148948	5.949128	0.2545659	0.5389774	6.298987	6.051954	5.945863	6.298987	5.66235	6.030238	6.154797
rno-miR-29c*	1.12117349	8.113711	7.948701	0.2600189	0.546752	8.216854	7.949734	8.047962	8.240293	8.080092	7.997433	7.768578
rno-miR-598-3p	1.17943944	8.083313	7.845212	0.2641806	0.5517241	8.389541	7.997433	7.604778	8.3415	7.768578	7.817323	7.949734
rno-miR-34a	1.30112516	9.478683	9.098924	0.2910818	0.5617807	9.278389	9.691077	9.774448	9.170818	8.575251	8.723606	9.997914
rno-miR-203	1.28592228	4.824706	4.461902	0.2831414	0.5617807	4.689639	4.377547	5.66235	4.569286	4.064535	4.470504	4.850667
rno-miR-3588	1.26855503	4.397403	4.054217	0.29217	0.5617807	4.194475	4.094966	5.141867	4.158304	3.958541	4.569286	3.634824
rno-miR-337*	1.17290453	6.115894	5.885808	0.2806369	0.5617807	6.27502	6.083659	5.86689	6.238007	6.215123	5.99643	5.445872
rno-miR-411	1.16238297	6.694491	6.477406	0.2908056	0.5617807	7.042878	6.651578	6.27502	6.808489	6.651578	6.490625	6.290015
rno-miR-7a-1*	1.14915901	6.044188	5.84361	0.2814799	0.5617807	6.290015	5.788084	5.823635	6.27502	5.750296	5.750296	6.030238
rno-miR-380*	1.13387347	5.126907	4.945647	0.2809957	0.5617807	5.315344	5.059438	4.807504	5.325341	4.9341	5.043026	4.859815
rno-miR-29b-2*	1.12337526	3.344831	3.176992	0.2809309	0.5617807	3.565006	3.270473	3.100858	3.44299	3.300313	3.138324	3.092338
rno-miR-151	1.11665187	9.074564	8.915384	0.2839665	0.5617807	9.02524	9.02524	8.969388	9.278389	8.723606	8.951434	9.071114
rno-miR-329	1.10704401	8.365577	8.218865	0.2887422	0.5617807	8.513082	8.389541	8.170144	8.389541	8.240293	8.31017	8.106131
rno-miR-300-5p	1.09629875	4.455812	4.323171	0.2887551	0.5617807	4.470504	4.386943	4.409966	4.555837	4.377547	4.386943	4.205025
rno-let-7d*	-1.10749419	4.516629	4.663928	0.2927847	0.5617807	4.700117	4.50573	4.35494	4.50573	4.762027	4.700117	4.529641
rno-miR-30d	-1.20777248	9.420936	9.693285	0.2810126	0.5617807	9.47225	9.424412	9.314833	9.47225	10.34061	9.424412	9.314833
rno-miR-495	1.16087209	6.735371	6.520162	0.3011208	0.5741869	7.096575	6.687816	6.290015	6.867079	6.551795	6.651578	6.357113
rno-miR-325-5p	1.17899333	5.423196	5.18564	0.304287	0.5766427	5.722654	5.349996	4.869838	5.750296	5.28772	5.127335	5.141867
rno-miR-31*	1.12831926	5.969206	5.795031	0.3124896	0.5849653	6.215123	5.645251	6.041377	5.975075	5.80847	5.945863	5.63076
rno-miR-96	1.11653644	11.37173	11.21269	0.3113758	0.5849653	11.52663	11.46114	11.03799	11.46114	11.16633	11.21484	11.25691
rno-miR-99b*	1.13141915	4.018198	3.840065	0.323545	0.6019899	3.885248	4.084673	4.137326	3.965546	4.146035	3.885248	3.488911
rno-miR-324-3p	-1.09230849	7.862544	7.989924	0.3300464	0.6103871	7.977899	7.753963	7.768578	7.949734	7.997433	7.892247	8.080092
rno-miR-375	-1.09686772	13.51754	13.65093	0.3410903	0.6241034	13.5258	13.55953	13.42529	13.55953	13.86575	13.46855	13.61848

rno-miR-3546	-1.14908658	4.402093	4.60258	0.3415289	0.6241034	4.280517	4.517695	4.529641	4.280517	5.059438	4.517695	4.230607
rno-miR-344a-3p	1.15846857	7.260103	7.047884	0.3480781	0.6323075	7.633774	7.291858	6.808489	7.306292	7.358678	6.82228	6.962695
rno-miR-129	1.13730581	8.836134	8.650514	0.3560036	0.639141	8.969388	8.681056	8.467839	9.226254	8.801041	8.575251	8.575251
rno-miR-7b	-1.15531083	10.43201	10.64029	0.3552196	0.639141	10.47824	10.16111	10.28066	10.80802	10.54227	10.34061	11.03799
rno-miR-101b	1.09220538	9.136237	9.008993	0.3646419	0.6446936	9.314833	9.170818	8.918551	9.140748	9.106158	8.969388	8.951434
rno-miR-99a	-1.09577854	11.09652	11.22848	0.3653964	0.6446936	11.03799	10.95239	11.13879	11.25691	11.03799	11.25691	11.39052
rno-miR-500	-1.10960925	7.568769	7.718821	0.3652934	0.6446936	7.783261	7.523711	7.253131	7.714975	7.852739	7.651862	7.651862
rno-miR-3564	1.19277102	4.147996	3.893679	0.3763624	0.6569593	3.810164	4.437923	4.752127	3.591769	3.973598	3.984409	3.723028
rno-miR-374	1.10813966	4.641593	4.493453	0.3766281	0.6569593	4.869838	4.547564	4.386943	4.762027	4.700117	4.377547	4.402696
rno-miR-328a*	-1.26626853	6.114111	6.454695	0.3814219	0.6615623	5.823635	6.357113	6.748943	5.526755	7.221566	6.215123	5.927395
rno-miR-451	-1.25772182	7.211728	7.542541	0.383897	0.6621145	6.962695	6.983697	7.117261	7.783261	8.106131	6.629245	7.892247
rno-miR-335	1.24166865	9.226555	8.914275	0.3962217	0.6716834	9.901172	9.140748	8.240293	9.624007	8.840616	9.02524	8.876968
rno-miR-7a	1.10438428	14.91462	14.77138	0.3953316	0.6716834	15.02584	15.02584	14.58097	15.02584	14.80544	14.92773	14.58097
rno-miR-126	1.08619981	10.92529	10.806	0.39675	0.6716834	10.72962	11.13879	10.95239	10.88036	10.80802	10.88036	10.72962
rno-miR-664	-1.0955438	5.592979	5.724627	0.3981967	0.6716834	5.645251	5.607724	5.54858	5.570363	6.041377	5.583923	5.54858
rno-miR-300-3p	1.08998113	8.133466	8.009163	0.4011638	0.6729906	8.240293	8.125996	7.997433	8.170144	8.216854	8.027375	7.783261
rno-miR-758	1.08319329	4.206103	4.090812	0.4097704	0.683693	4.291564	4.214717	3.973598	4.34453	4.119821	4.158304	3.994311
rno-miR-3541	1.12053282	4.784363	4.620178	0.4170644	0.689377	4.665679	4.807504	5.254302	4.409966	4.596469	4.632032	4.632032
rno-miR-339-3p	1.10004749	3.241313	3.103748	0.4176681	0.689377	3.358979	3.140778	2.941036	3.524461	3.107162	3.209062	2.995019
rno-miR-361	1.09307339	6.941477	6.813086	0.4287368	0.6964138	7.195173	6.867079	6.629245	7.07441	6.808489	6.808489	6.82228
rno-miR-125b-5p	-1.07237255	11.2663	11.3671	0.4270431	0.6964138	11.21484	11.21484	11.31776	11.31776	11.25691	11.31776	11.52663
rno-miR-141	-1.1353215	12.82473	13.00783	0.4263274	0.6964138	12.92837	12.92837	12.51381	12.92837	13.5258	12.79676	12.70092
rno-miR-1949	1.1133496	6.457763	6.302856	0.4550232	0.7340566	6.173038	6.662696	6.82228	6.173038	6.335207	6.471868	6.101493
rno-miR-200a	1.06539856	11.50546	11.41406	0.4566932	0.7340566	11.46114	11.39052	11.64352	11.52663	11.39052	11.39052	11.46114
rno-miR-3559-5p	1.14424149	6.316806	6.122415	0.4625452	0.7395905	6.383901	6.116408	6.215123	6.551795	5.570363	6.083659	6.713223
rno-miR-434	1.0852077	9.831981	9.71401	0.4671533	0.7410415	9.942318	9.901172	9.624007	9.860427	9.624007	9.997914	9.520108
rno-miR-153	-1.07106311	10.12608	10.22512	0.4682803	0.7410415	10.21182	9.997914	10.13345	10.16111	10.05806	10.21182	10.40548
rno-let-7f	1.05998855	13.61848	13.53443	0.4719797	0.7430655	13.61848	13.61848	13.61848	13.61848	13.42529	13.61848	13.55953
rno-miR-183	1.08544548	9.979365	9.861078	0.4790705	0.7503808	10.11027	10.05806	9.691077	10.05806	9.901172	10.05806	9.624007
rno-miR-211*	1.28063835	6.790681	6.433818	0.4862667	0.7577862	6.101493	7.377017	7.817323	5.86689	6.471868	6.962695	5.86689
rno-miR-3584-5p	-1.50764197	9.44909	10.04138	0.4926441	0.7638472	8.170144	10.80802	11.16633	7.651862	10.28066	9.942318	9.901172
rno-let-7a	1.06786738	13.50561	13.41087	0.5075386	0.7747723	13.46855	13.5258	13.55953	13.46855	13.14729	13.55953	13.5258
rno-miR-99a*	1.06315493	5.168655	5.080304	0.5148324	0.7747723	5.18246	5.082727	5.059438	5.349996	4.953488	5.082727	5.204696
rno-miR-103	-1.06780514	10.22279	10.31743	0.5032058	0.7747723	10.34061	10.21182	10.05806	10.28066	10.13345	10.47824	10.34061
rno-miR-301a	-1.07800534	8.916915	9.02528	0.5131573	0.7747723	9.106158	9.002362	8.533901	9.02524	9.071114	9.002362	9.002362
rno-miR-802	-1.09214077	3.907788	4.034947	0.5109694	0.7747723	3.776217	3.973598	3.90774	3.973598	3.749924	3.869605	4.485312

rno-miR-182	-1.12640713	6.187832	6.35956	0.5094929	0.7747723	6.471868	6.173038	5.771215	6.335207	6.901019	6.202586	5.975075
rno-miR-369-5p	1.06637075	7.770994	7.678285	0.5322184	0.7970294	7.949734	7.817323	7.548341	7.768578	7.548341	7.852739	7.633774
rno-miR-485	1.06573121	4.798805	4.706961	0.5374605	0.8009727	4.9341	4.762027	4.580894	4.918199	4.807504	4.795685	4.517695
rno-miR-136*	1.07576568	8.815551	8.710187	0.5410247	0.8023894	9.071114	8.876968	8.513082	8.801041	8.876968	8.756301	8.497293
rno-miR-874	1.09170599	4.246764	4.120179	0.5464976	0.8066095	4.158304	4.258766	4.485312	4.084673	4.555837	4.064535	3.740166
rno-miR-296*	1.09326599	6.869733	6.741088	0.5497578	0.807539	6.983697	6.629245	7.291858	6.574131	6.490625	6.748943	6.983697
rno-miR-30c	1.06968818	8.961003	8.863813	0.5653674	0.8265133	9.170818	8.801041	8.801041	9.071114	8.601774	9.071114	8.918551
rno-miR-541	1.08108604	5.033707	4.921225	0.5712586	0.8306088	5.22131	5.103379	4.555837	5.254302	5.002664	5.024167	4.736845
rno-miR-328a	-1.08863149	7.390962	7.513477	0.57358	0.8306088	7.680953	7.358678	6.843262	7.680953	7.568679	7.508718	7.463035
rno-miR-150*	1.08317124	5.348843	5.233582	0.5829611	0.8397962	5.254302	5.54858	5.722654	4.869838	5.27116	5.141867	5.28772
rno-miR-30e*	1.06892784	6.291265	6.195101	0.5853954	0.8397962	6.490625	6.154797	6.013357	6.506282	5.975075	6.27502	6.335207
rno-miR-1249	1.12457407	7.20419	7.034811	0.5901586	0.8426916	7.117261	7.042878	8.027375	6.629245	6.788751	7.023825	7.291858
rno-miR-27b	1.07875703	12.54505	12.43568	0.5990444	0.8488742	12.79676	12.51381	12.07288	12.79676	12.22171	12.51381	12.57152
rno-miR-33	1.06690592	5.810857	5.717424	0.6119604	0.8488742	6.030238	5.383729	5.788084	6.041377	5.771215	5.63076	5.750296
rno-miR-136	1.06220895	9.724329	9.637262	0.6202246	0.8488742	9.774448	9.774448	9.573971	9.774448	9.942318	9.691077	9.278389
rno-miR-429	1.0527459	10.50705	10.43289	0.6098306	0.8488742	10.60861	10.40548	10.60861	10.40548	10.21182	10.60861	10.47824
rno-miR-22	1.04870795	12.27872	12.21011	0.6170536	0.8488742	12.38212	12.17531	12.17531	12.38212	12.38212	12.17531	12.07288
rno-let-7i	1.0422978	11.88087	11.8211	0.600325	0.8488742	11.87045	11.87045	11.87045	11.91213	11.77559	11.77559	11.91213
rno-miR-30e	1.04125953	9.548541	9.490211	0.6145845	0.8488742	9.573971	9.573971	9.47225	9.573971	9.573971	9.47225	9.424412
rno-miR-28	-1.05614979	6.015835	6.09465	0.622139	0.8488742	5.99643	5.750296	6.101493	6.215123	5.891146	6.154797	6.238007
rno-miR-345-5p	-1.05802224	6.443807	6.525177	0.6135475	0.8488742	6.662696	6.383901	6.238007	6.490625	6.320306	6.506282	6.748943
rno-miR-125a-3p	-1.12264874	5.980263	6.147169	0.6161609	0.8488742	5.54858	6.27502	6.651578	5.445872	6.591661	6.041377	5.80847
rno-miR-31	1.06983367	8.622276	8.524889	0.6271335	0.8506593	8.918551	8.216854	8.840616	8.513082	8.632249	8.632249	8.31017
rno-miR-652*	-1.13639262	8.04717	8.231631	0.6289891	0.8506593	7.523711	8.421676	8.951434	7.291858	8.187568	8.267033	8.240293
rno-miR-345-3p	-1.05523062	4.08294	4.160499	0.6354934	0.8556863	3.939962	4.128691	4.402696	3.860413	4.094966	4.094966	4.291564
rno-miR-326	1.0491332	4.518735	4.449537	0.6404114	0.8573217	4.485312	4.308455	4.649142	4.632032	4.35494	4.649142	4.34453
rno-miR-532-3p	1.04651905	4.710081	4.644482	0.6422931	0.8573217	4.838792	4.700117	4.50573	4.795685	4.632032	4.50573	4.795685
rno-miR-181c	1.0529573	6.496003	6.421556	0.6530595	0.8679189	6.651578	6.490625	6.551795	6.290015	6.116408	6.574131	6.574131
rno-miR-674-3p	1.05042857	4.054448	3.98347	0.6622081	0.876284	3.994311	4.064535	4.084673	4.074274	3.627961	4.137326	4.185123
rno-miR-3593-3p	1.08620234	6.31394	6.194647	0.6673077	0.8766707	6.041377	6.763604	6.662696	5.788084	6.376406	6.383901	5.823635
rno-miR-433*	1.04617134	4.856062	4.790943	0.6767784	0.8766707	5.002664	4.918199	4.569286	4.9341	4.689639	4.983072	4.700117
rno-miR-148b-3p	1.04182163	9.332267	9.273159	0.671597	0.8766707	9.424412	9.278389	9.106158	9.520108	9.278389	9.314833	9.226254
rno-miR-133a	-1.04368294	3.93694	3.998624	0.6735337	0.8766707	3.879794	3.852279	4.194475	3.821212	3.869605	4.146035	3.98023
rno-miR-101a	-1.05780527	9.373949	9.455023	0.676058	0.8766707	9.140748	9.520108	9.520108	9.314833	9.226254	9.278389	9.860427
rno-miR-342-3p	1.04612835	8.613789	8.548729	0.6871381	0.8863503	8.876968	8.513082	8.3415	8.723606	8.497293	8.467839	8.681056
rno-miR-455	1.04354297	7.703	7.64151	0.6934568	0.8907584	7.852739	7.783261	7.358678	7.817323	7.604778	7.714975	7.604778

rno-miR-370	1.10282114	6.708641	6.567442	0.7191193	0.9019309	6.357113	6.82228	7.334866	6.320306	7.291858	6.687816	5.722654
rno-miR-146a	1.07132869	7.842968	7.743567	0.726338	0.9019309	7.334866	8.467839	8.106131	7.463035	7.753963	7.928395	7.548341
rno-miR-205	1.063468	4.848082	4.759305	0.7208673	0.9019309	4.903962	4.205025	5.27116	5.012181	4.869838	4.838792	4.569286
rno-miR-7a-2*	1.06266354	7.102846	7.015161	0.7344714	0.9019309	7.568679	7.023825	6.441864	7.377017	6.928196	7.07441	7.042878
rno-miR-222	1.04921564	4.112649	4.043338	0.7077488	0.9019309	4.064535	3.953587	4.185123	4.247351	4.409966	4.018022	3.702025
rno-miR-352	1.04067859	7.200913	7.143388	0.7155973	0.9019309	7.410678	7.07441	6.983697	7.334866	6.962695	7.272297	7.195173
rno-miR-30b-5p	1.03109052	10.30964	10.26547	0.7318747	0.9019309	10.40548	10.28066	10.21182	10.34061	10.11027	10.40548	10.28066
rno-miR-223	-1.03436266	6.582361	6.631103	0.7337258	0.9019309	6.414711	6.574131	6.591661	6.748943	6.662696	6.441864	6.788751
rno-miR-363	-1.03930204	3.793623	3.849238	0.732318	0.9019309	3.505271	3.723028	4.006229	3.939962	3.826543	4.006229	3.714941
rno-miR-409-5p	-1.05190358	6.936483	7.009486	0.7158556	0.9019309	7.253131	6.962695	6.506282	7.023825	7.117261	7.147593	6.763604
rno-miR-188	-1.08103966	5.573329	5.685748	0.7311871	0.9019309	5.349996	5.771215	6.030238	5.141867	6.414711	5.402038	5.240497
rno-miR-125a-5p	1.03808296	9.17009	9.116169	0.7394374	0.9044114	9.348618	9.106158	8.876968	9.348618	8.951434	9.226254	9.170818
rno-miR-1224	1.13240735	11.96077	11.78138	0.7510503	0.9105635	11.31776	12.38212	12.92837	11.21484	12.51381	12.22171	10.60861
rno-miR-98	-1.02950413	9.992768	10.03472	0.7547328	0.9105635	10.05806	9.942318	9.860427	10.11027	9.860427	10.13345	10.11027
rno-miR-433	-1.05293727	6.745163	6.819583	0.7531125	0.9105635	7.07441	6.788751	6.154797	6.962695	7.023825	6.843262	6.591661
rno-miR-320	-1.05479497	6.539087	6.616049	0.7563312	0.9105635	6.901019	6.551795	6.051954	6.651578	7.042878	6.298987	6.506282
rno-miR-466b	1.05314008	4.703776	4.629079	0.7840018	0.921005	4.214717	4.617455	5.402038	4.580894	4.617455	4.859815	4.409966
rno-miR-325-3p	1.05186395	8.180786	8.107838	0.7815544	0.921005	8.575251	8.187568	7.463035	8.497293	8.027375	8.170144	8.125996
rno-miR-148b-5p	-1.03235941	3.726107	3.772052	0.7860043	0.921005	3.852279	3.776217	3.368193	3.90774	3.844594	3.591769	3.879794
rno-miR-362*	-1.03503832	6.395297	6.444981	0.7745056	0.921005	6.574131	6.298987	6.116408	6.591661	6.357113	6.290015	6.687816
rno-miR-379	-1.0397109	7.05596	7.112143	0.7785777	0.921005	7.291858	7.096575	6.687816	7.147593	7.410678	7.117261	6.808489
rno-miR-347	-1.04569728	5.681401	5.745866	0.7721997	0.921005	5.847905	5.583923	5.445872	5.847905	5.254302	5.86689	6.116408
rno-miR-137	-1.04946845	7.181303	7.250962	0.7851504	0.921005	7.604778	6.808489	6.763604	7.548341	7.147593	7.096575	7.508718
rno-miR-340-5p	-1.04257713	9.146669	9.206823	0.7892793	0.921326	9.520108	8.918551	8.723606	9.424412	9.424412	9.170818	9.02524
rno-miR-192	1.07036458	8.145714	8.047612	0.7951412	0.9246528	7.892247	7.439699	9.170818	8.080092	7.714975	8.240293	8.187568
rno-miR-194	1.05027943	6.862399	6.791626	0.8004965	0.9273677	6.82228	6.202586	7.523711	6.901019	6.82228	6.901019	6.651578
rno-let-7c	1.02222851	13.71472	13.68301	0.8116904	0.9368006	13.71472	13.71472	13.71472	13.71472	13.46855	13.71472	13.86575
rno-miR-425	-1.0279374	7.220991	7.260744	0.8157446	0.9379535	7.463035	7.195173	6.867079	7.358678	7.306292	7.358678	7.117261
rno-miR-3562	1.03647347	4.077577	4.025894	0.8232742	0.9401729	3.710537	4.172152	4.617455	3.810164	4.230607	3.917353	3.929721
rno-miR-200b	-1.03113628	10.88104	10.92528	0.8237997	0.9401729	10.80802	10.72962	11.25691	10.72962	10.60861	10.95239	11.21484
rno-miR-154*	1.02493652	6.274458	6.238923	0.8272967	0.940667	6.506282	6.238007	5.99643	6.357113	6.298987	6.376406	6.041377
rno-miR-384-5p	1.02719059	10.53538	10.49667	0.8362837	0.9438938	10.88036	10.60861	10.11027	10.54227	10.40548	10.54227	10.54227
rno-miR-128	-1.02853376	7.263459	7.304048	0.8337874	0.9438938	7.439699	7.117261	6.928196	7.568679	7.096575	7.291858	7.523711
rno-let-7b	1.02636398	13.22761	13.19006	0.8419637	0.9468236	13.14729	13.14729	13.46855	13.14729	12.79676	13.30489	13.46855
rno-miR-376c	-1.0239432	7.581131	7.615267	0.846667	0.9486378	7.817323	7.651862	7.221566	7.633774	7.651862	7.783261	7.410678
rno-miR-582	1.03714399	7.480879	7.428263	0.8623325	0.9596122	7.548341	7.334866	7.042878	7.997433	6.983697	7.253131	8.047962

rno-miR-154	1.02011083	8.995196	8.966471	0.865839	0.9596122	9.226254	9.071114	8.681056	9.002362	9.002362	9.140748	8.756301
rno-miR-466b-1*	-1.01689556	5.254606	5.278778	0.8649953	0.9596122	5.240497	5.254302	5.420248	5.103379	5.461795	5.27116	5.103379
rno-miR-324-5p	-1.0162426	8.432643	8.455888	0.8752752	0.9596767	8.601774	8.31017	8.216854	8.601774	8.513082	8.3415	8.513082
rno-miR-107	-1.01696066	10.65523	10.67949	0.8736073	0.9596767	10.95239	10.65443	10.40548	10.60861	10.65443	10.72962	10.65443
rno-miR-322	-1.02133155	7.236994	7.267445	0.8706742	0.9596767	6.928196	7.221566	7.680953	7.117261	7.334866	7.195173	7.272297
rno-miR-132	1.03591529	7.17869	7.127784	0.893103	0.9663406	7.377017	7.508718	6.320306	7.508718	7.633774	7.334866	6.414711
rno-miR-24	1.01674257	12.33714	12.31319	0.9046845	0.9663406	12.70092	12.22171	11.91213	12.51381	12.17531	12.38212	12.38212
rno-miR-23b	1.01466065	13.49103	13.47004	0.9065346	0.9663406	13.86575	13.42529	13.14729	13.5258	13.55953	13.42529	13.42529
rno-miR-466b-2*	-1.01299173	4.138107	4.156729	0.8919721	0.9663406	4.119821	3.98023	4.247351	4.205025	4.205025	4.291564	3.973598
rno-miR-127	-1.01734782	11.0994	11.12421	0.9014683	0.9663406	11.25691	11.16633	10.80802	11.16633	11.52663	11.03799	10.80802
rno-miR-340-3p	-1.02148965	6.701343	6.732018	0.9051394	0.9663406	7.147593	6.441864	6.173038	7.042878	6.506282	6.788751	6.901019
rno-miR-455*	-1.03108054	7.191515	7.235672	0.8968793	0.9663406	7.753963	6.901019	6.357113	7.753963	7.253131	7.306292	7.147593
rno-miR-139-3p	-1.0408163	6.051113	6.108828	0.903328	0.9663406	5.127335	6.843262	6.962695	5.27116	6.173038	6.101493	6.051954
rno-miR-382	1.01292927	7.537175	7.518642	0.9134511	0.9703443	7.714975	7.633774	7.195173	7.604778	7.680953	7.568679	7.306292
rno-miR-376a	-1.01367845	8.793633	8.813233	0.9186236	0.9724739	9.002362	8.840616	8.575251	8.756301	9.170818	8.801041	8.467839
rno-miR-350	1.00788142	3.83569	3.824365	0.9280529	0.9757268	3.917353	3.844594	3.695567	3.885248	3.897733	3.879794	3.695567
rno-miR-431	-1.01301723	7.39934	7.417998	0.9274917	0.9757268	7.651862	7.604778	6.901019	7.439699	7.508718	7.523711	7.221566
rno-miR-30a*	1.00945605	6.497744	6.484166	0.9340061	0.9786343	6.629245	6.290015	6.383901	6.687816	6.238007	6.662696	6.551795
rno-miR-532-5p	-1.00976987	6.045137	6.059164	0.9382822	0.9797709	6.202586	6.130125	5.645251	6.202586	5.927395	5.975075	6.27502
rno-miR-327	1.02510332	6.740228	6.704459	0.9452715	0.9837232	6.013357	7.548341	7.753963	5.645251	6.687816	6.983697	6.441864
rno-let-7e	1.00668663	11.99558	11.98596	0.9505144	0.9840189	12.07288	11.91213	11.77559	12.22171	11.87045	11.91213	12.17531
rno-miR-100	1.00586895	7.22184	7.213398	0.9551715	0.9840189	7.272297	7.147593	7.272297	7.195173	7.272297	7.439699	6.928196
rno-miR-331	1.00557638	8.229518	8.221495	0.9544574	0.9840189	8.31017	8.106131	8.080092	8.421676	8.3415	8.106131	8.216854
rno-miR-30d*	1.00366861	4.182137	4.176854	0.9736082	0.9930157	4.258766	4.185123	3.929721	4.35494	4.194475	3.958541	4.377547
rno-miR-26a	1.00294355	12.55652	12.55228	0.973029	0.9930157	12.57152	12.57152	12.38212	12.70092	12.57152	12.57152	12.51381
rno-miR-181d	-1.00427656	5.083634	5.08979	0.9696015	0.9930157	4.983072	5.141867	5.325341	4.884254	4.859815	5.254302	5.155253
rno-miR-3573-3p	-1.00412589	5.95843	5.96437	0.9776566	0.9938243	5.80847	5.86689	6.471868	5.686493	5.86689	5.823635	6.202586
rno-miR-672	-1.00699527	5.965146	5.975203	0.9808755	0.9938243	5.66235	5.80847	6.376406	6.013357	5.22131	5.607724	7.096575
rno-miR-30b-3p	1.00161249	4.893088	4.890763	0.9848603	0.9945793	5.012181	4.869838	4.736845	4.953488	4.918199	4.869838	4.884254
rno-miR-185	-1.00083153	8.239346	8.240545	0.9935516	0.9967985	8.3415	8.170144	7.977899	8.467839	8.267033	8.187568	8.267033
rno-miR-208*	-1.00125389	4.556686	4.558494	0.990678	0.9967985	4.617455	4.838792	4.449917	4.320581	4.470504	4.555837	4.649142
rno-miR-376b-3p	1.00041973	6.465653	6.465048	0.9976109	0.9976109	6.788751	6.471868	6.130125	6.471868	6.713223	6.551795	6.130125
