# Supplementary material for: Role of microRNAs in the age-associated decline of pancreatic beta cell function in rat islets
Source: Diabetologia. 2015 Oct 16;59(1):161–9. doi: 10.1007/s00125-015-3783-5 (PMC4670458; doi:10.1007/s00125-015-3783-5)
Supplement: Supplementary file 11 — (PDF 349 kb) [file 125_2015_3783_MOESM11_ESM.pdf]

**ESM Table 5**

**MicroRNA expression in different organs of 12-month old male Wistar rats.** RNA was isolated from brain, liver, skeletal muscle and adipose tissue of rats aged 3 and 12 months. MicroRNA expression was assessed by qRT-PCR. Values are expressed as fold change *versus* the level at 3 months (1 ± SD)

| ID | Brain | Liver | Skeletal muscle | Adipose tissue |
|---|---|---|---|---|
| miR-34a | 1.58 ± 0.01* | 10.89 ± 4.4* | 1.72 ± 0.35 | 0.27 ± 0.36 |
| miR-124a | 0.55 ± 0.22 | 0.58 ± 0.22 | 3.3 ± 1.5 | 3.5 ± 2 |
| miR-130b | 1 ± 0.46 | 1.15 ± 0.02 | 1.18 ± 0.02 | 0.43 ± 0.23 |
| miR-181a | 1.16 ± 0.11 | 1.2 ± 0.04 | 1.35 ± 0.02 | 1.72 ± 0.35 |
| miR-383 | 0.78 ± 0.27 | 0.58 ± 0.18 | 0.58 ± 0.16 | 1.28 ± 0.5 |

(n = 5). *Significantly different from the control (Student t-test $p<0.05$)
