# Supplementary material for: Role of microRNAs in the age-associated decline of pancreatic beta cell function in rat islets
Source: Diabetologia. 2015 Oct 16;59(1):161–9. doi: 10.1007/s00125-015-3783-5 (PMC4670458; doi:10.1007/s00125-015-3783-5)
Supplement: Supplementary file 12 — (PDF 303 kb) [file 125_2015_3783_MOESM12_ESM.pdf]

ESM Table 6

List of predicted miR-34 and miR-181 target genes upregulated in the islets of 12 month-old rats

microRNA family	Gene name	MRE for 3'UTR	microRNA family	Gene name	MRE for 3'UTR
miR-34 family	Ghr	1	miR-181 family	Rbm25	1
miR-34 family	RGD1359509	1	miR-181 family	Paics	1
miR-34 family	Gng5	2	miR-181 family	Rnpc2	2
miR-34 family	LOC100125362	2	miR-181 family	Trim2	3
miR-34 family	Rps6ka5	1	miR-181 family	Nr3c1	1
miR-34 family	Rab10	1	miR-181 family	Sfrs18	1
miR-34 family	Chd1	1	miR-181 family	Ggps1	1
miR-34 family	Cggbp1	1	miR-181 family	Pfkfb2	1
miR-34 family	Phf17	1	miR-181 family	Elf2	1
miR-34 family	Cdc42se2	2	miR-181 family	Hnrph1	1
miR-34 family	MGC108974	1	miR-181 family	RGD1559610	1
miR-34 family	Gls	1	miR-181 family	Rod1	1
miR-34 family	Api5	1	miR-181 family	Slc7a1	2
miR-34 family	Zcchc9	2	miR-181 family	Apoh	1
miR-34 family	Tnfrsf11b	1	miR-181 family	Spg3a	1
miR-34 family	Rybp	1	miR-181 family	Rcn2	1
miR-34 family	RGD1566036	1	miR-181 family	Mtm1	1
miR-34 family	RGD1308430	1	miR-181 family	RGD1562416	2
miR-34 family	Cnot6l	1	miR-181 family	Tjp1	1
miR-34 family	Trim2	1	miR-181 family	RGD1311558_pred	1
miR-34 family	Upf2	1	miR-181 family	Zbtb8b	1
miR-34 family	RGD1305117	1	miR-181 family	Gdap1	1
miR-34 family	Nr3c1	1	miR-181 family	Cnot6l	1
miR-34 family	RGD621098	1	miR-181 family	Cggbp1	2
miR-34 family	Txndc13	1	miR-181 family	Ehmt1	1
miR-34 family	Pfkfb2	1	miR-181 family	Trim35	1
miR-34 family	Nup35	1	miR-181 family	Slc24a2	1
miR-34 family	LOC680039	1	miR-181 family	Rbm22	1
miR-34 family	Ank3	1	miR-181 family	L2hgdh	4
miR-34 family	Amd1	2	miR-181 family	Dusp6	1
miR-34 family	RGD735106	1	miR-181 family	Ptgfrn	1
miR-34 family	Gab1	1	miR-181 family	Chordc1	1
miR-34 family	Nfat5	3	miR-181 family	Gls	2
miR-34 family	Aqp4	2	miR-181 family	RGD1305823	1
miR-34 family	RGD1560796	1	miR-181 family	Morc3	2
miR-34 family	Cttnbp2nl	1	miR-181 family	RGD735140	1
miR-34 family	Zfp26	1	miR-181 family	Lpl	1
miR-34 family	Aqp4	2	miR-181 family	Tlr4	1
miR-34 family	MGC114410	1	miR-181 family	Ccdc62	1
miR-34 family	Tmco3	1	miR-181 family	Gabrb3	1
miR-34 family	Slc48a1	1	miR-181 family	Crebbp	1
miR-34 family	MGC72992	1	miR-181 family	Nat5	1
miR-34 family	Zfp281	1	miR-181 family	Tbc1d14	2
miR-34 family	Topors	1	miR-181 family	Chd1	1

miR-34 family	Nuak2	1
miR-34 family	P2ry1	1
miR-34 family	Rexo4	1
miR-34 family	Rgs17	1
miR-34 family	RGD1560155	1
miR-34 family	Znf644	1
miR-34 family	Pdk3	1
miR-34 family	Dnajb4	1
miR-34 family	Pdik1l	1
miR-34 family	Ganc	1
miR-34 family	Usp9x	1
miR-34 family	Crbn	1
miR-34 family	Tnrc6b	2
miR-34 family	Arid1a	1
miR-34 family	C11orf8h	1
miR-34 family	Rkhd2	1
miR-34 family	Ngly1	1
miR-34 family	Tp53	1
miR-34 family	Nkiras1	1
miR-34 family	Pcmtd2	1
miR-34 family	Ttc5	1
miR-181 family	Dhx36	1
miR-181 family	Usp8	1
miR-181 family	Rab10	1
miR-181 family	RGD1307084	1
miR-181 family	Rkhd2	1
miR-181 family	Utp15	1
miR-181 family	Amt	1
miR-181 family	Ngly1	1
miR-181 family	Nab1	1
miR-181 family	Serpinb5	1
miR-181 family	Ythdf3	5
miR-181 family	Capn7	1
miR-181 family	Tmtc3	1
miR-181 family	Reck	1
miR-181 family	Rg9mtd1	1
miR-181 family	15.sept	2
miR-181 family	Lrrn1	1
miR-181 family	Pcgf6	1
miR-181 family	Il6ra	2
miR-181 family	Tbc1d14	2
miR-181 family	Tnrc6b	2
miR-181 family	Usp9x	1
miR-181 family	Elf2	1
miR-181 family	Synj1	1
miR-181 family	RGD1561797	1
miR-181 family	Actr3	1
miR-181 family	Arhgap12	1
miR-181 family	Herc3	2
miR-181 family	Plekha3	1
miR-181 family	Gnai1	1
miR-181 family	Ppp3r1	1
miR-181 family	Tceb1	2
miR-181 family	P2ry1	2
miR-181 family	Cwf19l2	1
miR-181 family	Calm1	1
miR-181 family	Cald1	1
miR-181 family	Slc7a5	1
miR-181 family	Galnt4	1
miR-181 family	Tbc1d14	2
miR-181 family	Gab1	1
miR-181 family	Hmgcs1	1
miR-181 family	Uba6	1
miR-181 family	Zmiz1	1
miR-181 family	RGD1305703	1
miR-181 family	Ube2a	2
miR-181 family	Zfp26	1
miR-181 family	Hdgfrp3	1
miR-181 family	Twistnb	1
miR-181 family	Nfat5	1
miR-181 family	Cttnbp2nl	1

miR-181 family	RGD1560796	2
miR-181 family	Canx	1
miR-181 family	Lats2	1
