# Supplementary material for: Role of microRNAs in the age-associated decline of pancreatic beta cell function in rat islets
Source: Diabetologia. 2015 Oct 16;59(1):161–9. doi: 10.1007/s00125-015-3783-5 (PMC4670458; doi:10.1007/s00125-015-3783-5)
Supplement: Supplementary file 13 — (PDF 237 kb) [file 125_2015_3783_MOESM13_ESM.pdf]

ESM Table 7

Predicted miR-34 and miR-181 target genes down-regulated in the islets of 12 month-old rats

microRNA family	Gene name	MRE for 3'UTR	microRNA family	Gene name	MRE for 3'UTR
miR-34 family	Prkab2	1	miR-181 family	Tek	1
miR-34 family	Tgfb3	1	miR-181 family	Myl1	1
miR-34 family	Alox15	1	miR-181 family	Cytl1	2
miR-34 family	Mrvi1	1	miR-181 family	Zcchc24	1
miR-34 family	Gpx7	1	miR-181 family	Syn2	1
miR-34 family	Mrvi1	1	miR-181 family	Gnat3	1
miR-34 family	Dpysl5	1	miR-181 family	Ddn	2
miR-34 family	Kcnk2	1	miR-181 family	Sall2	1
miR-34 family	Tor1b	1	miR-181 family	Grap	1
miR-34 family	RGD1560436	1	miR-181 family	Bcl6b	1
miR-34 family	Coq4	1	miR-181 family	Csrp1	1
miR-34 family	Serpinc1	1	miR-181 family	C1qtnf6	1
miR-34 family	Gm52_predicted	1	miR-181 family	Lysmd1	1
miR-34 family	Ucn2	1	miR-181 family	Myl1	1
miR-34 family	Bin2	1	miR-181 family	LOC294513	1
miR-34 family	Scly	1	miR-181 family	Fam84a	1
miR-34 family	Inexa	1	miR-181 family	Ccl9	2
miR-34 family	Casq2	1	miR-181 family	Eif2b5	1
miR-34 family	LOC500102	1	miR-181 family	Snx11	1
miR-34 family	Plekha8	1	miR-181 family	Dpysl5	1
miR-34 family	LOC690806	1	miR-181 family	Il22ra2	2
miR-34 family	Rhov	1	miR-181 family	Bspry	1
miR-34 family	Arf4l	1			
miR-34 family	Hr	2			
miR-34 family	Mmp17	1			
miR-34 family	Tek	1			
miR-34 family	Slc16a3	1			
miR-34 family	Gabrd	1			
miR-34 family	Cplx1	1			
miR-34 family	Npy1r	1			
miR-34 family	RGD1310269	1			
miR-34 family	Tmprss13	1			
miR-34 family	Vamp1	1			
miR-34 family	Il22ra2	1			
miR-34 family	Stard8	1			
miR-34 family	Prkag1	1			
miR-34 family	Sox10	1			
miR-34 family	Adamts15	1			
miR-34 family	Myo1f	1			
miR-34 family	nod3l	1			
miR-34 family	Samd14	1			
miR-34 family	Ninj1	1			
miR-34 family	Zc3hc1	1			
miR-34 family	Ros1	1			

miR-34 family	Spata2L	1
miR-34 family	Il4ra	1
